# Synthesis of novel sulphonamide derivatives from tunable quinolines with computational studies

**DOI:** 10.1038/s41598-025-94817-1

**Published:** 2025-03-31

**Authors:** Nagesh Dhanaji Chavan, S. Sarveswari, V. Vijayakumar

**Affiliations:** Department of Chemistry, School of Advanced Sciences, Vellore Institute of Technology, Vellore, 632014 India

**Keywords:** Synthesis, Quinoline, Sulphonamide–quinoline, UV–vis, Fluorescence, DFT, MEP, TD-DFT, Molecular docking, ADMET, Computational biology and bioinformatics, Chemistry

## Abstract

The synthesis of new quinoline-sulphonamide derivatives was accomplished through a meticulous five-step molecular assembly utilizing Suzuki, acid–amine cross-coupling reactions and N-alkylation. The integrity of each derivative was thoroughly confirmed via comprehensive spectroscopic analyses, including ^1^H and ^13^C NMR, DEPT-135, ^1^H-^1^H COSY, HSQC NMR and HRMS techniques. Subsequently, the absorbance and emission spectra of the newly synthesized derivatives were thoroughly investigated. Absorbance spectra were determined to be restricted within the range of 337 nm to 341.73 nm, with compound **10j** exhibiting the maximum wavelength of 341.73 nm; conversely, emission spectra were uniformly detected within the range of 411.70 nm to 429.90 nm upon excitation at 340 nm, with compound **10f** demonstrating the highest wavelength of 429.90 nm. Notably, these fluorophores displayed impressive characteristics, with high intensity and significant molar extinction coefficients; quantum yield ranging from 0.015 to 0.558 along with the highest stokes shifts in **10h** compound (0.6237 × 10^–4^) in acetonitrile solvent. Additionally, compound **10p** showed strong binding affinity and favorable pharmacokinetic properties through molecular docking studies and ADMET calculations. The electronic structure of the molecules was elucidated using techniques such as density functional theory (DFT) and molecular electrostatic potential (MEP) mapping. Additionally, the calculated global reactivity parameters provided valuable insights. Compound **10p** exhibited a distinctly low energy gap compared to other compounds, demonstrating its exceptional properties. The comparison between experimental and theoretical UV–vis spectra with major contribution transition in percentage also showcased the remarkable consistency and quality of the synthesized derivatives, highlighting the significant potential of this work in the field of fluorophore and biological application.

## Introduction

**Fig. 1:**
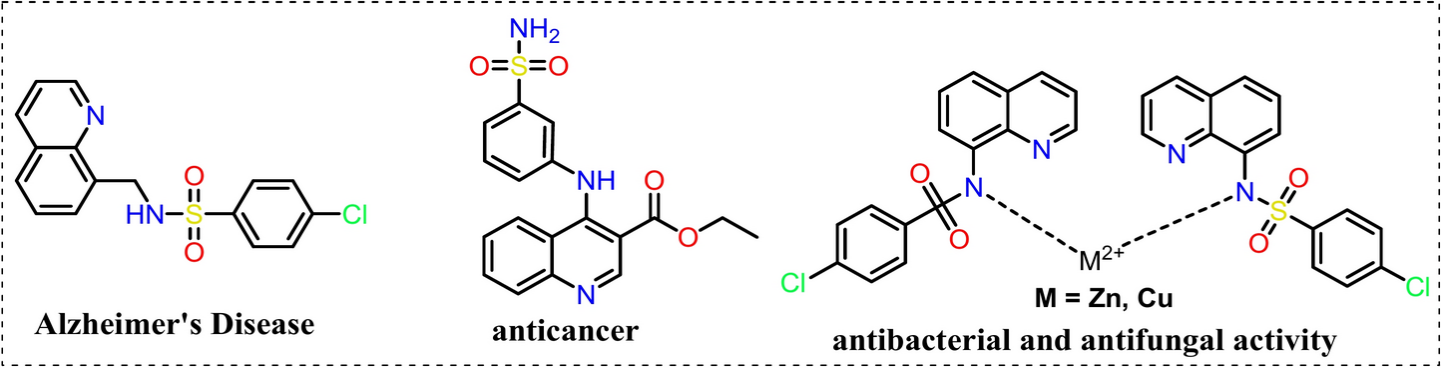
Biological activity on quinoline-sulphonamide derivatives.

Quinoline-sulphonated derivatives are important in several fields due to their unique photophysical, chemical, and biological properties. They are widely used as dyes, particularly in the textile and paper industries, due to their ability to form stable, intensely colored complexes^1,2^. These compounds are also valuable in analytical chemistry as pH indicators and in fluorescence-based assays, where their absorbance and emission characteristics can be finely tuned by modifying the structure of the quinoline ring or the position of the sulphonate group^3,4^. Quinoline-sulphonated compounds have found applications in medicinal chemistry also. They exhibit a broad spectrum of biological activities^5^, including antibacterial^6^, Alzheimer’s disease^7^, antifungal^8^, anticancer^9^, antimalarial^10^, tumor cell-specific M2 isoform of pyruvate kinase^11^, mycobacterium tuberculosis H37Rv strain inhibitors^12^, mycobacterial activity^13^, and antioxidant^14,15^. This makes them attractive candidates for drug development, particularly in the design of new therapeutics that target specific enzymes or cellular pathways (Fig. 1). Furthermore, the photophysical properties of quinoline-sulphonated compounds, such as their absorbance and fluorescence behavior, photoluminescent materials, as well as dyes utilized in organic light-emitting diodes (OLEDs) and solar cells are of significant interest in the study of molecular interactions and in the development of sensors and probes for detecting various analytes^16–18^. The sulphonate group not only enhances the water solubility of these compounds but also affects their electronic properties, making them versatile tools in both scientific research and industrial applications^19^. Their unique structural attributes facilitate specific interactions with proteins, nucleic acids, and metal ions, resulting in alterations in fluorescence that can be harnessed for visualizing cellular components, monitoring biological processes, or observing physiological parameters such as pH levels and reactive oxygen species^1,20^. The adaptability of quinoline-sulphonamide-based fluorophores renders them exceedingly efficacious in applications related to diagnostics, monitoring therapeutic interventions, and advancing fundamental research in biology^21,22^.

Computational chemistry, which is significantly propelled by the continuous evolution of cutting-edge technology and Sophisticated algorithms, underscores the pivotal role played by density functional theory (DFT) in the comprehensive analysis of electronic structures as well as the reactivity patterns present within various molecular systems, while simultaneously facilitating the prediction of intricate molecular characteristics and properties^23–25^. The application of DFT is grounded in the utilization of reactivity descriptors that include fundamental concepts such as electronegativity and hardness, which serve to enhance our understanding and predictive capabilities regarding molecular behavior and attributes in both the domains of chemistry and materials science^26–28^.

The primary objective of synthesizing this series of quinoline-sulphonamide derivatives was to investigate their photophysical attributes and theoretical characteristics through density functional theory (DFT) studies^29–31^. By analysing the absorbance, emission, and electronic structure, we sought to elucidate their prospective applications in the fields of optoelectronics, sensing, and coordination chemistry^32^. This holistic approach offers both experimental and computational perspectives into the structural and functional attributes of the synthesized derivatives. The design of the quinoline-sulphonamide derivatives was aimed at augmenting photophysical properties by exploiting the electron-withdrawing effects of the sulphonamide group. This enhancement leads to improved fluorescence, refined electronic structure, and facilitated molecular interactions. Moreover, the sulphonamide moiety increases binding affinity in docking investigations, thus rendering these derivatives invaluable for applications in optoelectronics, sensing, and prospective biological domains. Furthermore, one selected compound underwent molecular docking and absorption, distribution, metabolism, excretion, and toxicity (ADMET) studies to evaluate its interactions with biological targets and to forecast its pharmacokinetic properties, thereby augmenting its potential significance in drug development^33^. Numerous researchers have employed the synthesis of sulphonamides through the reaction of amines with sulphonyl chloride in the presence of a base, such as pyridine^34^, DIPEA, sodium hydroxide, and triethylamine^35^; therefore, in our experimental framework, we utilized triethylamine as the base, which resulted in a commendable yield.

In the current study, an advanced five-step synthetic protocol involving cyclization, reduction, acid–amine cross-coupling reactions, Suzuki cross-coupling, and alkylation was utilized to produce innovative quinoline-sulphonamide-derived fluorophores, achieving remarkable overall yields between 70 and 90%. Complete characterization utilizing NMR spectroscopy, High-Resolution Mass Spectrometry (HRMS), and computational assessments were performed to analyze the Frontier Molecular Orbital (FMO) characteristics, Global Reactivity, and Molecular Electrostatic Potential (MEP) of these compounds. The fluorescence characteristics, along with UV absorption and emission spectra, were systematically scrutinized, with emission spectra documented following excitation at a wavelength of 340 nm. Furthermore, among all derivatives, one specific compound underwent molecular docking against antibacterial-related proteins and an ADMET analysis based on DFT studies.

## Result and discussion

### Chemistry

**Scheme 1:**
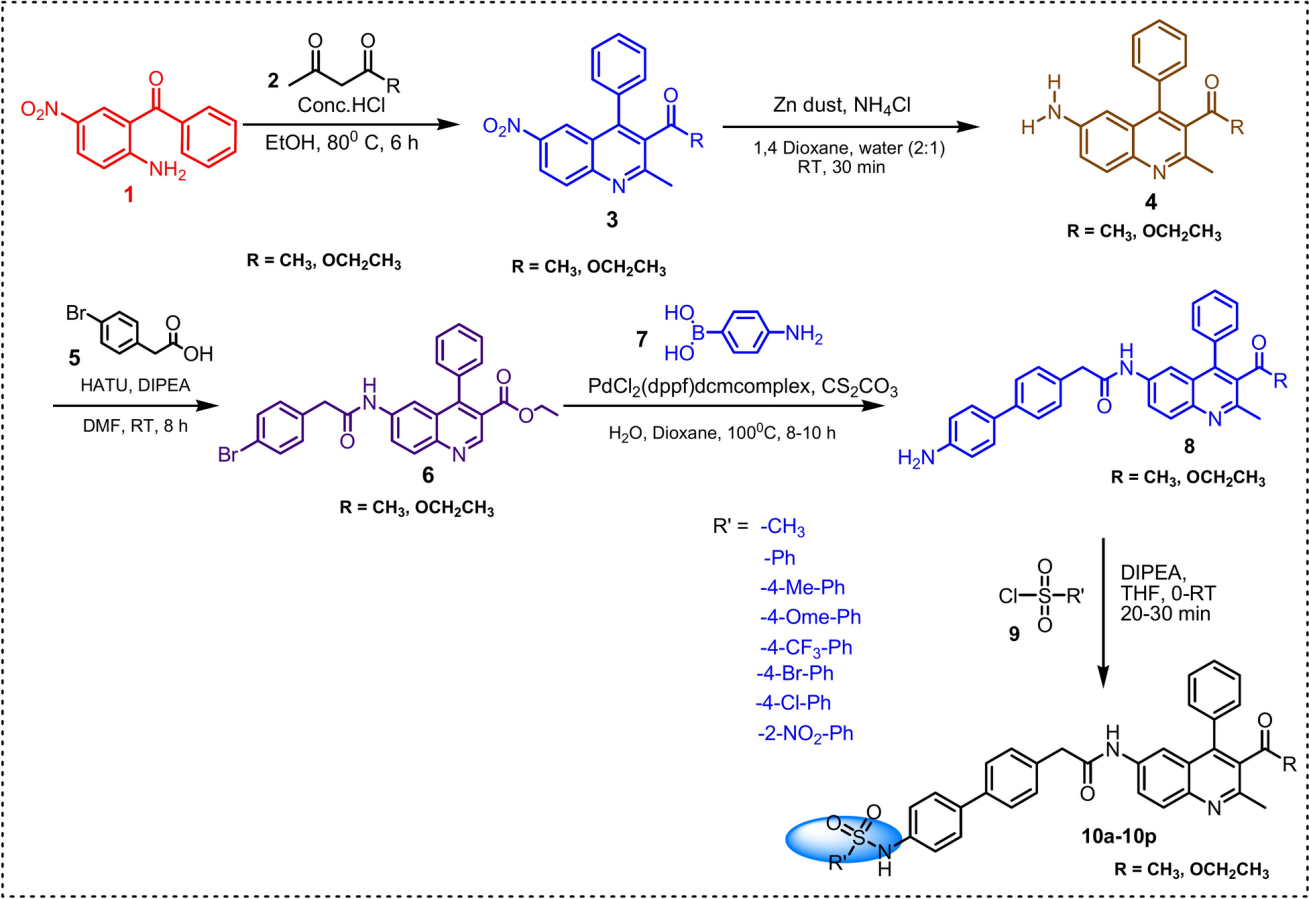
Synthesis route of novel quinoline-sulphonamide derivatives.

**Fig. 2:**
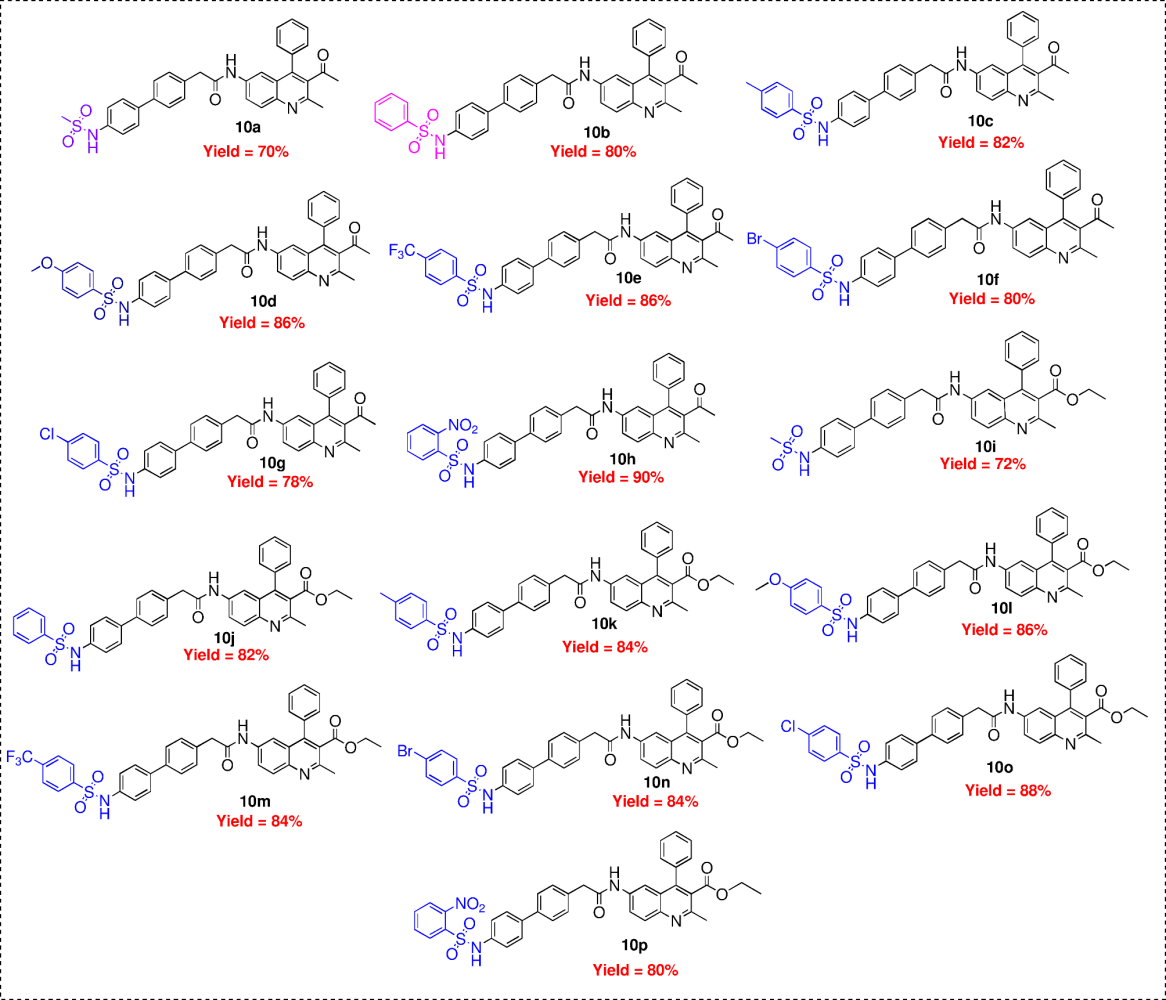
Synthesized derivatives of compounds (**10a**-**10p**).

**Fig. 3:**
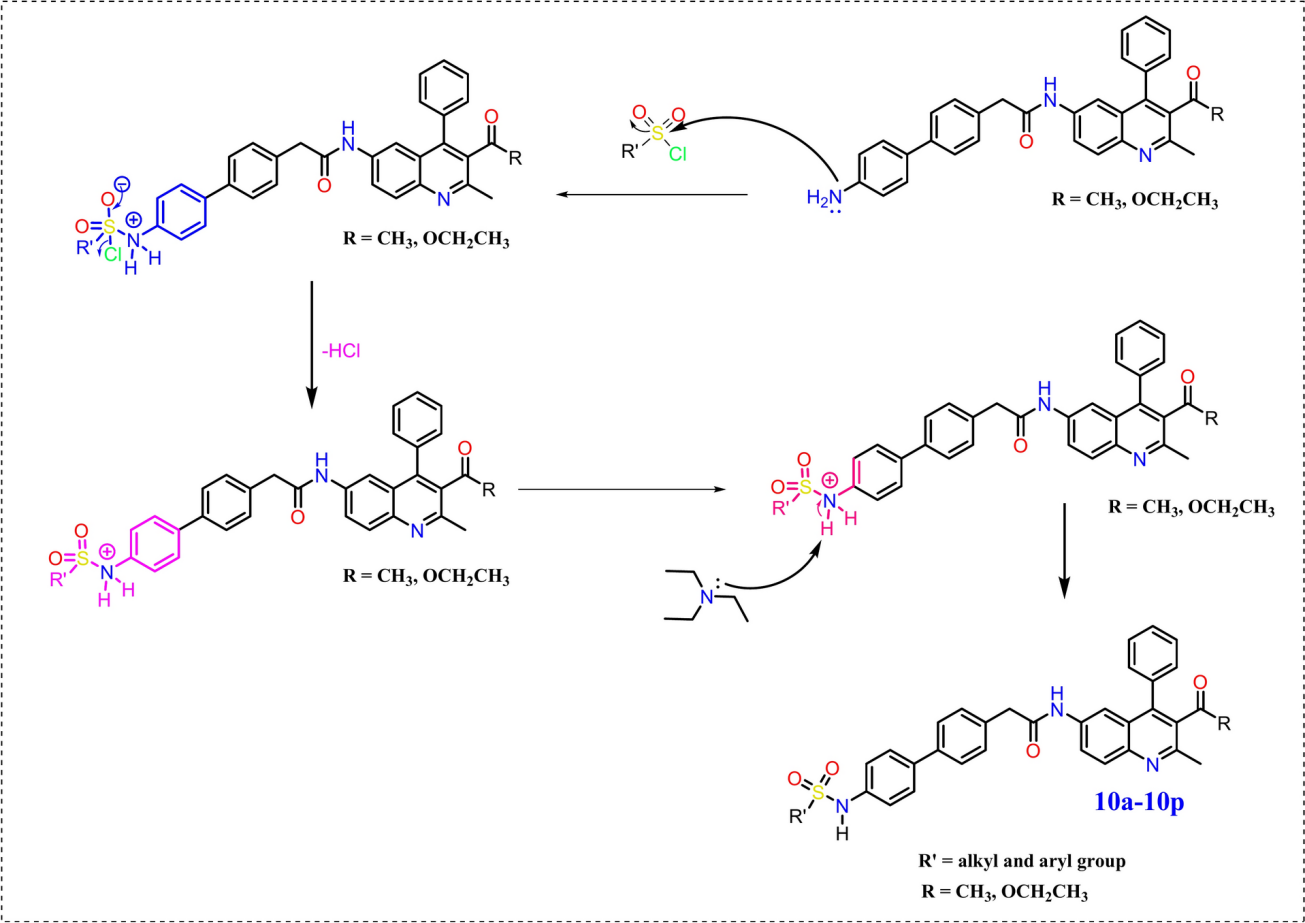
General reaction mechanism of synthesized derivatives (**10a**-**10p**).

The synthesis of the compound designated in the title demands the execution of a sequence comprising five distinct steps, as outlined in Scheme 1. Initially, the requisite intermediates, namely 1-(2-methyl-6-nitro-4-phenylquinolin-3-yl)ethan-1-one and ethyl 2-methyl-6-nitro-4-phenylquinoline-3-carboxylate, were obtained through the cyclization of 2-amino-5-nitroaminobenzophenone (**1**) with pentan-2,4-dione and ethyl 3-oxobutanoate under acidic conditions^36^. Subsequently, the nitro functional group underwent reduction to an amine utilizing zinc dust in conjunction with ammonium chloride, resulting in the formation of 1-(6-amino-2-methyl-4-phenylquinolin-3-yl)ethan-1-one and ethyl 6-amino-2-methyl-4-phenylquinolin-3-carboxylate, which were thereafter subjected to an acid–amine cross-coupling reaction employing 2-(4-bromophenyl)acetic acid and HATU as the coupling reagent. This was followed by the synthesis of precursor (**8**) through a Suzuki cross-coupling reaction facilitated by a palladium catalyst and K_2_CO_3_ as the base,^37^ concluding in the formation of the title compound via alkylation involving various substituted sulfonyl chlorides and DIPEA (N-ethyl-N-isopropylpropan-2-amine), yielding compounds (**10a**-**10p**) as depicted in Scheme 1. The synthesized quinoline-sulphonamide derivatives, illustrated in Figs. 2, 3 shows general reaction mechanism of all synthesized derivatives, synthesized derivatives were characterized utilizing an array of spectroscopic methodologies, including ^1^H, ^13^C, and ^19^F NMR spectroscopy, alongside 2D NMR and DEPT-135 spectra, as well as high-resolution mass spectrometry (HRMS). In the ^1^H NMR spectra, the NH resonance is detected within the frequency range of 10.818–9.833 ppm, while aromatic signals are identified within the pertinent chemical shift range of δ 8.113–7.190 ppm, and the methyl proton at the second position is observed in the interval of 2.62–2.61 ppm. The proton at the third position of the quinoline carbonyl ketone resonates in the vicinity of 2.00–1.99 ppm, and the ester functionality displays resonances at δ 4.02–3.97 (q) ppm and 0.89–0.96 (t) ppm. In **10e** compound ^19^F peak appeared at − 61.71 ppm. The ^13^C NMR spectra exhibited chemical shifts that align with the anticipated compounds, wherein the CH_2_ peaks in the DEPT-135 spectra presented negative signals, while the CH_3_ and CH peaks demonstrated positive signals. Furthermore, the HRMS spectra revealed peaks that correspond to [M + 1], thereby substantiating the identity of the synthesized compounds.

### UV–vis and fluorescence

**Fig. 4:**
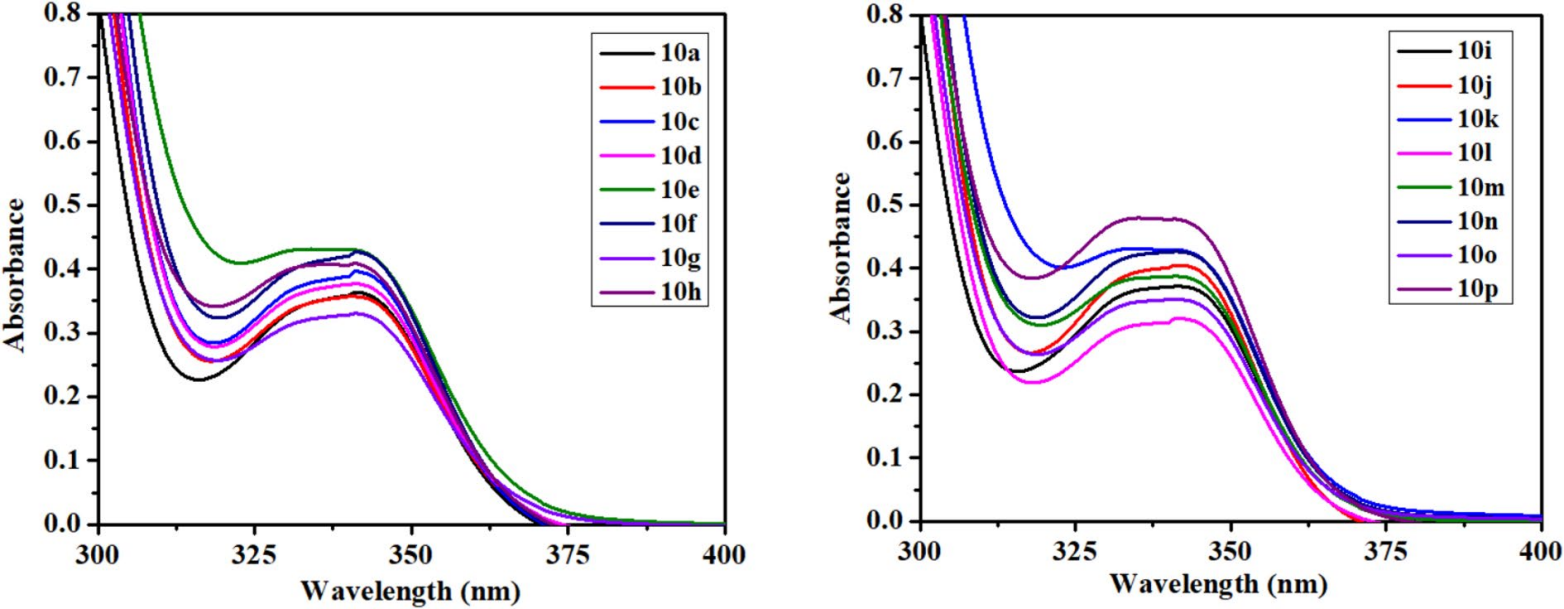
UV–vis spectrum of compounds in acetonitrile solution.

The photophysical properties of molecules are essential for understanding their interaction with light, particularly how they absorb and emit photons. Absorbance, a critical photophysical parameter, quantifies a substance’s ability to absorb light at specific wavelengths, directly correlating with the electronic transitions within the molecule^38^. This behavior is governed by the Beer-Lambert law, which elucidates the linear relationship between absorbance and both the concentration of the absorbing species and the path length of the light through the material. By analyzing the absorbance spectrum, one can precisely identify the absorption peaks, which correspond to distinct electronic transitions such as π → π* or n → π*. These peaks provide insights into the electronic structure, molecular orbitals, and the influence of the surrounding environment on the photophysical behavior of the substance^39^. The UV–Vis absorption spectra (Fig. 4) of all synthesized compounds were recorded in acetonitrile at a concentration of 2 × 10^−5^ M, revealing absorption bands within the range of 337.83 nm to 341.73 nm. These bands are indicative of electronic transitions, specifically n → π* transitions, which are characteristic of the chromophoric systems in the compounds. Notably, the observed spectral shifts both red and blue shifts suggest variations in the electronic environment and the extent of conjugation within the molecules. The emission behavior is highly dependent on the nature of the electronic transitions and the surrounding solvent environment^40^.

**Fig. 5:**
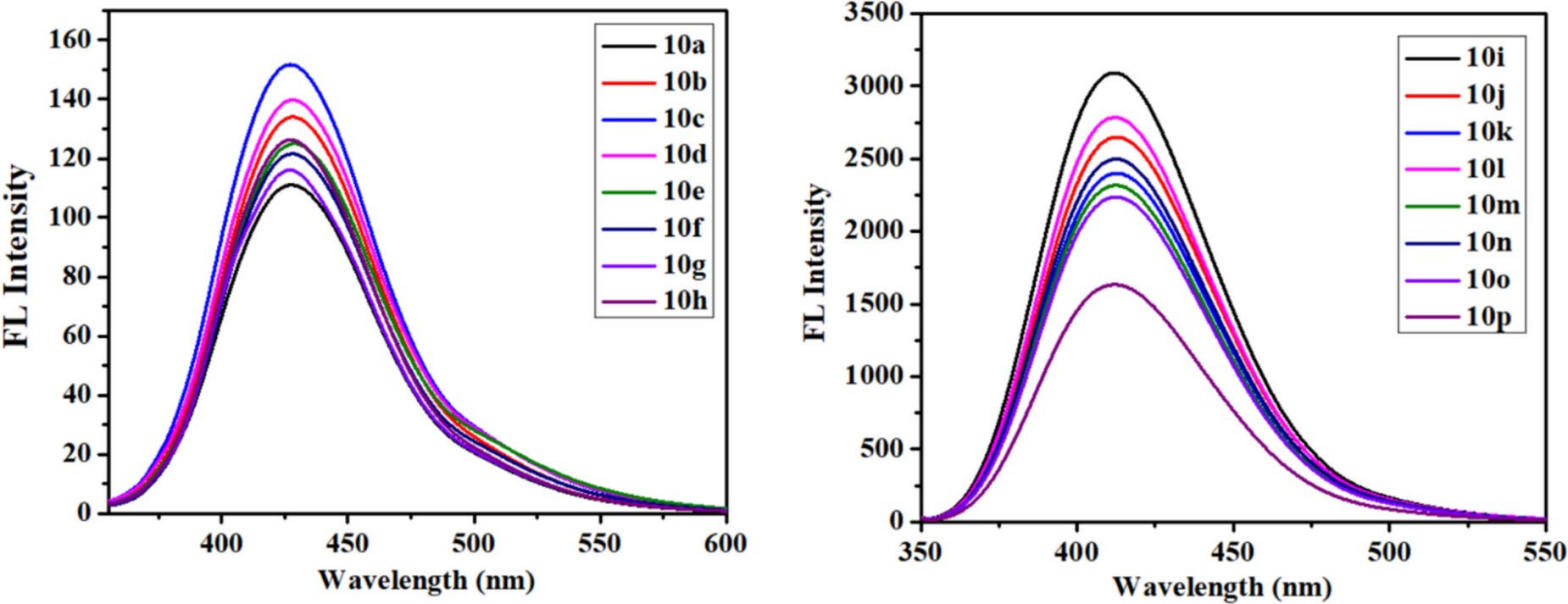
FL spectrum of compounds in acetonitrile solution.

The fluorescence (FL) spectra (Fig. 5) of all synthesized compounds were recorded in acetonitrile at a concentration of 2 × 10^−5^ M, with an excitation wavelength of 340 nm. The emission wavelengths ranged from 411.70 nm to 429.90 nm, indicating a redshift as the emitted photons have longer wavelengths and lower energy compared to the absorbed photons. This redshift is a key feature of fluorescence, where the energy dissipation between absorption and emission is quantified by the Stokes shift. The specific redshift observed between the emission peaks of 411.70 nm to 429.90 nm highlights the relaxation of the excited state, leading to longer wavelength emissions^41^. The lower fluorescence intensity of a ketone group compared to a carboxylic ester at the 3rd position of quinoline is mainly due to their differing electronic effects on the quinoline ring. A ketone is a strong electron-withdrawing group, which can destabilize the excited state and increase non-radiative decay pathways like intersystem crossing, thus reducing fluorescence intensity. On the other hand, a carboxylic ester, while still slightly electron-withdrawing, has a resonance contribution that can stabilize the excited state better, resulting in a higher probability of radiative decay (fluorescence). Additionally, the ester group can better stabilize the LUMO, enhancing fluorescence intensity compared to the ketone.

The Stokes shift minimizes self-quenching, improves signal-to-noise ratio, enables multiplexing, and provides insight into molecular environments by separating excitation and emission wavelengths. In compounds **10h** and **10e** are observed with large stokes shift. The smallest Stokes shift, measured at 0.5053 × 10^4^, was observed for **10j**, while the largest, recorded at 0.6237 × 10^4^, was found for **10h**, in measurements conducted in acetonitrile. The stokes shift holds significance for fluorescence method sensitivity, facilitating efficient photon detection amidst low background and separation from the excitation source. Using Beer–Lambert’s rule A = εcl, the molar extinction coefficient (ε) was computed. As the absorbance per unit concentration and path length, the molar extinction coefficient is determined. The mathematical expression for it is ε = A/(c × l), where l is the light’s path length through the sample, c is the absorbing species’ concentration, and A is its absorbance. The value of epsilon exhibited a range of variance from 1.7500 × 10^4^ M^−1^ cm^−1^ to 2.4000 × 10^4^ M^−1^ cm^−1^ as illustrated in Table 1. In the case of quinoline derivatives, compound **10o** is characterized by the presence of a chloro-sulphonamide group, while compound **10p** with a nitro aryl moiety attached to it, leading to the indication of the highest molar extinction coefficient among the compounds analyzed. The quantum efficiencies were evaluated by comparing them to the well-established quantum efficiencies of quinine sulfate in a solution of 0.5 M H_2_SO_4_ (ɸ = 0.54) under an excitation wavelength of 340 nm. It was observed that the introduction of Ar-CH_2_ ring substituents with third position carbonyl ester group and sulphonamide group significantly enhanced the quantum yield; conversely, the presence of 3rd position Carboxylic ketone group was found to have a suppressive impact on the quantum yield, with a quantum yield range of 0.015 to 0.558, as indicated in Table 1.

**Table 1:** Photophysical properties data of synthesized compounds (**10a**-**10p**).

Code	λ_Abs_	λ_Emi_	Molar extinction coefficient (ɛ) (ɛ × 10^−4^) M^−1^ cm^−1^	Stokes shift (Δ × 10^−4^)	Quantum yield (ɸ)
**10a**	339.63	427.63	1.7500	0.6059	0.015
**10b**	340.00	428.90	1.7540	0.6096	0.018
**10c**	341.21	428.02	1.9500	0.5944	0.0184
**10d**	340.04	429.9	1.8500	0.6147	0.0182
**10e**	338.41	428.26	2.1500	0.6199	0.0159
**10f**	341.55	429.9	2.1000	0.5962	0.0171
**10g**	340.39	429.69	1.6000	0.6105	0.0171
**10h**	337.83	428.02	2.0500	0.6237	0.0143
**10i**	340.22	411.70	1.8000	0.5103	0.3197
**10j**	341.73	413.06	1.9500	0.5053	0.558
**10k**	338.99	412.50	2.1500	0.5256	0.2104
**10l**	340.80	413.3	1.5500	0.5147	0.0232
**10m**	340.22	413.26	1.9000	0.5194	0.2283
**10n**	340.22	412.26	2.1000	0.5136	0.2228
**10o**	340.33	413.53	1.7500	0.5201	0.24
**10p**	338.41	412.50	2.4000	0.5307	0.1267

## Computational studies

### Density functional theory

Density functional theory (DFT) stands as a preeminent theoretical framework with broad applicability in scientific inquiry. Its utility spans diverse domains, encompassing the elucidation of compound kinetic and thermodynamic stability, structural determinations, mechanistic elucidations, molecular interactions analysis, and the exploration of atoms and molecules’ optical and electronic attributes. In this investigation, Gaussian 16 software^42^ served as the computational workhorse, executing all theoretical computations. The compound’s molecular geometry underwent optimization via DFT, employing the B3LYP/6-31G’(d,p) foundational level set. Subsequently, GaussView 6.0^43^ emerged as an indispensable tool for the visualization of intricate molecular structures, thereby facilitating a comprehensive understanding of the studied systems.

#### Frontier molecular orbitals (FMOs)

The process of geometry optimization employs the B3LYP functional in conjunction with the 6-31G’ (d,p) basis set, which is esteemed for its precision and computational efficacy. Crucial parameters pertaining to the electronic structure, including the Highest Occupied Molecular Orbital (HOMO), the Lowest Unoccupied Molecular Orbital (LUMO), along with their respective energies (E_LUMO_ and E_HOMO_), were systematically calculated. The energy gap (E_gap_) between the HOMO and LUMO was determined through the application of Koopman’s theorem^44^. This helps in the achievement of different research goals. DFT-based research ultimately allowed for a thorough understanding of these compounds’ electronic characteristics and interactions, which made it easier to investigate the compounds’ possible uses in a variety of sectors^45–52^.

The space-related arrangement and distribution of electron charge within the HOMO and the LUMO of the quinoline-sulphonamide derivatives are meticulously illustrated throughout the molecular structure, as exemplified in the detailed figures presented in the supplementary information, specifically ranging from Figures S3.1 to S3.16. Such intricate characteristics not only offer profound insights into the intricate behaviour of the molecule but also significantly enhance our comprehension of its chemical reactivity, thereby contributing to the broader field of molecular chemistry. The compounds possessing the highest values of the HOMO are capable of functioning effectively as electron donors, thereby facilitating electron transfer processes in various chemical reactions. Notably, the specific compounds designated as **10a**, **10b**, and **10l** are distinguished by exhibiting the most elevated HOMO values, which are quantified with respective energy levels of − 5.9243 eV, − 5.8984 eV, and − 5.8892 eV, indicating their potential reactivity and stability in electronic interactions. Conversely, it is important to note that the molecules characterized by possessing the lowest values of the LUMO have the capability of acting as electron acceptors, and in this context, the particular compounds identified as **10k**, **10m**, and **10j** are highlighted for their notably low LUMO energies, which are measured at − 2.0156 eV, − 2.0156 eV, and − 2.0166 eV, respectively, thus revealing their significant role in the acceptance of electrons in relevant chemical processes.

**Fig. 6:**
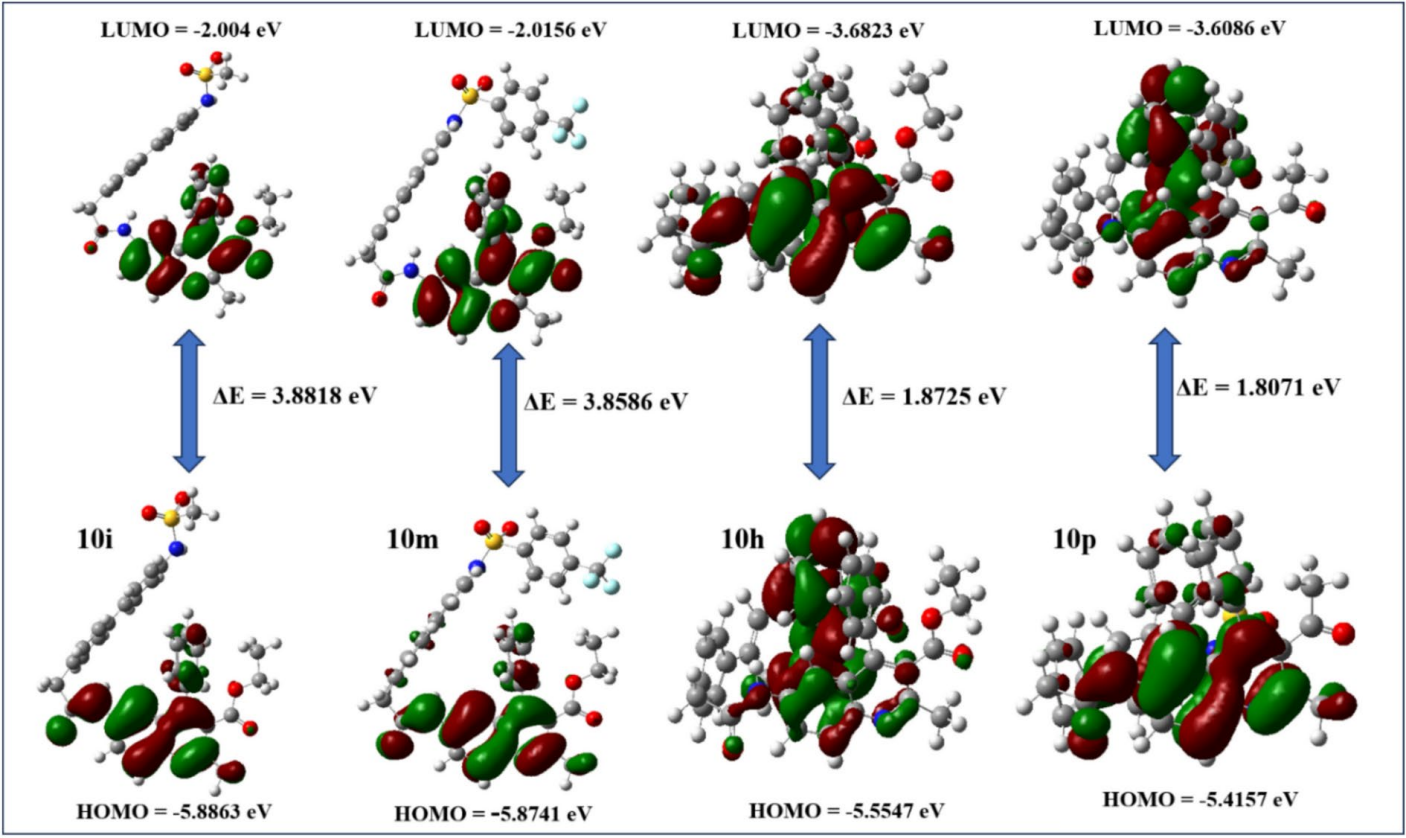
Frontier molecular orbital diagram of compounds **10i**, **10m**, **10h**, and **10p**.

The molecules distinguished by minimal energy gaps exhibit significantly enhanced reactivity in comparison to their counterparts characterized by substantial energy gaps, a phenomenon that can be attributed to the diminished excitation energy that is required to surmount the energy barrier that separates the ground state from the excited state. The term excitation energy is defined as the amount of energy that is necessary for the promotion of an electron from its foundational ground state to a higher, excited state, and this parameter is crucial in the comprehensive understanding of various electronic transitions, the intricate details of optical absorption spectra, as well as the complex behaviours of electrons within molecules and materials, all of which can be effectively analyzed within the theoretical framework provided by DFT computations. Molecules that possess smaller energy gaps inherently require a reduced amount of excitation energy, which subsequently makes them significantly more susceptible to engaging in various chemical reactions, as they can more easily overcome the requisite energy barriers and thus actively participate in a wide array of diverse chemical processes. Among the various Quinoline-Sulphonamide compounds that were accurately examined in the context of this study (Table 2), the compounds designated as **10i**, **10m**, **10n**, and **10o** distinctly demonstrate a comparatively broader energy band gap, which is quantitatively represented by their respective energy gap values of 3.8818 eV, 3.8586 eV, 3.8502 eV, and 3.8534 eV (Fig. 6); this observation underscores the significant electronic properties that these compounds possess. Conversely, in stark contrast to the compounds, the compounds identified as **10h** and **10p** reveal the lowest energy band gaps among those evaluated, with their measurements recorded at 1.8725 eV and 1.8071 eV, respectively (Fig. 6), which emphasizes their relatively narrower electronic structures. The assessment of a molecule’s inherent chemical hardness is greatly enhanced by examining the energy gap between the LUMO and the HOMO, as this gap serves as a dependable indicator of the molecule’s reactivity profile within various chemical contexts. Specifically, a smaller value of this energy gap corresponds to an elevated level of molecular softness, suggesting a propensity for greater reactivity, whereas a larger LUMO–HOMO energy gap is indicative of an increased degree of molecular hardness, signifying a reduced reactivity potential in comparison. Figure 6 shows the electron distribution of **10i**, **10m**, **10h**, and **10p** under HOMO–LUMO. Green is negative (electrophile), while red is positive (nucleophile).

**Table 2:** The compound’s global reactivity descriptors and FMO energy parameters.

Code	HOMO	LUMO	ΔE	*I*	A	χ	μ	Η	S	ω
**10a**	− 5.9243	− 2.1605	3.7638	5.9243	2.1605	4.0424	− 4.0424	1.8819	0.5314	4.3417
**10b**	− 5.8984	− 2.1638	3.7346	5.8984	2.1638	4.0311	− 4.0311	1.8673	0.5355	4.3511
**10c**	− 5.7478	− 2.2872	3.4606	5.7478	2.2872	4.0175	− 4.0175	1.7303	0.5779	4.664
**10d**	− 5.7264	− 2.1738	3.5527	5.7264	2.1738	3.9501	− 3.9501	1.7763	0.563	4.392
**10e**	− 5.8874	− 2.838	3.0494	5.8874	2.838	4.3627	− 4.3627	1.5247	0.6559	6.2415
**10f**	− 5.8528	− 2.4932	3.3596	5.8528	2.4932	4.173	− 4.173	1.6798	0.5953	5.1833
**10g**	− 5.8649	− 2.5013	3.3637	5.8649	2.5013	4.1831	− 4.1831	1.6818	0.5946	5.2022
**10h**	− 5.5547	− 3.6823	**1.8725**	5.5547	3.6823	4.6185	− 4.6185	0.9362	1.0681	11.392
**10i**	− 5.8863	− 2.0045	**3.8818**	5.8863	2.0045	3.9454	− 3.9454	1.9409	0.5152	4.01
**10j**	− 5.8604	− 2.0166	3.8437	5.8604	2.0166	3.9385	− 3.9385	1.9219	0.5203	4.0356
**10k**	− 5.8528	− 2.0156	3.8372	5.8528	2.0156	3.9342	− 3.9342	1.9186	0.5212	4.0335
**10l**	− 5.8892	− 2.0663	3.8229	5.8892	2.0663	3.9778	− 3.9778	1.9115	0.5232	4.1389
**10m**	− 5.8741	− 2.0156	**3.8586**	5.8741	2.0156	3.9448	− 3.9448	1.9293	0.5183	4.033
**10n**	− 5.8693	− 2.0191	**3.8502**	5.8693	2.0191	3.9442	− 3.9442	1.9251	0.5195	4.0404
**10o**	− 5.8712	− 2.0177	**3.8534**	5.8712	2.0177	3.9444	− 3.9444	1.9267	0.519	4.0376
**10p**	− 5.4157	− 3.6086	**1.8071**	5.4157	3.6086	4.5121	− 4.5121	0.9036	1.1067	11.266

Significant values are given in bold.

#### Molecular electrostatic potential (MEP)

**Fig. 7:**
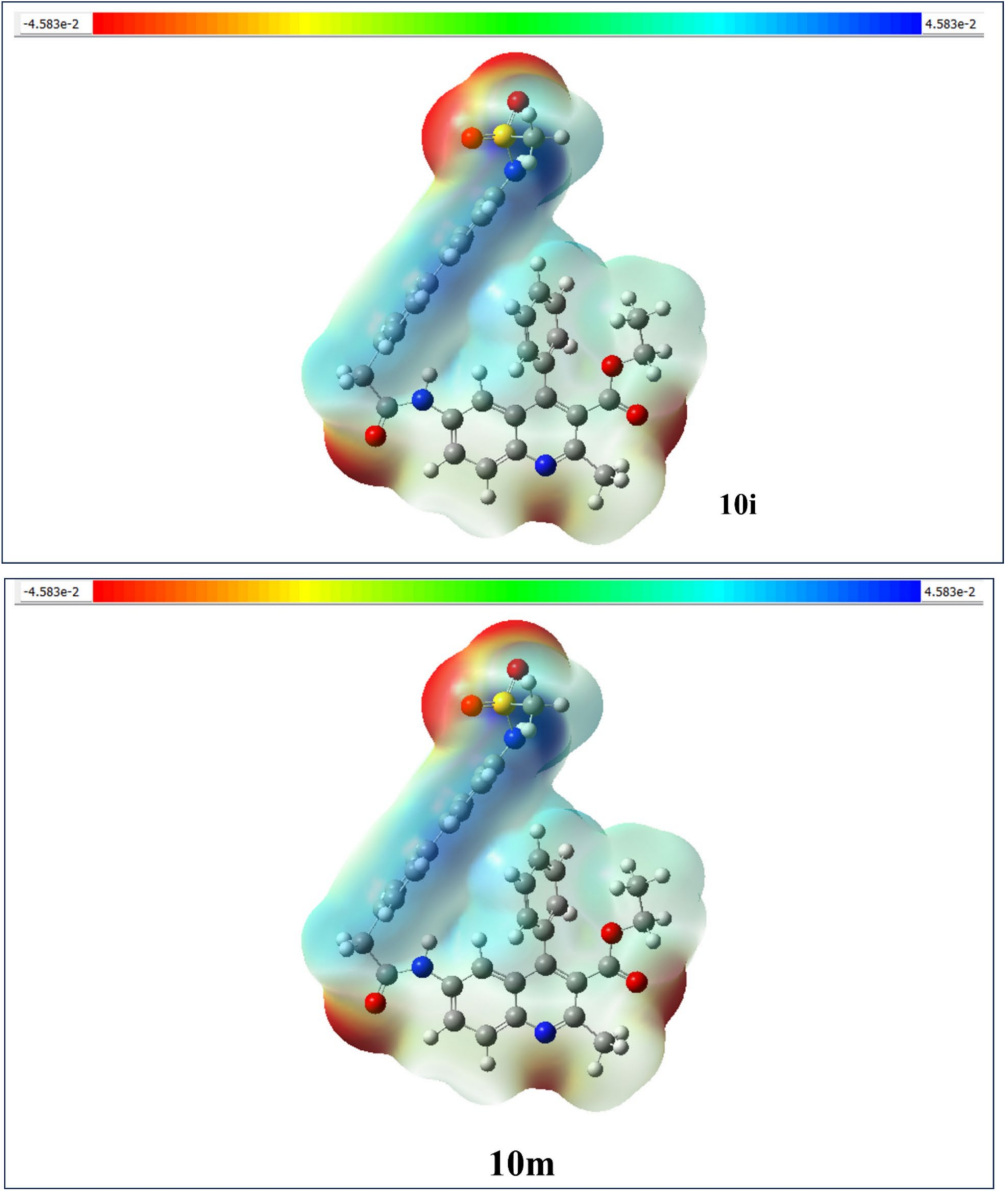

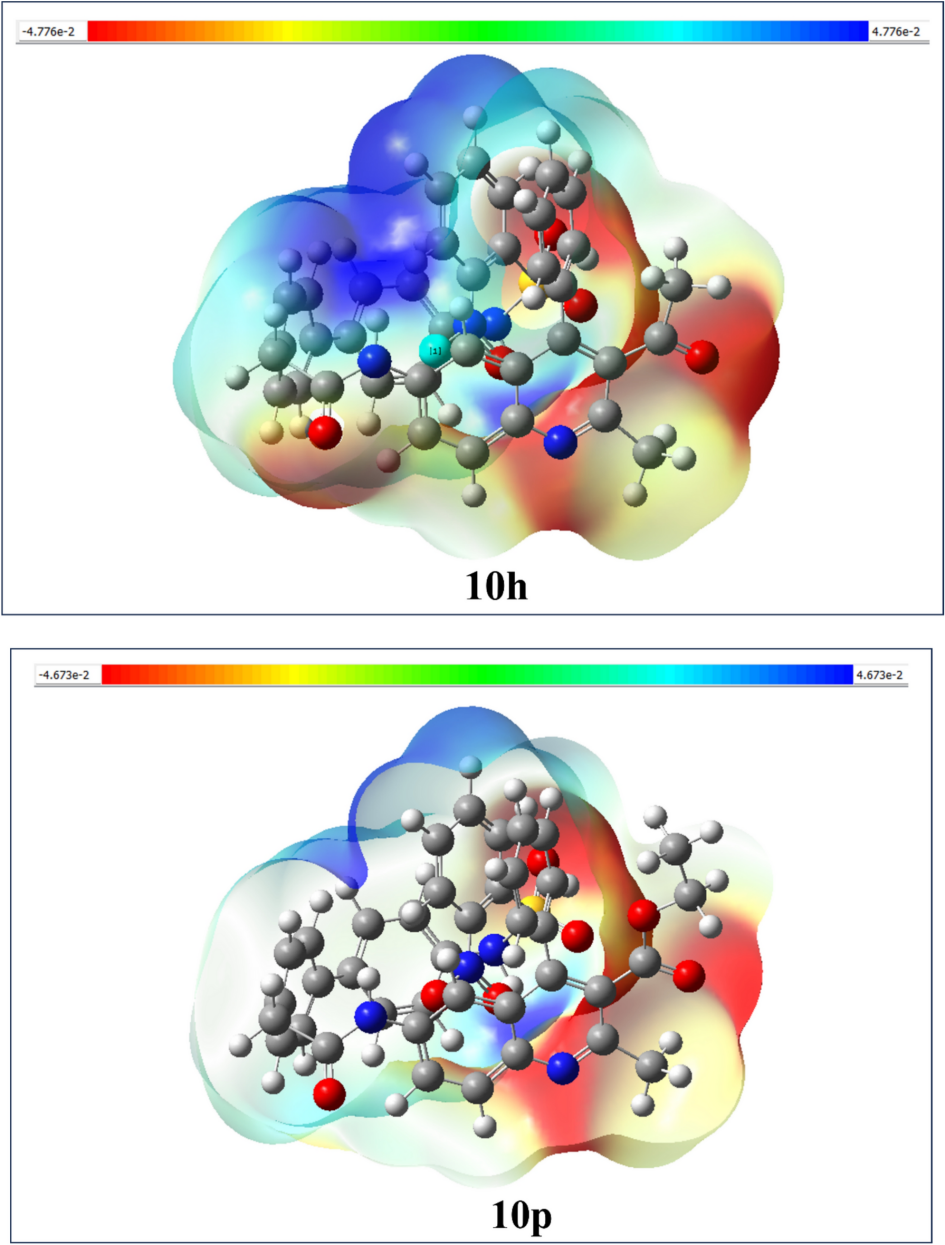
Molecular electrostatic potential map of the investigated compound of **10i**, **10m**, **10h** and **10p**.

The molecular electrostatic potential (MEP) of the various synthesized compounds has been carefully calculated using the electron density profiles inherent to their molecular structures. This detailed analysis is visually represented in Fig. 7, employing a sophisticated color-coded scale that illustrates the electrostatic characteristics. The MEP diagram provides a comprehensive overview of charge distribution within the molecular framework, allowing for the prediction of potential electrophilic and nucleophilic interaction sites throughout the complex. This prediction is grounded in the analysis of electron density^53^. All synthesized compounds that underwent MEP assessments are displayed in the supplementary information, specifically in Figure S4, which offers additional context. The MEP analysis reveals the distinct electron distribution patterns and density profiles associated with compounds **10i**, **10m**, **10h**, and **10p**. These patterns are effectively shown in Fig. 7, which acts as a map of the electrostatic potential surface of the compounds. In the electrostatic potential scale, the color gradient demonstrates an increase in electrostatic potential, with red indicating the highest potential values and transitioning to blue at the lower end of the spectrum. Green regions within the diagram represent areas with no electrostatic potential, providing a clear visual cue for zones devoid of charge interaction. Specifically, electrophilic sites are marked in red, nucleophilic sites in blue, and neutral electrostatic potential regions are clearly indicated in green. In the MEP map, the oxygen atoms within the molecular structure are highlighted as strong electrophilic regions, characterized by a vivid red-to-orange color spectrum. This visually underscores their significant role in the compound’s electrophilic reactivity. Conversely, regions exhibiting blue coloration indicate positive electron density, aligning them with characteristics associated with nucleophilic reactivity. Thus, the MEP analysis showcases the complexity of molecular interactions at play.

#### Time-dependent density functional theory (TD-DFT)

**Fig. 8:**
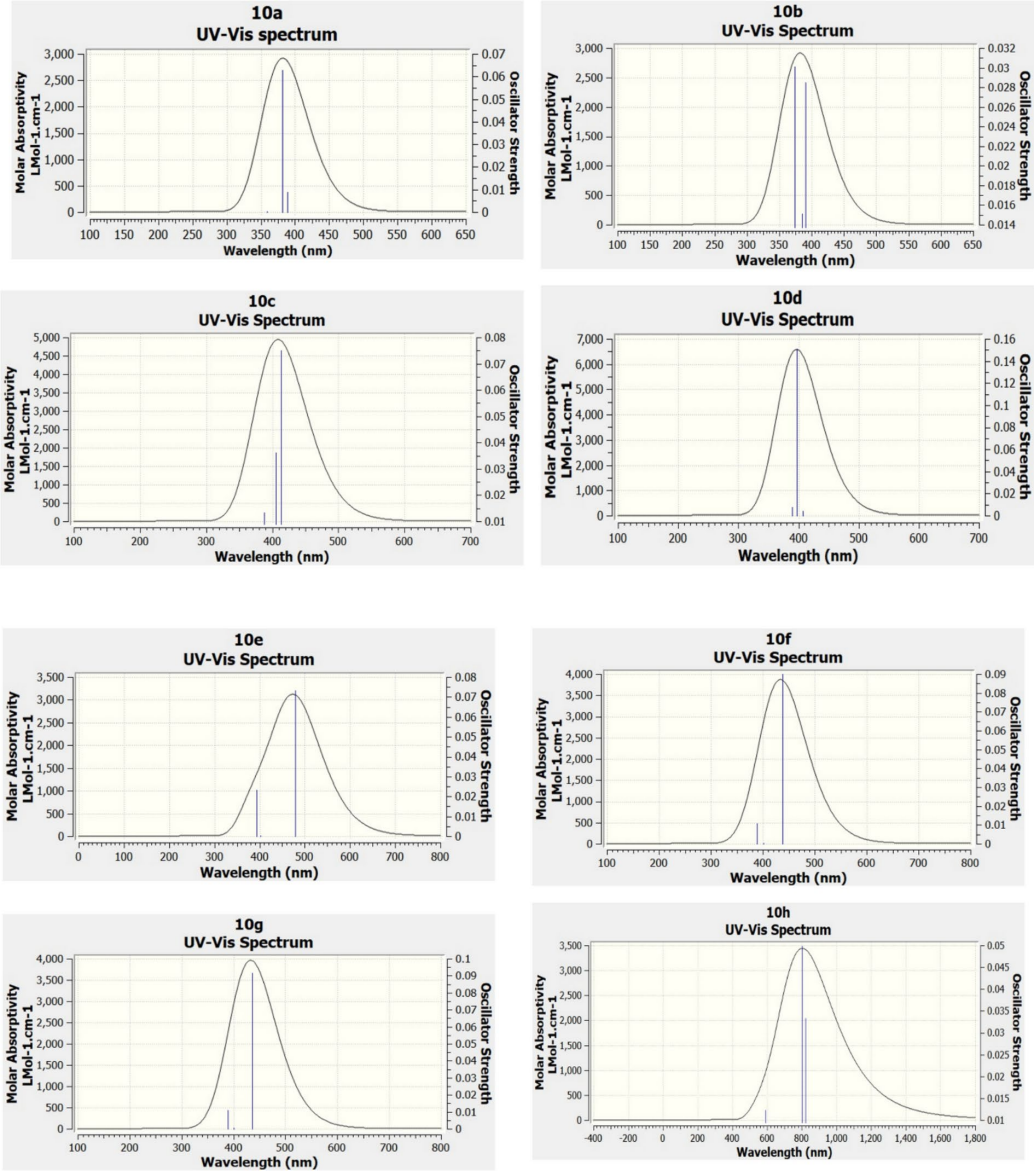
Theoretical UV–Vis spectra of compounds were calculated using TD-DFT with the B3LYP/6-31G’(d,p) methodology. The blue lines illustrate the principal electronic transitions in the singlet state.

Density functional theory (DFT) furnishes a foundational quantum mechanical paradigm, positing that the attributes of a system are contingent upon charge density functions. This theoretical framework facilitates precise computations that illuminate the structural, energetic, and molecular features of various compounds. Building upon the principles of DFT to accommodate excited states, time-dependent density functional theory (TD-DFT) emerges, which permits the calculation of electronic spectra associated with absorption phenomena^54,55^. TD-DFT enables investigations into a multitude of processes engaged with excited states, yielding insights into phenomena such as molecular transitions and optical properties. The interplay between DFT and TD-DFT methodologies provides a formidable toolkit for an exhaustive analysis of molecular dynamics, thereby enhancing our understanding in the realms of chemical reactivity, spectroscopy, and materials science. By utilizing the B3LYP functional alongside 6-31G’ (d,p) basis sets, UV–vis spectra were forecasted through TD-DFT, yielding comprehensive theoretical absorption profiles that are critical for elucidating molecular absorptivity. GaussView 6 was utilized to extract and visualize relevant UV–vis spectral data, as depicted in Fig. 8 and in additional spectra presented in the supplementary information Figure S5. The juxtaposition with experimental data in Table 3 underscores the dependability of TD-DFT predictions, illustrating its efficiency in simulating molecular absorption phenomena and augmenting the understanding of molecular characteristics^56–58^.

**Table 3:** Selected major contribution transition states of compound **10a**-**10e** and **10i**-**10m**.

Entry	Experimental λ_Abs_	Theoretical λ_Abs_	Oscillation strength, f	Energy, ΔE	Selected major contributions (%)
**10a**	339.63	388.53	0.0090	3.1911	H + 2 → L (52.99)
		381.27	0.0628	3.2518	H → L (78.96)
		358.96	0.0004	3.4540	H + 1 → L (88.86)
**10b**	340.00	390.09	0.0285	3.1784	H → L (46.68)
		385.61	0.0151	3.2153	H → L (35.09)
		373.69	0.0301	3.3179	H → L + 1 77.96
**10c**	341.21	413.58	0.0750	2.9979	H → L-1 (52.53)
		405.69	0.0362	3.0561	H → L (53.56)
		388.21	0.0133	3.1937	H + 2 → L (48.39)
**10d**	340.04	406.83	0.0042	3.0475	H → L (99.12)
		397.25	0.1508	3.1211	H → L-1 (97.56)
		388.97	0.0074	3.1875	H + 2 → L (56.73)
**10e**	338.41	478.79	0.0734	2.5895	H → L (97.77)
		401.98	0.0004	3.0843	H → L-1 (98.42)
		393.12	0.0231	3.1538	H + 1 → L (83.83)
**10i**	340.22	368.13	0.0763	3.3680	H → L (94.54)
		347.69	0.0005	3.5659	H + 1 → L (94.28)
		343.63	0.0017	3.6080	H + 2 → L (95.69)
**10j**	341.73	374.42	0.0459	3.3113	H → L (81.21)
		360.43	0.0337	3.4399	H + 1 → L (79.91)
		344.17	0.0019	3.6025	H + 2 → L (98.15)
**10k**	338.99	375.26	0.0417	3.3040	H → L (80.29)
		362.28	0.0393	3.4223	H + 1 → L (78.84)
		344.43	0.0018	3.5997	H + 2 → L (98.31)
**10l**	340.80	375.90	0.0436	3.2983	H → L (80.29)
		360.69	0.0358	3.4374	H + 1 → L (78.84)
		344.19	0.0020	3.6022	H + 2 → L (98.31)
**10m**	340.22	374.17	0.0517	3.3136	H → H\L (79.25)
		359.44	0.0291	3.4494	H + 1 → L (78.40)
		344.65	0.0017	3.5974	H + 2 → L (98.27)

The discrepancies observed between the spectra obtained through simulation and those acquired via experimental methods can be attributed to variations in both oscillator strength and energy levels. The comprehensive spectral analysis undertaken in this study has unveiled the presence of unique medium energy band n-π* transitions across a diverse array of compounds: specifically, compound **10a** showcased notable absorption peaks at wavelengths of 388.53 nm, 381.27 nm, and 358.96 nm; similarly, compound **10b** exhibited transitions occurring at 390.09 nm, 385.61 nm, and 373.69 nm; furthermore, for compound **10d**, absorption bands were identified at 406.83 nm, 397.25 nm, and 388.97 nm; compound **10i**, on the other hand, displayed absorption peaks at 368.13 nm, 347.69 nm, and 343.63 nm; additionally, compound **10j** presented absorption peaks at 374.42 nm, 360.43 nm, and 344.17 nm; while compound **10k** demonstrated electronic transitions at 375.26 nm, 362.28 nm, and 344.19 nm; these significant findings serve to elucidate the intricate electronic transitions that occur within the various compounds under investigation.

Additionally, the compounds designated as **10d**, **10e**, **10i**, **10k**, and **10l** have been observed to exhibit remarkably high contributions exceeding the threshold of 50% within their respective spectral representations, which serves as a strong indicator of their substantial involvement in the electronic transitions that have been documented and analyzed throughout this study. All of the compounds that were synthesized and subsequently analyzed were subjected to calculations of three excited state transition states, which were derived from the selected major electronic transition contributions that have been meticulously detailed in Table S1. The electronic transitions have been evaluated at a foundational level using the B3LYP functional in conjunction with the 6–31 + G’(d,p) basis set, employing an iso surface value set at 0.02 atomic units (au); within this framework, the transitions can be categorized into lower energy (LE) and higher energy (HE) bands, specifically the transitions from the ground state S0 to the first excited state S1, and from the ground state S0 to the second excited state S2, both of which are indicative of the intramolecular charge transfer (ICT) process occurring within the studied compounds.

The pertinent and relevant major distribution for the molecular entity referred to as Compound **10d** is characterized by the transitions from the HOMO to the LUMO at an impressive percentage of 99.12%, followed closely by the transition from HOMO to LUMO-1 at a notable 97.56%, and additionally, the transition from the HOMO + 2 state to the LUMO state at a significant percentage of 56.73%; in comparison, for Compound **10e**, the transition from HOMO to LUMO occurs at a remarkable 97.77%, the transition from HOMO-2 to LUMO at a substantial 85%, and furthermore, the transition from HOMO to LUMO is recorded at 80%. When examining Compound **10i**, it is observed that the transition from HOMO to LUMO is at a considerable 94.54%, with the transition from HOMO + 1 to LUMO closely following at 94.28%, while the transition from HOMO + 2 to LUMO exhibits an impressive 95.69%. Transitioning to Compound **10k**, the distribution reveals that the transition from HOMO to LUMO is at a noteworthy 80.29%, the transition from HOMO + 1 to LUMO at 78.84%, and the transition from HOMO + 2 to LUMO reaches a remarkable 98.31%. Similarly, for Compound **10l**, the transition from HOMO to LUMO is once again recorded at 80.29%, while the transition from HOMO + 1 to LUMO is at 78.84%, and the transition from HOMO + 2 to LUMO is registered at an outstanding 98.31%; these transitions collectively elucidate the donor-π-acceptor charge transfer character inherent within these specific compounds.

## Molecular docking study

The molecular modeling studies followed a well-established protocol using various software tools, including ChemDraw Professional 16.0^59^, AutoDock4.0^60,61^, Discovery Studio Visualizer v20^62,63^ and PyMOL^64^. Molecular docking analysis employed a grid with its center positioned at X = 41.09, Y = 8.686, and Z = 2.123 for dihydropteroate synthase (DHPS), and X = 63.361, Y = 71.934, and Z = 35.631 for DNA Gyrase (gyrA). The grid box was configured with dimensions of 60 Å × 60 Å × 60 Å, and a spacing of 0.375 Å between the grid points was utilized. Rigid molecular docking was conducted to evaluate the most promising compound (**10p**) against both DHPS and GyrA targets, considering their respective mechanistic differences.

This investigation employed *Escherichia coli* (*E. coli*) to assess the biological efficacy of **10p**. *E. coli*, particularly uropathogenic strains, represent the principal causative agents of urinary tract infections and rank among the most isolated pathogens in instances of neonatal meningitis and hospital-acquired bacteremia^65,66^. The newly synthesized quinoline derivatives possess both a quinoline moiety and sulfur groups in their structures. To evaluate their activity, DHPS and gyrA were considered the two major targets as two targets countered by two different classes of antibiotics sulfanamides and quinolones. The PDB IDs of these target proteins are **1AJ0** and **1AB4**, respectively.

Sulfonamide compounds primarily target DHPS, an enzyme of paramount importance in the folic acid biosynthetic pathway. These pharmaceutical agents function as competitive inhibitors of DHPS, effectively obstructing bacterial production of folic acid, a molecule vital for microbial survival. The structural similarity between sulfonamides and para-aminobenzoic acid (PABA), an essential precursor in folic acid synthesis, enables these drugs to occupy the DHPS-binding site, thereby preventing PABA attachment and subsequent enzymatic reactions. Since folic acid plays a crucial role in bacterial DNA synthesis and cellular division, interruption of its production significantly impairs microbial growth and proliferation. In this study, sulfamethoxazole, a sulfonamide antibiotic used to treat bacterial infections, was used as a control drug. It is frequently administered in combination with trimethoprim to treat various infections, including those of the urinary tract^67–69^.

**Fig. 9:**
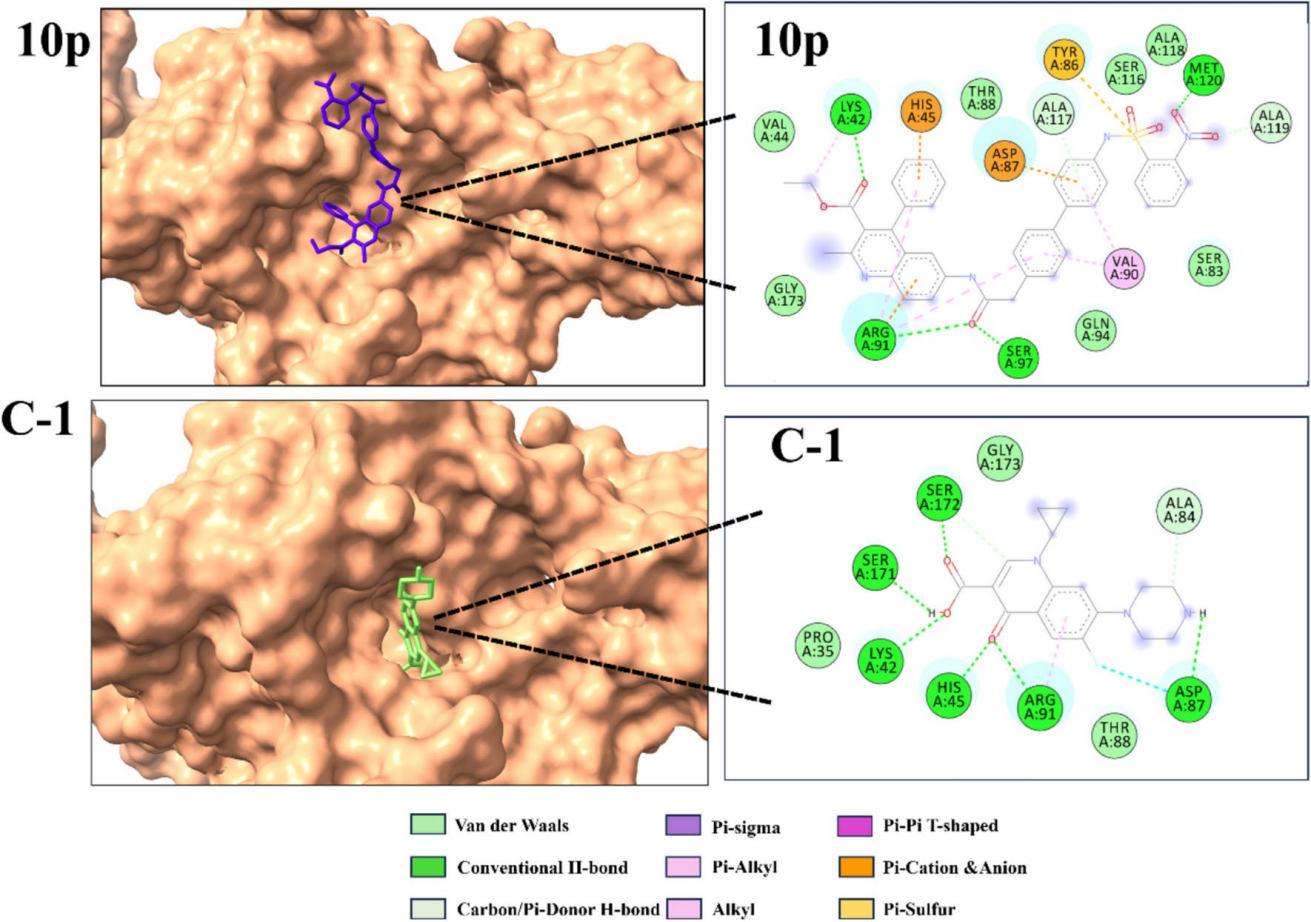
Depicted 2D and 3D interaction of compound **10p** and studied crystal structures by docking simulations C-1 = Ciprofloxacin (Control) against DNA gyrase protein (PDB :1AJ0).

**Fig. 10:**
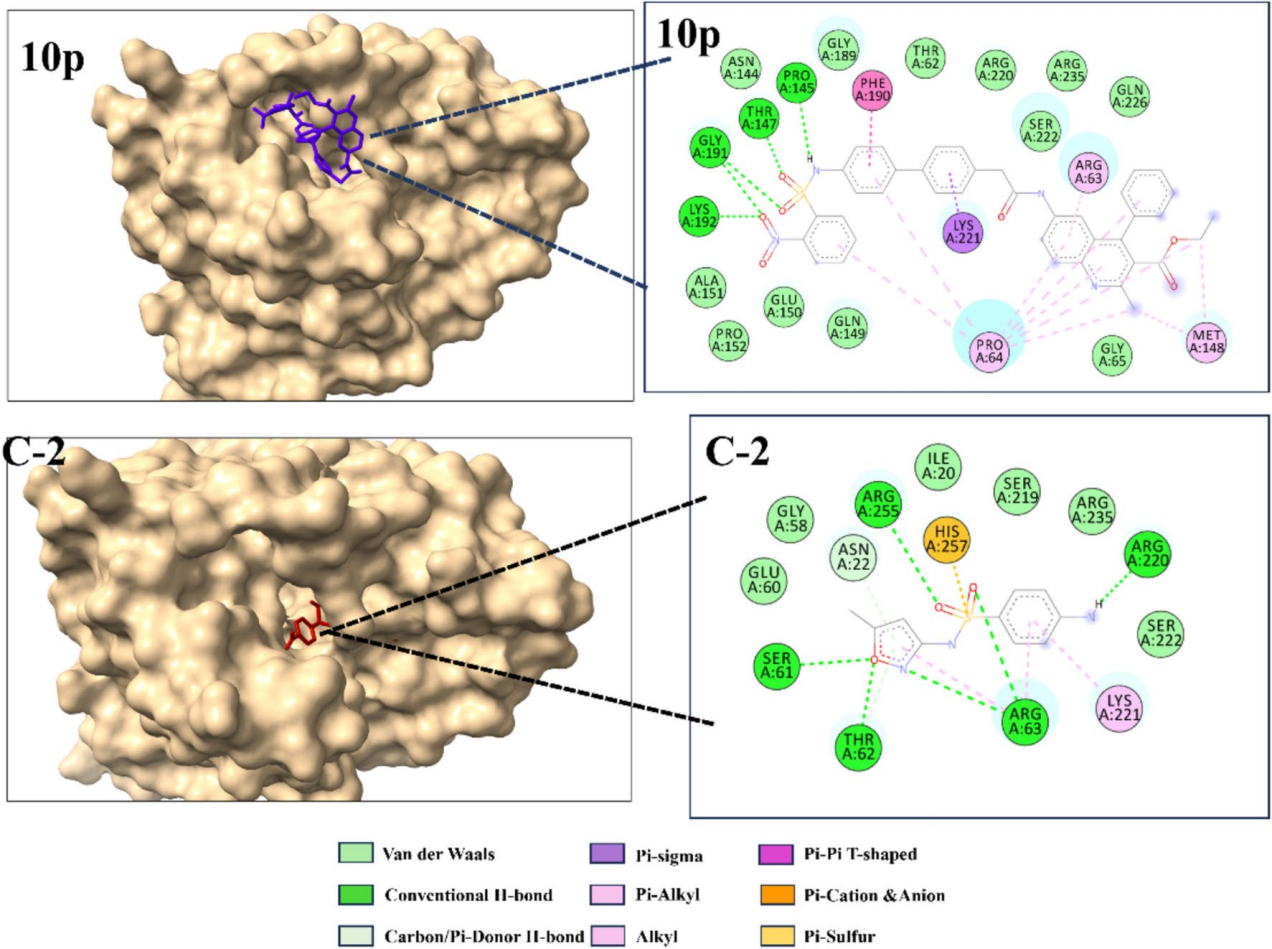
Depicted 2D and 3D interaction of compound **10p** and studied crystal structures by docking simulations **C-2 = **Sulfamethoxazole (control) against DHPS related protein (PDB: 1AB4).

Quinolones are a class of antibiotics that impede bacterial DNA synthesis through their interactions with DNA gyrase and topoisomerase IV, both of which are crucial enzymes in the process of DNA replication. DNA gyrase, encoded by gyrA, is the primary molecular target of quinolone antibiotics. In the present study, gyrA was identified as a pivotal target for assessing the efficacy of the newly synthesized quinoline derivatives. The quinolone resistance-determining region (QRDR) of gyrA in Escherichia coli spans amino acids 67–106, with modifications at positions 83 and 87 commonly linked to clinical resistance. The specific QRDR location in gyrA plays a crucial role in the effectiveness of quinolone-based antibiotics, as point mutations within this region facilitate resistance to these drugs^70,71^. Given its importance, we calculated the center of mass for this position to identify the active site. Due to the small HOMO–LUMO energy gap, compound **10** exhibits higher chemical and biological reactivity, making it a promising candidate for further studies^72,73^. The reduced gap suggests enhanced electron transfer capabilities, which can influence molecular interactions and stability. Considering these properties, compound **10p** was selected for molecular docking and ADMET analysis to evaluate its binding affinity, pharmacokinetic behavior, and potential biological applications. The molecular docking results revealed that compound **10p** exhibited superior binding affinity compared to standard drugs. Against the 1AB4 protein, compound **10p** showed a binding energy of − 8.01 kcal/mol, outperforming ciprofloxacin (− 6.3 kcal/mol). Similarly, when docked with the DHPS target, compound 10p demonstrated a significantly higher binding affinity of − 10.81 kcal/mol, surpassing the standard drug sulfamethoxazole (− 6.93 kcal/mol). These findings indicate that compound 10p has a stronger interaction with both target proteins docking binding affinity and 2D and 3D interaction shown Table 4 and Figs. 9, 10 respectively.

**Table 4:** Molecular docking compound **10p** interaction with target protein.

Compound	Target (PDB code)	Binding affinities (kcal mol^−1^)	Inhibition constant (μM)
**10P**	1AJ0	–8.01	26.94
Ciprofloxacin	1AJ0	–6.23	1.35
**10P**	1AB4	–10.81	0.011
Sulfamethoxazole	1AB4	–6.93	8.31

## ADMET properties

**Fig. 11:**
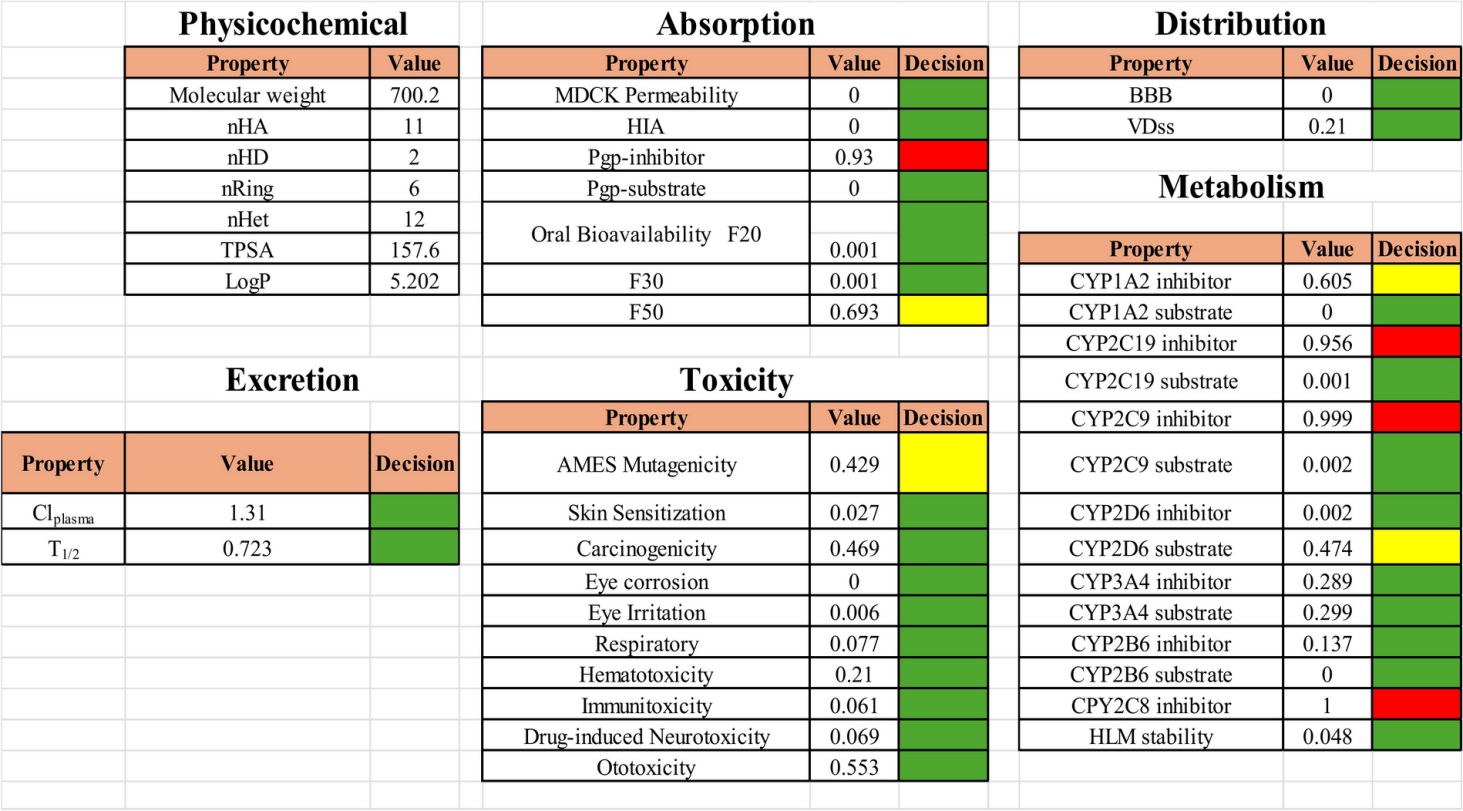
ADMET properties of Compound **10p** (green colour: excellent, yellow colour: medium, red colour: poor).

ADMET investigations assess a pharmaceutical compound’s absorption, distribution, metabolism, excretion, and toxicity, thereby confirming its pharmacokinetic integrity and safety. These evaluations facilitate the anticipation of bioavailability, tissue distribution, metabolic routes, clearance rates, and possible toxicity, which is instrumental in the optimization of pharmaceuticals for clinical advancement and the enhancement of therapeutic efficacy while mitigating adverse effects^74,75^. The absorption properties of compound **10p** indicate excellent MDCK permeability, HIA, and oral bioavailability at F20 and F30, while F50 shows moderate absorption. However, its Pgp-inhibitor potential is poor. The distribution properties of compound **10p** indicate excellent blood-brain barrier (BBB) permeability and volume of distribution (VDss), suggesting strong potential for central nervous system activity. In terms of metabolism, compound **10p** exhibits a medium CYP1A2 inhibition value (0.605) but is an excellent CYP1A2 substrate. It shows poor inhibition for CYP2C19 (0.956) but is an excellent substrate (0.001). Additionally, it has excellent inhibition (0.289) and substrate (0.299) values for CYP3A4, along with high hepatic metabolic stability (HLM stability = 0.048). Excretion properties are also promising, with excellent plasma clearance (Clplasma = 1.31) and half-life (T1/2 = 0.723). Regarding toxicity, compound **10p** shows medium AMES mutagenicity but excellent safety profiles for skin sensitization, carcinogenicity, eye corrosion, irritation, respiratory toxicity, hematotoxicity, immunotoxicity, drug-induced neurotoxicity, and ototoxicity. These findings suggest that compound **10p** possesses favorable pharmacokinetic shown in Figure 11.

## Experimental

### Methods and materials

The experiments were carried out in round bottom flasks, with all solvents and reagents sourced from commercial suppliers. The synthesis of N-(3-acetyl-2-methyl-4-phenylquinolin-6-yl)-2-(4ʹ-amino-[1,1ʹ-biphenyl]-4-yl)acetamide and ethyl 6-(2-(4ʹ-amino-[1,1ʹ-biphenyl]-4-yl)acetamido)-2-methyl-4-phenylquinoline-3-carboxylate was conducted according to established procedures. Utilizing a Bruker Avance 400 spectrometer, the ^1^H and ^13^C NMR spectra were acquired and calibrated against the residual solvent signal CDCl_3_: (7.26) for 1H and (77.16) for ^13^C NMR; dimethyl sulfoxide-d6 (2.50) for ^1^H and (39.50) for ^13^C. Chemical shifts (δ) were reported in parts per million, while coupling constants (*J*) were determined in Hertz. Nomenclature included s-*singlet*, d-*doublet*, dd-*doublet* of the *doublet*, t-*triplet*, m-*multiple*, and br-*broad*. High-Resolution Electrospray Ionization Mass Spectrometry (HR ESI–MS) data were generated using Thermo EXACTIVE Orbitrap high resolution mass spectrometer with Accela 600 UPLC system (Waters) and presented as m/z values. Absorption measurements were conducted utilizing a JASCO V-670 spectrometer. Fluorescence emission spectra were recorded on the Hitachi F-7000 FL spectroflurophotometer with excitation at the appropriate absorption maxima. Silica gel (100–200 mesh) packed glass columns were employed for column chromatography. Macherey–Nagel 60 F245 aluminium-backed silica gel plates were used for analytical Thin-Layer Chromatography (TLC).

Calculated all global reactivity parameters below equations.

1 $$DE\, = \,LUMO - HOMO \, \left( {eV} \right)$$

2 $$I \, = \, - E_{HOMO} \left( {eV} \right)$$

3 $$A \, = \, - E_{LUMO} \left( {eV} \right)$$

4 $$\chi = \, (I\, + \,A)/2 \, \left( {eV} \right)$$

5 $$\mu = \, - \chi \left( {eV} \right)$$

6 $$\eta = \, (I{-}A)/2 \, \left( {eV} \right)$$

7 $$S = {1 \mathord{\left/ {\vphantom {1 {\eta \left( {eV} \right)}}} \right. \kern-0pt} {\eta \left( {eV} \right)}}$$

8 $$\omega \, = \,\mu^{2} /2\eta \left( {eV} \right)$$

9 $$\omega^{ - } = \, (3I\, + \,A)^{2} /16\left( {I - A} \right)$$

10 $$\omega^{ + } = \, (I\, + \,3A)^{2} /16\left( {I - A} \right)$$

Quantum yield calculated by below equation

11 $$\phi f \, = \phi R. \, AR/A. \, I/IR. \, n^{2} R/n^{2}$$

### Procedure for compound (8)

Initially, Compound N-(3-acetyl-4-phenylquinolin-6-yl)-2-(4-bromophenyl) acetamide or ethyl 6-(2-(4-bromophenyl)acetamido)-4-phenylquinoline-3-carboxylate is mixed with 4-amino phenyl boronic acid and K_2_CO_3_ in a AR grade solvent 1, 4-dioxane-water (1:2) 10 mL at rt. followed by reaction mixture degassing with nitrogen gas. A palladium catalyst is added in reaction mixture and the reaction mixture is heated at 100 °C for 6 h. Afterward, the reaction is quenched with cold water (50 mL), and the organic layer is extracted with ethyl acetate (100 mL × 2). Drying with sodium sulfate removes residual water, and the concentrated organic layer is subjected to high vacuum to obtain a crude compound. This crude compound is further purified using column chromatography to isolate the desired pure product N-(3-acetyl-2-methyl-4-phenylquinolin-6-yl)-2-(4ʹ-amino-[1,1ʹ-biphenyl]-4-yl)acetamide and ethyl 6-(2-(4ʹ-amino-[1,1ʹ-biphenyl]-4-yl)acetamido)-2-methyl-4-phenylquinoline-3-carboxylate. Careful handling and safety precautions are essential due to the involvement of chemicals and reactive conditions in the process.

### General procedure for the synthesis of 10a-10p

N-(3-acetyl-2-methyl-4-phenylquinolin-6-yl)-2-(4ʹ-amino-[1,1ʹ-biphenyl]-4-yl) acetamide and ethyl 6-(2-(4ʹ-amino-[1,1ʹ-biphenyl]-4-yl)acetamido)-2-methyl-4-phenyquinoline-3-carboxylate (300–500 mgs), substituted sulphonyl chloride (1.5 to 3.0 equiv), DIPEA (2.5 equivalents) in 10 mL of AR grade DMF 50 mL round bottom flask was stirred at room temperature under the nitrogen gas for 20–30 min. After completion of the reaction, the reaction mixture was added to the cold water and extracted with ethyl acetate (2 × 25 mL). The organic layer was washed with brine water (25 mL). The Organic layer concentrates under a high vacuum to get crude compound. The crude compound was purified by using column chromatography to obtain pure products **10a**-**10p**. The all-synthesized derivatives experimental procedure, identification data and spectra shown in supplementary information.

## Conclusion

The synthesis of novel quinoline-sulphonamide derivatives was successfully completed using acid–amine coupling, Suzuki cross-coupling, and alkylation reactions. These methods introduced a variety of C_6_ substituents on the quinoline core, increasing the structural diversity of the compounds. These modifications helped refine Density Functional Theory (DFT) studies and enabled a detailed analysis of the photophysical properties of the synthesized derivatives. Time-Dependent Density Functional Theory (TD-DFT) calculations confirmed a small but significant difference in λmax values between theoretical predictions and experimental results, highlighting the accuracy of the models used. Additionally, the **10p** compound demonstrated strong binding affinity to two target proteins, comparable to a standard drug. Ongoing research is now focused on further exploring the unique characteristics of these derivatives, particularly their potential in fluorescence and medicinal applications.

## Supplementary Information

Supplementary Information.

## Data Availability

The data is provided within the manuscript along with the supplementary information file.
