# Supplementary material for: Synthesis of novel sulphonamide derivatives from tunable quinolines with computational studies
Source: Sci Rep. 2025 Mar 31;15:10972. doi: 10.1038/s41598-025-94817-1 (PMC11958747; doi:10.1038/s41598-025-94817-1)
Supplement: Supplementary file 1 — Supplementary Information. [file 41598_2025_94817_MOESM1_ESM.pdf]

Supportive Information

Synthesis of Novel Sulphonamide derivatives from Tunable Quinolines with Computational studies.

*Nagesh Dhanaji Chavan,[1] S Sarveswari,[1] V Vijayakumar[1]**

[†]Department of Chemistry, School of Advance Sciences, Vellore Institute of Technology, Vellore- 632014, India

Table of Contents

1. General Methods……………………………………………………….....S1
2. Synthesis of Functionalized Quinoline and Sulphonamide derivatives and Identification data …………………………………………………………………......S2
3. DFT (FMO, MEP) Calculations and Diagrams………………...……………..S10
4. ^{1}H, ^{13}C, DEPT-135 and 2D NMR and HRMS Spectra…………………..……S20

1. General methods

The experiments were carried out in round bottom flasks, with all solvents and reagents sourced from commercial suppliers. The synthesis of N-(3-acetyl-2-methyl-4-phenylquinolin-6-yl)-2-(4'-amino-[1,1'-biphenyl]-4-yl)acetamide and ethyl 6-(2-(4'-amino-[1,1'-biphenyl]-4-yl)acetamido)-2-methyl-4-phenylquinoline-3-carboxylate was conducted according to established procedures. Utilizing a Bruker Avance 400 spectrometer, the 1H and 13C NMR spectra were acquired and calibrated against the residual solvent signal CDCl3: (7.26) for 1H and (77.16) for 13C NMR; dimethyl sulfoxide-d6 (2.50) for 1H and (39.50) for 13C. Chemical shifts (δ) were reported in parts per million, while coupling constants (J) were determined in Hertz. Nomenclature included s-singlet, d-doublet, dd-doublet of the doublet, t-triplet, m-multiple, and br-broad. High-Resolution Electrospray Ionization Mass Spectrometry (HR ESI-MS) data were generated using Thermo EXACTIVE Orbitrap high resolution mass spectrometer with Accela 600 UPLC system (Waters) and presented as m/z values. Absorption measurements were conducted utilizing a JASCO V-670 spectrometer.

Fluorescence emission spectra were recorded on the Hitachi F-7000 FL spectroflurophotometer with excitation at the appropriate absorption maxima. Silica gel (100-200 mesh) packed glass columns were employed for column chromatography. Macherey-Nagel 60 F245 aluminium-backed silica gel plates were used for analytical Thin-Layer Chromatography (TLC).

2. Synthesis of Functionalized Quinoline

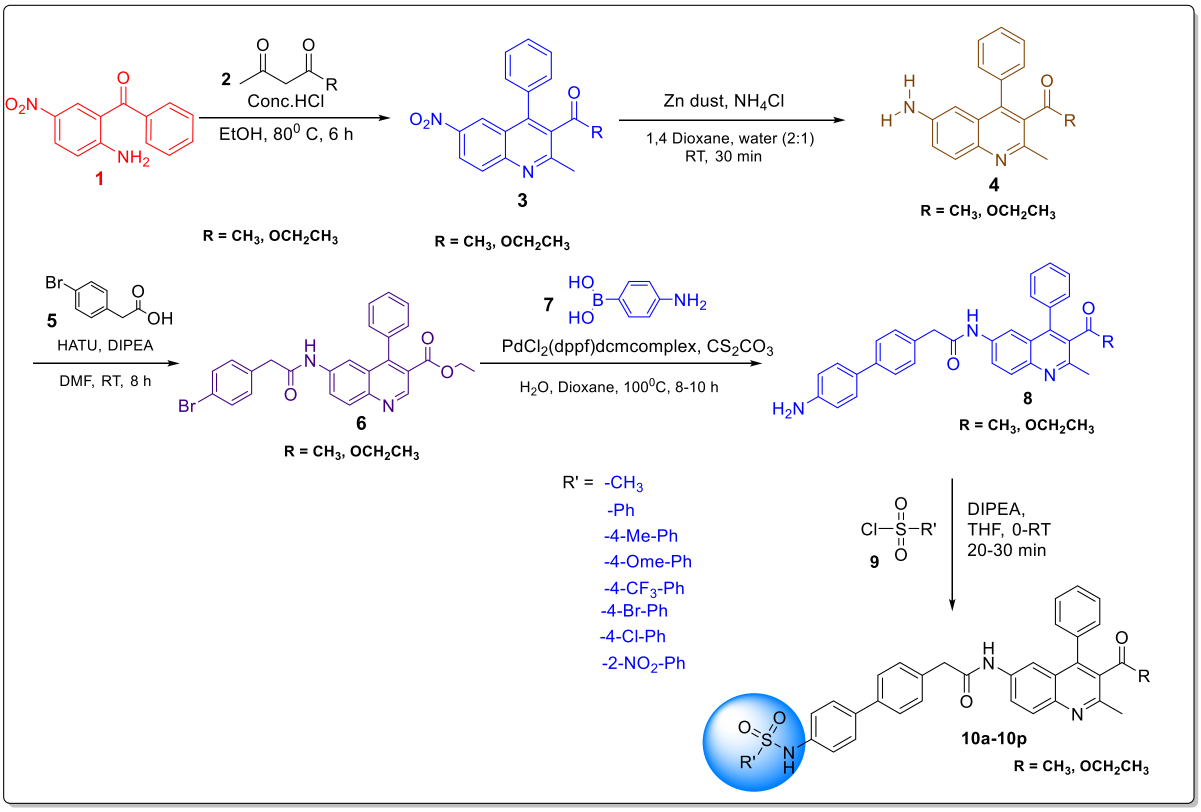

Procedure for compound (8) Initially, Compound N-(3-acetyl-4-phenylquinolin-6-yl)-2-(4-bromophenyl) acetamide or ethyl 6-(2-(4-bromophenyl) acetamido)-4-phenylquinoline-3-carboxylate is mixed with 4-amino phenyl boronic acid and K_2CO_3 in a AR grade solvent 1, 4 dioxane-water (1:2) 10 mL at rt. followed by reaction mixture degassing with nitrogen gas. A palladium catalyst is added in reaction mixture and the reaction mixture is heated at 100°C for 6 hours. Afterward, the reaction is quenched with cold water (50 mL), and the organic layer is extracted with ethyl acetate (100 mL × 2). Drying with sodium sulfate removes residual water, and the concentrated organic layer is subjected to high vacuum to obtain a crude compound. This crude compound is further purified using column chromatography to isolate the desired pure product N-(3-acetyl-2-methyl-4-phenylquinolin-6-yl)-2-(4'-amino-[1,1'-biphenyl]-4-yl)acetamide and ethyl 6-(2-(4'-amino-[1,1'-biphenyl]-4-yl)acetamido)-2-methyl-4-phenylquinoline-3-carboxylate. Careful handling and safety precautions are essential due to the involvement of chemicals and reactive conditions in the process.

2.1 General procedure for the synthesis of 10a-10p

N-(3-acetyl-2-methyl-4-phenylquinolin-6-yl)-2-(4'-amino-[1,1'-biphenyl]-4-yl)acetamide and ethyl 6-(2-(4'-amino-[1,1'-biphenyl]-4-yl)acetamido)-2-methyl-4-phenylquinoline-3-carboxylate (300-500 mgs), substituted sulphonyl chloride (1.5 to 3.0 equiv), DIPEA (2.5 equivalents) in 10 mL of AR grade DMF 50 mL round bottom flask was stirred at room temperature under the nitrogen gas for 20-30 mins. After completion of the reaction, the reaction mixture was added to the cold water and extracted with ethyl acetate (2 × 25 mL). The organic layer was washed with brine water (25mL). The Organic layer concentrates under a high vacuum to get crude compound. The crude compound was purified by using column chromatography to obtain pure products 10a-10p.

2.2 Procedure and Identification Analytical data of the compounds

N-(3-acetyl-2-methyl-4-phenylquinolin-6-yl)-2-(4'-(methylsulfonamido)-[1,1'-biphenyl]-4-yl)acetamide (10a) N-(3-acetyl-2-methyl-4-phenylquinolin-6-yl)-2-(4'-amino-[1,1'-biphenyl]-4-yl)acetamide (100 mg, 0.2183 mmole), methanesulfonyl chloride (123 mg, 1.0917 mmole), DIPEA (84 mg, 0.6550 mmole) in THF (5 mL) at RT for 20 min. The title compound was isolated (Eluent 60% ethyl acetate in Hexane). TLC: Rf = 0.3 (EtOAc/Hexane 6:4) [silica gel, UV and $KMnO_4$ stain]. White solid, yield 70%, m.p: 128-130^0 C. **^{1}H NMR** (400 MHz, DMSO-*d$_6$*): δ ppm 10.50 (bs, 1H), 9.84 (bs, 1H), 8.02 (dd, *J* = 1.60, 9.20 Hz, 1H), 7.99 (s, 1H), 7.96 (d, *J* = 4.80 Hz, 1H), 7.62 (d, *J* = 8.40 Hz, 1H), 7.58-5.57 (m, 5H), 7.36 (d, *J* = 8.00 Hz, 2H), 7.34-7.33 (m, 2H), 7.28 (d, *J* = 8.40 Hz, 1H), 3.66 (s, 2H), 3.02 (s, 3H), 2.56 (s, 3H), 2.00 (s, 3H). **^{13}C NMR** (100 MHz, DMSO-*d$_6$*): δ ppm 205.73, 169.90, 151.84, 143.97, 143.28, 138.32, 138.15, 138.09, 135.86, 135.41, 135.27, 135.11, 130.25, 130.12, 129.44, 129.24, 127.88, 126.75, 125.49, 124.22, 120.54, 113.40, 43.30, 39.26, 32.25, 23.56. **DEPT-135**: Positive peaks: - δ ppm 130.25, 130.12, 129.44, 129.24, 127.88, 126.75, 124.22, 120.53, 113.39. Negative Peak: - 43.30; **HRMS** (ESI) m/z: [M + H] + Calculated for $C_{33}H_{30}N_3O_4S$: 564.1857; Found 564.1894.

N-(3-acetyl-2-methyl-4-phenylquinolin-6-yl)-2-(4'-(phenylsulfonamido)-[1,1'-biphenyl]-4-yl)acetamide (10b). N-(3-acetyl-2-methyl-4-phenylquinolin-6-yl)-2-(4'-amino-[1,1'-biphenyl]-4-yl)acetamide (150 mg, 0.3275 mmole), benzenesulfonyl chloride (115 mg, 0.6550 mmole), DIPEA (126 mg, 0.9825 mmole) in THF (10 mL) at RT for 20 min. The title compound was isolated (Eluent 50% ethyl acetate in Hexane). TLC: Rf = 0.4 (EtOAc/Hexane 6:4) [silica gel, UV and $KMnO_4$ stain]. White solid, yield 80 %, m.p: 124-126^0 C. **^{1}H NMR** (400 MHz, DMSO-*d$_6$*): δ ppm 10.46 (s, 1H), 10.42 (s, 1H), 8.04-8.02 (m, 1H), 7.98 (d, *J* = 8.80 Hz, 1H), 7.93 (s, 1H), 7.61-7.60 (m, 10H), 7.34-7.32 (m, 4H), 7.19 (d, *J* = 8.40 Hz, 2H), 3.65 (s, 2H), 2.56 (s, 3H), 2.00 (s, 3H). **^{13}C NMR** (100 MHz, DMSO-*d$_6$*): δ ppm 205.72, 169.82,

151.81, 144.23, 143.06, 140.12, 138.04, 137.44, 136.02, 135.41, 135.36, 135.17, 133.39, 130.18, 130.14, 129.76, 129.56, 129.36, 129.19, 127.69, 127.11, 126.69, 125.45, 124.15, 120.79, 113.45, 43.31, 32.22, 23.64. **DEPT-135**: Positive peaks:- δ ppm 133.39, 130.18, 130.14, 129.76, 129.57, 129.36, 129.19, 127.69, 127.11, 126.69, 124.15, 120.79, 113.45; Negative Peak:- 43.31; **HRMS** (ESI) m/z: [M + H] + Calculated for $C_{38}H_{32}N_3O_4S$: 626.2113; Found 626.2065.

N-(3-acetyl-2-methyl-4-phenylquinolin-6-yl)-2-(4'-((4-methylphenyl)sulfonamido)-[1,1'-biphenyl]-4-yl)acetamide (10c). N-(3-acetyl-2-methyl-4-phenylquinolin-6-yl)-2-(4'-amino-[1,1'-biphenyl]-4-yl)acetamide (150 mg, 0.3275 mmole), 4-methyl benzenesulfonyl chloride (115 mg, 0.6550 mmole), DIPEA (126 mg, 0.9825 mmole) in THF (10 mL) at RT for 20 min. The title compound was isolated (Eluent 50% ethyl acetate in Hexane). TLC: Rf = 0.4 (EtOAc/Hexane 6:4) [silica gel, UV and $KMnO_4$ stain]. White solid, yield 82 %, m.p: 108-110^0 C. **^{1}H NMR** (400 MHz, DMSO-d_6): δ ppm 10.46 (s, 1H), 10.42 (s, 1H), 8.04-8.02 (m, 1H), 7.98 (d, *J* = 8.80 Hz, 1H), 7.93 (s, 1H), 7.61-7.60 (m, 10H), 7.34-7.32 (m, 4H), 7.19 (d, *J* = 8.40 Hz, 2H), 3.65 (s, 2H), 2.56 (s, 3H), 2.00 (s, 3H). **^{13}C NMR** (100 MHz, DMSO-d_6): δ 205.76, 169.83, 151.81, 144.21, 143.77, 143.05, 138.11, 138.02, 137.57, 137.22, 135.84, 135.40, 135.34, 135.13, 130.20, 130.13, 129.57, 129.37, 129.20, 127.67, 127.16, 126.67, 127.16, 126.67, 125.44, 124.12, 120.57, 113.41, 43.29, 32.24, 23.65, 21.40. **DEPT-135**: Positive peaks:- δ ppm 133.20, 130.13, 129.57, 129.37, 129.21, 127.17, 126.67, 124.12, 120.56, 113.40, 32.24, 23.65, 21.41; Negative Peak:- 43.29; **HRMS** (ESI) m/z: [M + H] + Calculated for $C_{39}H_{34}N_3O_4S$; 640.2270; Found 640.2250.

N-(3-acetyl-2-methyl-4-phenylquinolin-6-yl)-2-(4'-((4-methoxyphenyl)sulfonamido)-[1,1'-biphenyl]-4-yl)acetamide (10d). N-(3-acetyl-2-methyl-4-phenylquinolin-6-yl)-2-(4'-amino-[1,1'-biphenyl]-4-yl)acetamide (150 mg, 0.3275 mmole), 4-methoxy benzenesulfonyl chloride (134 mg, 0.6550 mmole), DIPEA (126 mg, 0.9825 mmole) in THF (10 mL) at RT for 20 min. The title compound was isolated (Eluent 50% ethyl acetate in Hexane). TLC: Rf = 0.4 (EtOAc/Hexane 6:4) [silica gel, UV and $KMnO_4$ stain]. Light yellow solid, yield 86 %, m.p: 140-142^0 C. **^{1}H NMR** (400 MHz, DMSO-d_6): δ ppm 10.45 (s, 1H), 10.26 (s, 1H), 8.03-8.01 (m, 2H), 7.96-7.93 (m, 1H), 7.74 (d, *J* = 8.80 Hz, 2H), 7.55-7.52 (m, 7H), 7.32-7.33 (m, 4H), 7.17 (d, *J* = 8.00 Hz, 2H), 7.07 (d, *J* = 8.40 Hz, 2H), 3.79 (s, 3H), 3.65 (s, 2H), 2.56 (s, 3H), 2.00 (s, 3H). **^{13}C NMR** (100 MHz, DMSO-d_6): δ ppm 205.76, 169.84, 162.91, 151.81, 144.22, 143.06, 138.14, 138.03, 137.69, 135.76, 135.40, 135.34, 135.12, 131.68, 130.18, 130.13, 129.57, 129.36, 129.20, 127.65, 126.66, 125.44, 124.13, 120.53, 114.89, 113.42, 56.07, 43.30, 32.23, 23.64. **DEPT-135**: Positive peaks:- δ ppm 130.19, 130.13, 129.57, 129.36, 129.21,

127.66, 126.66, 124.13, 120.52, 114.89, 113.41, 56.07, 32.24, 23.65; Negative Peak:- 43.30; HRMS (ESI) m/z: [M + H] + Calculated for $C_{39}H_{34}N_3O_5S$; 656.2219; Found 656.2180

N-(3-acetyl-2-methyl-4-phenylquinolin-6-yl)-2-(4'-((4(trifluoromethyl)phenyl)sulfonamido)-[1,1'-biphenyl]-4-yl)acetamide (10e). N-(3-acetyl-2-methyl-4-phenylquinolin-6-yl)-2-(4'-amino-[1,1'-biphenyl]-4-yl)acetamide (150mg, 0.3275 mmole), 4-trifluro benzenesulfonyl chloride (159 mg, 0.6550 mmole), DIPEA (126 mg, 0.9825 mmole) in THF (10 mL) at RT for 20 min. The title compound was isolated (Eluent 55% ethyl acetate in Hexane). TLC: Rf = 0.5 (EtOAc/Hexane 6:4) [silica gel, UV and $KMnO_4$ stain]. White solid, yield 86 %, m.p: 128-130^0 C. **^{1}H NMR** (400 MHz, DMSO-*d$_6$*): δ ppm 10.65 (s, 1H), 10.46 (s, 1H), 8.02-7.97 (m, 6H), 7.54-7.54 (m, 7H), 7.33-7.21 (m, 6H), 3.66 (s, 2H), 2.56 (s, 3H), 1.99 (s, 3H). **^{13}C NMR** (100 MHz, DMSO-*d$_6$*): δ ppm 205.76, 169.84, 162.91, 151.81, 144.22, 143.06, 138.14, 138.03, 137.69, 135.76, 135.40, 135.34, 135.12, 131.68, 130.18, 130.13, 129.57, 129.36, 129.20, 127.65, 126.66, 125.44, 124.13, 120.53, 114.89, 113.42, 56.07, 43.30, 32.23, 23.64. **DEPT-135**: Positive peaks:- δ ppm 130.19, 130.13, 129.57, 129.36, 129.21, 127.66, 126.66, 124.13, 120.52, 114.89, 113.41, 56.07, 32.24, 23.65; Negative Peak:- 43.30; HRMS (ESI) m/z: [M + H] + Calculated for $C_{39}H_{31}F_3N_3O_4S$: 694.1987; Found 694.1927

N-(3-acetyl-2-methyl-4-phenylquinolin-6-yl)-2-(4'-((4-bromophenyl)sulfonamido)-[1,1'-biphenyl]-4-yl)acetamide (10f). N-(3-acetyl-2-methyl-4-phenylquinolin-6-yl)-2-(4'-amino-[1,1'-biphenyl]-4-yl)acetamide (150mg, 0.3275 mmole), 4-Bromo benzenesulfonyl chloride (165 mg, 0.6550 mmole), DIPEA (126 mg, 0.9825 mmole) in THF (10 mL) at RT for 20 min. The title compound was isolated (Eluent 55% ethyl acetate in Hexane). TLC: Rf = 0.5 (EtOAc/Hexane 6:4) [silica gel, UV and $KMnO_4$ stain]. White solid, yield 80%, m.p: 104-106^0 C. **^{1}H NMR** (400 MHz, DMSO-*d$_6$*): δ ppm 10.52 (s, 1H), 7.95-7.95 (m, 3H), 7.77-7.73 (m, 4H), 7.54-7.54 (m, 7H), 7.33-7.17 (m, 6H), 3.65 (s, 2H), 2.51 (s, 3H), 1.99 (s, 3H). **^{13}C NMR** (100 MHz, DMSO-*d$_6$*): δ ppm 205.79, 169.85, 151.80, 144.20, 143.06, 139.32, 138.04, 137.16, 136.26, 135.38, 135.24, 132.89, 130.21, 130.13, 129.55, 129.37, 129.21, 129.15, 127.78, 127.30, 126.71, 125.44, 124.14, 121.06, 113.39, 43.29, 32.24, 23.65 **DEPT-135**: Positive peaks: - δ ppm 132.89, 130.21, 130.13, 129.55, 129.38, 129.21, 129.15, 127.78, 126.71, 124.14, 121.06, 113.39, 32.24, 23.65. Negative Peak: - 43.29. **HRMS** (ESI) m/z: [M + H] + Calculated for $C_{38}H_{31}BrN_3O_4S$; 704.1218; Found 704.1170

N-(3-acetyl-2-methyl-4-phenylquinolin-6-yl)-2-(4'-((4-chlorophenyl)sulfonamido)-[1,1'-biphenyl]-4-yl)acetamide (10g) N-(3-acetyl-2-methyl-4-phenylquinolin-6-yl)-2-(4'-amino-[1,1'-biphenyl]-4-yl)acetamide (150 mg, 0.3275 mmole), 4-Chloro benzenesulfonyl chloride (171 mg, 0.8187 mmole), DIPEA (126 mg, 0.9825 mmole) in THF (10 mL) at RT for 20 min.

The title compound was isolated (Eluent 55% ethyl acetate in Hexane). TLC: Rf = 0.5 (EtOAc/Hexane 6:4) [silica gel, UV and $KMnO_4$ stain]. Pale yellow solid, yield 78 %, m.p: 120-122^0 C. **^{1}H NMR** (400 MHz, DMSO-d_6): δ ppm δ 10.47 (s, 1H), 10.44 (s, 1H), 8.01 (dd, *J* = 1.60, 8.80 Hz, 1H), 7.97 (d, *J* = 8.80 Hz, 1H), 7.92 (s, 1H), 7.79 (d, *J* = 8.40 Hz, 2H), 7.64 (d, *J* = 8.40 Hz, 2H), 7.56-7.55 (m, 7H), 7.34-7.33 (m, 4H), 7.16 (d, *J* = 8.40 Hz, 2H), 3.64 (s, 2H), 2.56 (s, 3H), 2.00 (s, 3H). **^{13}C NMR** (100 MHz, DMSO-d_6): δ ppm 205.71, 169.81, 151.81, 144.19, 143.08, 138.88, 138.32, 138.04, 137.08, 136.32, 135.41, 135.43, 135.24, 130.48, 130.20, 130.14, 129.96, 129.55, 129.37, 129.20, 129.07, 127.78, 126.72, 125.45, 124.13, 121.08, 113.42, 43.29, 32.24, 23.64 **DEPT-135**: Positive peaks: - δ ppm 130.48, 130.37, 130.20, 130.14, 129.97, 129.55, 129.37, 129.21, 129.07, 127.78, 126.72, 124.13, 121.08, 113.41, 32.24, 23.64. Negative Peak: - 43.29. **HRMS** (ESI) m/z: [M + H] + Calculated for $C_{38}H_{31}ClN_3O_4S$: 660.1723; Found 660.1697.

N-(3-acetyl-2-methyl-4-phenylquinolin-6-yl)-2-(4'-((2-nitrophenyl)sulfonamido)-[1,1'-biphenyl]-4-yl)acetamide (10h). N-(3-acetyl-2-methyl-4-phenylquinolin-6-yl)-2-(4'-amino-[1,1'-biphenyl]-4-yl)acetamide (150mg, 0.3275 mmole), 4-Nitro benzenesulfonyl chloride (144.1 mg, 0.6560 mmole), DIPEA (126 mg, 0.9825 mmole) in THF (10 mL) at RT for 20 min. The title compound was isolated (Eluent 55% ethyl acetate in Hexane). TLC: Rf = 0.5 (EtOAc/Hexane 6:4) [silica gel, UV and $KMnO_4$ stain]. White solid, yield 90%, m.p: 132-134^0 C. **^{1}H NMR** (400 MHz, DMSO-d_6): δ ppm 10.47 (s, 1H), 10.44 (s, 1H), 8.01 (dd, *J* = 1.60, 8.80 Hz, 1H), 7.97 (d, *J* = 8.80 Hz, 1H), 7.92 (s, 1H), 7.79 (d, *J* = 8.40 Hz, 2H), 7.64 (d, *J* = 8.40 Hz, 2H), 7.56-7.55 (m, 7H), 7.34-7.33 (m, 4H), 7.16 (d, *J* = 8.40 Hz, 2H), 3.64 (s, 2H), 2.56 (s, 3H), 2.00 (s, 3H). **^{13}C NMR** (100 MHz, DMSO-d_6): δ ppm 205.71, 169.81, 151.81, 144.19, 143.08, 138.88, 138.32, 138.04, 137.08, 136.32, 135.41, 135.43, 135.24, 130.48, 130.20, 130.14, 129.96, 129.55, 129.37, 129.20, 129.07, 127.78, 126.72, 125.45, 124.13, 121.08, 113.42, 43.29, 32.24, 23.64 **DEPT-135**: Positive peaks: - δ ppm130.48, 130.37, 130.20, 130.14, 129.97, 129.55, 129.37, 129.21, 129.07, 127.78, 126.72, 124.13, 121.08, 113.41, 32.24, 23.64. Negative Peak: - 43.29. **HRMS** (ESI) m/z: [M + H] + Calculated for $C_{38}H_{31}N_4O_6S$; 671.1964; Found 671.1905.

Ethyl-2-methyl-6-(2-(4'-(methylsulfonamido)-[1,1'-biphenyl]-4-yl)acetamido)-4-phenyl quinoline-3-carboxylate (10i) Ethyl 6-(2-(4'-amino-[1,1'-biphenyl]-4-yl)acetamido)-2-methyl-4-phenylquinoline-3-carboxylate (150mg, 0.2912 mmole), Methane sulfonyl chloride (165.9 mg, 1.456 mmole), DIPEA (112 mg, 0.8736 mmole) in THF (10 mL) at RT for 20 min. The title compound was isolated (Eluent 55% ethyl acetate in Hexane). TLC: Rf = 0.5 (EtOAc/Hexane 6:4) [silica gel, UV and $KMnO_4$ stain]. Off White solid, yield 72%, m.p: 98-

100^0 C. **^{1}H NMR** (400 MHz, DMSO-*d*$_6$): δ ppm 10.49 (s, 1H), 9.83 (s, 1H), 8.05 (dd, *J* = 1.60, 9.20 Hz, 1H), 7.99 (d, *J* = 9.20 Hz, 1H), 7.94 (d, *J* = 1.60 Hz, 1H), 7.62 (d, *J* = 8.40 Hz, 2H), 7.57 (d, *J* = 8.00 Hz, 2H), 7.55-7.53 (m, 3H), 7.36 (d, *J* = 8.00 Hz, 2H), 7.33-7.33 (m, 4H), 4.01 (q, *J* = 7.20 Hz, 2H), 3.66 (s, 2H), 2.63 (s, H), 0.87 (t, *J* = 7.20 Hz, 3H). **^{13}C NMR** (100 MHz, DMSO-*d*$_6$): δ ppm 169.88, 168.13, 152.54, 145.28, 144.47, 138.33, 138.18, 138.12, 135.86, 135.12, 130.23, 129.66, 129.53, 129.06, 128.88, 127.87, 127.79, 126.75, 125.34, 124.47, 120.51, 113.59, 61.48, 43.32, 23.51, 13.88 **DEPT-135**: Positive peaks: - δ ppm 130.42, 129.65, 129.52, 129.07, 128.88, 127.87, 126.75, 124.48, 120.53, 113.60, 23.50, 13.88. Negative Peak: - 61.49, 43.31. **HRMS** (ESI) m/z: [M + H] + Calculated for $C_{34}H_{32}N_3O_5S$: 594.2062; Found: 594.2011.

Ethyl-2-methyl-4-phenyl-6-(2-(4'-(phenylsulfonamido)-[1,1'-biphenyl]-4-yl)acetamido) quinoline-3-carboxylate (10j). Ethyl 6-(2-(4'-amino-[1,1'-biphenyl]-4-yl)acetamido)-2-methyl-4-phenylquinoline-3-carboxylate (150mg, 0.2912 mmole), benzenesulfonyl chloride (152.8 mg, 0.8736 mmole), DIPEA (112 mg, 0.8736 mmole) in THF (10 mL) at RT for 20 min. The title compound was isolated (Eluent 55% ethyl acetate in Hexane). TLC: Rf = 0.5 (EtOAc/Hexane 6:4) [silica gel, UV and $KMnO_4$ stain]. White solid, yield 82%, m.p: 114-116^0 C. **^{1}H NMR** (400 MHz, DMSO-*d*$_6$): δ ppm 10.45 (s, 1H), 10.40 (s, 1H), 8.03 (dd, *J* = 2.40, 9.00 Hz, 1H), 7.98 (d, *J* = 8.80 Hz, 1H), 7.91 (d, *J* = 1.84 Hz, 1H), 7.80 (d, *J* = 8.28 Hz, 2H), 7.61-7.60 (m, 11H), 7.33-7.32 (m, 4H), 7.17 (d, *J* = 8.40 Hz, 2H), 4.00 (q, *J* = 6.80 Hz, 2H), 3.64 (s, 2H), 2.63 (s, 3H), 0.87 (t, *J* = 6.80 Hz, 3H). **^{13}C NMR** (100 MHz, DMSO-*d*$_6$): δ ppm 169.84, 168.13, 152.53, 145.25, 144.49, 140.05, 138.10, 137.43, 135.98, 135.65, 135.15, 133.42, 130.18, 129.79, 129.67, 129.52, 129.05, 128.87, 127.78, 127.71, 127.11, 126.69, 125.33, 124.45, 120.71, 113.58, 61.47, 43.29, 23.53, 13.88. **DEPT-135**: Positive peaks: - δ ppm 130.21, 129.66, 129.52, 129.04, 128.86, 128.13, 127.85, 127.11, 127.07, 126.73, 124.45, 121.17, 113.59, 23.51, 13.86. Negative Peak: - 61.47, 43.29. **HRMS** (ESI) m/z: [M + H] + Calculated for $C_{39}H_{34}N_3O_5S$; 656.2219; Found 656.2157

Ethyl 2-methyl-6-(2-(4'-((4-methylphenyl)sulfonamido)-[1,1'-biphenyl]-4-yl)acetamido)-4-phenylquinoline-3-carboxylate (10k). Ethyl 6-(2-(4'-amino-[1,1'-biphenyl]-4-yl)acetamido)-2-methyl-4-phenylquinoline-3-carboxylate (150 mg, 0.2912 mmole), 4-methylbenzenesulfonyl chloride (165.1 mg, 0.8736 mmole), DIPEA (112 mg, 0.8736 mmole) in THF (10 mL) at RT for 20 min. The title compound was isolated (Eluent 55% ethyl acetate in Hexane). TLC: Rf = 0.5 (EtOAc/Hexane 6:4) [silica gel, UV and $KMnO_4$ stain]. White solid, yield 84%, m.p: 104-106^0 C. **^{1}H NMR** (400 MHz, DMSO-*d*$_6$): δ ppm 10.45 (s, 1H), 10.32 (s, 1H), 8.04 (d, *J* = 7.20 Hz, 1H), 7.98 (d, *J* = 8.80 Hz, 1H), 7.92 (s, 1H), 7.69 (d, *J* = 8.00 Hz,

2H), 7.52-7.50 (m, 7H), 7.36-7.33 (m, 6H), 7.17 (d, *J* = 8.40 Hz, 2H), 3.92 (q, *J* = 7.20 Hz, 2H), 3.64 (s, 2H), 2.63 (s, 3H), 2.32 (s, 3H), 0.87 (t, *J* = 6.80 Hz, 3H) **^{13}C NMR** (100 MHz, DMSO-*d_6*): δ ppm 169.94, 168.13, 152.53, 145.25, 144.50, 143.75, 138.13, 138.09, 137.59, 137.27, 135.85, 135.66, 135.13, 130.19, 130.17, 129.98, 129.65, 129.04, 128.86, 127.80, 127.66, 127.49, 127.16, 126.67, 125.76, 125.34, 124.48, 120.59, 114.69, 113.63, 61.47, 43.29, 23.51, 21.40, 13.88 **DEPT-135**: Positive peaks: - δ ppm 130.20, 130.17, 129.98, 129.66, 129.53, 129.04, 128.86, 127.67, 127.49, 127.16, 126.67, 125.76, 124.47, 120.59, 114.69, 113.63, 23.51, 21.40, 13.88. Negative Peak: - 61.47, 43.29. **HRMS** (ESI) m/z: [M + H] + Calculated for $C_{40}H_{36}N_3O_5S$: 670.2375; Found 470.2315

Ethyl 6-(2-(4'-((4-methoxyphenyl)sulfonamido)-[1,1'-biphenyl]-4-yl)acetamido)-2-methyl-4-phenylquinoline-3-carboxylate (10l). Ethyl 6-(2-(4'-amino-[1,1'-biphenyl]-4-yl)acetamido)-2-methyl-4-phenylquinoline-3-carboxylate (150mg, 0.2912 mmole), 4-methylbenzenesulfonyl chloride (179.08 mg, 0.8736 mmole), DIPEA (112 mg, 0.8736 mmole) in THF (10 mL) at RT for 20 min. The title compound was isolated (Eluent 55% ethyl acetate in Hexane). TLC: Rf = 0.5 (EtOAc/Hexane 6:4) [silica gel, UV and $KMnO_4$ stain]. White solid, yield 86%, m.p: 138-140^0 C. **^{1}H NMR** (400 MHz, DMSO-*d_6*): δ ppm 10.46 (s, 1H), 10.27 (s, 1H), 8.06 (d, *J* = Hz, 1H), 7.99 (d, *J* = 8.80 Hz, 1H), 7.92 (s, 1H), 7.74 (d, *J* = 8.00 Hz, 2H), 7.52-7.52 (m, 7H), 7.32-7.32 (m, 4H), 7.18 (d, *J* = 7.60 Hz, 2H), 7.07 (d, *J* = 8.00 Hz, 2H), 4.01 (d, *J* = 6.80 Hz, 2H), 3.78 (s, 3H), 3.65 (s, 2H), 2.63 (s, 3H), 0.87 (t, *J* = 6.40 Hz, 3H). **^{13}C NMR** (100 MHz, DMSO-*d_6*): δ ppm 169.83, 168.13, 162.92, 152.53, 145.25, 144.53, 138.16, 138.11, 137.73, 135.78, 135.68, 135.13, 131.76, 130.17, 129.66, 129.54, 129.35, 129.03, 128.85, 127.80, 127.65, 126.66, 125.35, 124.47, 120.56, 114.89, 113.65, 61.45, 56, 07, 43.31, 23.52, 13.87. **DEPT-135**: Positive peaks: - δ ppm 130.17, 129.66, 129.54, 129.35, 129.03, 128.85, 127.65, 126.66, 124.47, 120.55, 114.89, 113.54, 56.07, 23.52, 13.87. Negative Peak: - 61.45, 43.31. **HRMS** (ESI) m/z: [M + H] + Calculated for $C_{40}H_{36}N_3O_6S$; 686.2324; Found: 686.2263.

Ethyl 2-methyl-4-phenyl-6-(2-(4'-((4-(trifluoromethyl)phenyl)sulfonamido)-[1,1'-biphenyl]-4-yl)acetamido)quinoline-3-carboxylate (10m) Ethyl 6-(2-(4'-amino-[1,1'-biphenyl]-4-yl)acetamido)-2-methyl-4-phenylquinoline-3-carboxylate (150 mg, 0.2912 mmole), 4-(trifluoromethyl)benzenesulfonyl chloride (176.90 mg, 0.728 mmole), DIPEA (112 mg, 0.8736 mmole) in THF (10 mL) at RT for 20 min. The title compound was isolated (Eluent 55% ethyl acetate in Hexane). TLC: Rf = 0.5 (EtOAc/Hexane 6:4) [silica gel, UV and $KMnO_4$ stain]. Brown e solid, yield 84%, m.p: 108-100^0 C. **^{1}H NMR** (400 MHz, DMSO-*d_6*): δ ppm 10.65 (s, 1H), 10.51 (s, 1H), 8.13 (d, *J* = 1.20 Hz, 1H), 8.06-8.05 (m, 6H), 7.35-7.33 (m, 4H), 7.19 (d, *J* = 8.40 Hz, 2H), 3.86 (q, *J* = 7.20 Hz, 2H), 3.65 (s, 2H), 2.63 (s, 3H), 0.87 (t, *J* = 7.20 Hz, 3H).

13C NMR (100 MHz, DMSO-d_6): δ ppm 169.84, 168.13, 152.53, 145.25, 144.49, 143.94, 138.09, 137.98, 136.84, 136.50, 135.65, 135.27, 130.21, 129.66, 129.52, 129.04, 128.86, 128.13, 127.84, 127.10, 127.07, 126.73, 125.33, 125.17, 124.45, 121.18, 113.59, 61.46, 43.29, 23.50, 13.85. **DEPT-135**: Positive peaks: - δ ppm 133.42, 130.19, 129.79, 129.67, 129.52, 129.05, 128.87, 127.71, 127.11, 126.70, 124.44, 120.70, 113.58, 23.29, 13.88. Negative Peak: - 61.47, 43.29. **HRMS** (ESI) m/z: [M + H] + Calculated for $C_{40}H_{33}F_3N_3O_5S$: 724.2093; Found 724.2037

Ethyl-6-(2-(4'-((4-bromophenyl)sulfonamido)-[1,1'-biphenyl]-4-yl)acetamido)-2-methyl-4-phenylquinoline-3-carboxylate (10n). Ethyl 6-(2-(4'-amino-[1,1'-biphenyl]-4-yl)acetamido)-2-methyl-4-phenylquinoline-3-carboxylate (150 mg, 0.2912 mmole), 4-bromobenzenesulfonyl chloride (162.08 mg, 0.6406 mmole), DIPEA (112 mg, 0.8736 mmole) in THF (10 mL) at RT for 20 min. The title compound was isolated (Eluent 55% ethyl acetate in Hexane). TLC: Rf = 0.5 (EtOAc/Hexane 6:4) [silica gel, UV and $KMnO_4$ stain]. Light yellow solid, yield 84%, m.p: 120-122^0 C. **1H NMR** (400 MHz, DMSO-d_6): δ ppm 10.49 (s, 1H), 10.48 (s, 1H), 8.05-8.03 (m, 3H), 7.78 (d, *J* = 8.00 Hz, 2H), 7.71 (d, *J* = 8.00 Hz, 2H), 7.53-7.52 (m, 7H), 7.34-7.32 (m, 4H), 7.17 (d, *J* = 8.00 Hz, 2H), 4.00 (d, *J* = 6.80 Hz, 2H), 3.64 (s, 2H), 2.63 (s, 3H), 0.86 (t, *J* = 6.40 Hz, 3H). **13C NMR** (100 MHz, DMSO-d_6): δ ppm 169.84, 168.13, 152.53, 145.25, 144.49, 139.28, 138.10, 138.04, 137.08, 136.31, 135.65, 135.22, 132.91, 130.20, 129.67, 129.52, 129.14, 129.05, 128.87, 127.80, 127.32, 126.73, 125.33, 124.45, 121.03, 113.59, 61.47, 43.29, 23.52, 13.87. **DEPT-135**: Positive peaks: - δ ppm 132.91, 130.20, 129.67, 129.52, 129.14, 129.05, 128.87, 127.80, 126.73, 124.45, 121.03, 113.59, 23.52, 13.87. Negative Peak: - 61.47, 43.29.

HRMS (ESI) m/z: [M + H] + Calculated for $C_{39}H_{33}BrN_3O_5S$; 734.1324; Found 734.1260

Ethyl 6-(2-(4'-((4-chlorophenyl)sulfonamido)-[1,1'-biphenyl]-4-yl)acetamido)-2-methyl-4-phenylquinoline-3-carboxylate (10o). Ethyl 6-(2-(4'-amino-[1,1'-biphenyl]-4-yl)acetamido)-2-methyl-4-phenylquinoline-3-carboxylate (150 mg, 0.2912 mmole), 4-chlorobenzenesulfonyl chloride (152.60 mg, 0.728 mmole), DIPEA (112 mg, 0.8736 mmole) in THF (10 mL) at RT for 20 min. The title compound was isolated (Eluent 55% ethyl acetate in Hexane). TLC: Rf = 0.5 (EtOAc/Hexane 6:4) [silica gel, UV and $KMnO_4$ stain]. Light yellow solid, yield 88%, m.p: 110-112^0 C. **1H NMR** (400 MHz, DMSO-*d6*): δ ppm 10.49 (s, 1H), 10.47 (s, 1H), 8.04 (dd, *J* = 2.00, 9.00 Hz, 1H), 7.99 (d, *J* = 8.80 Hz, 1H), 7.93 (d, *J* = 1.60 Hz, 1H), 7.80 (d, *J* = 8.40 Hz, 2H), 7.64-7.62 (m, 3H), 7.55-7.52 (m, 7H), 7.34-7.33 (m, 4H), 7.18 (d, *J* = 8.80 Hz, 2H), 4.00 (q, *J* = 6.80 Hz, 2H), 3.65 (s, 2H), 2.63 (s, 3H), 0.86 (t, *J* = 7.20 Hz, 3H). **13C NMR** (100 MHz, DMSO-d_6): δ ppm 169.85, 168.14, 152.54, 145.25, 144.49, 138.85, 138.33, 138.10, 138.04,

137.09, 136.32, 135.66, 135.22, 130.20, 129.96, 129.67, 129.52, 129.07, 128.86, 127.79, 126.72, 125.33, 124.45, 121.05, 113.60, 61.41, 43.30, 23.51, 13.86. **DEPT-135**: Positive peaks: - δ ppm 130.21, 129.96, 129.67, 129.52, 129.08, 128.86, 127.79, 126.72, 124.45, 121.05, 113.60, 23.52, 13.87. Negative Peak: - 61.47, 43.30. **HRMS** (ESI) m/z: [M + H] + Calculated for $C_{39}H_{33}ClN_3O_5S$: 690.1829; Found: 670.1367.

Ethyl 2-methyl-6-(2-(4'-((2-nitrophenyl)sulfonamido)-[1,1'-biphenyl]-4-yl)acetamido)-4-phenylquinoline-3-carboxylate (10p). Ethyl 6-(2-(4'-amino-[1,1'-biphenyl]-4-yl)acetamido)-2-methyl-4-phenylquinoline-3-carboxylate (150 mg, 0.2912 mmole), 4-nitrobenzenesulfonyl chloride (160.16 mg, 0.728 mmole), DIPEA (112 mg, 0.8736 mmole) in THF (10 mL) at RT for 20 min. The title compound was isolated (Eluent 55% ethyl acetate in Hexane). TLC: Rf = 0.5 (EtOAc/Hexane 6:4) [silica gel, UV and $KMnO_4$ stain]. Brown solid, yield 80%, m.p: 100-102^{0} C. **^{1}H NMR** (400 MHz, DMSO-*d*$_6$): δ ppm 10.85 (s, 1H), 10.48 (s, 1H), 8.04-8.04 (m, 4H), 7.93 (d, *J* = 2.00 Hz, 1H), 7.86-7.85 (m, 2H), 7.58-7.56 (m, 7H), 7.34-7.32 (m, 4H), 7.20 (d, *J* = 8.80 Hz, 2H), 4.00 (q, *J* = 7.20 Hz, 2H), 3.64 (s, 2H), 2.63 (s, 3H), 0.86 (t, *J* = 6.80 Hz, 3H). **^{13}C NMR** (100 MHz, DMSO-*d6*): δ ppm 169.84, 168.12, 152.54, 148.40, 145.29, 144.44, 138.09, 137.98, 136.62, 136.39, 135.62, 135.29, 135.17, 133.12, 131.92, 130.31, 130.22, 129.63, 129.51, 129.06, 128.87, 127.79, 126.77, 125.33, 125.18, 124.47, 121.12, 113.59, 61.49, 43.29, 23.50, 13.87. **DEPT-135**: Positive peaks: - δ ppm 135.18, 133.13, 130.31, 130.22, 129.64, 129.52, 129.06, 128.88, 127.86, 126.78, 125.19, 124.48, 121.12, 113.60, 23.50, 13.87. Negative Peak: - 61.49, 43.29.3.

3. DFT (FMO, MEP) Calculations and Diagrams

Fig S3.1

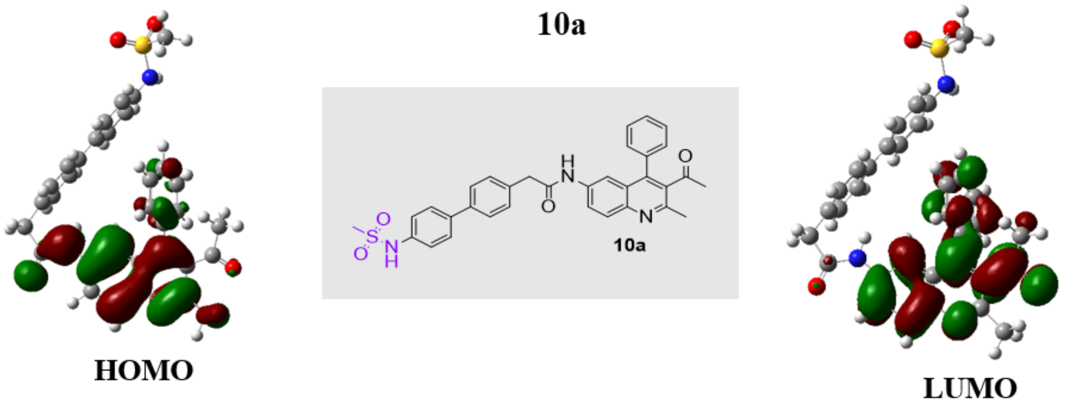

Fig S3.2

10b

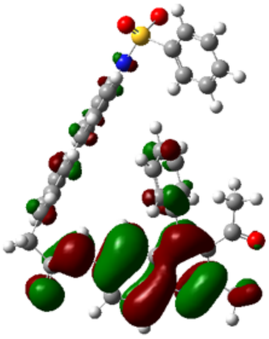

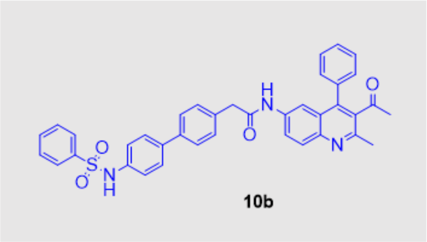

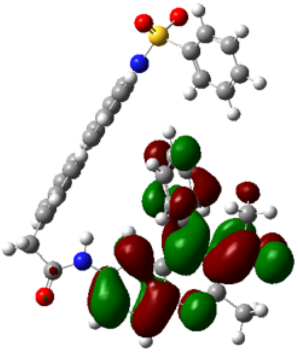

HOMO **LUMO**

Fig S3.3

10c

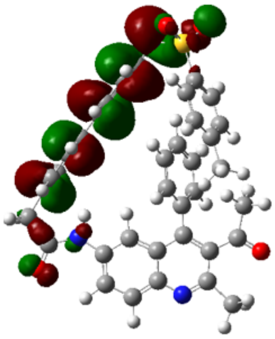

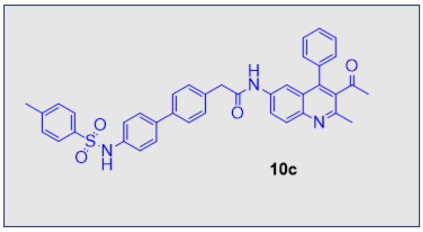

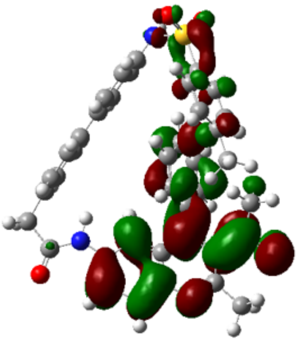

HOMO **LUMO**

Fig S3.4

10d

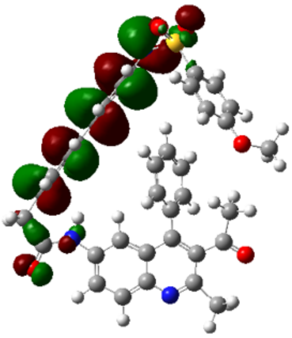

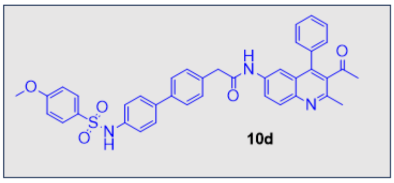

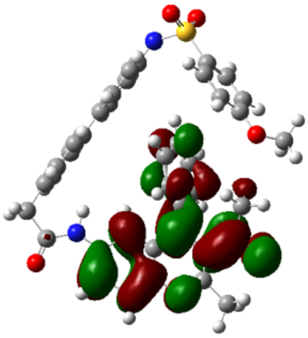

HOMO **LUMO**

Fig S3.5

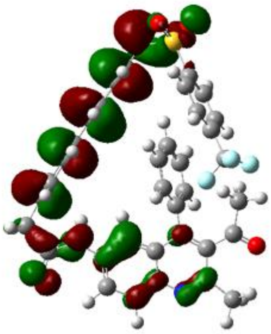

10e

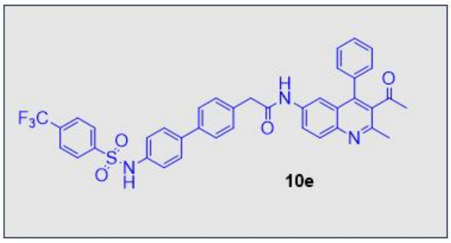

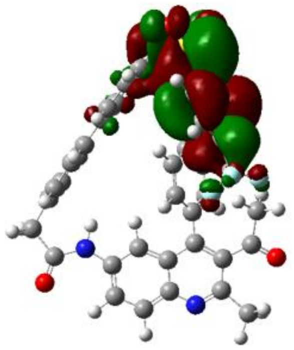

HOMO **LUMO**

Fig S3.6

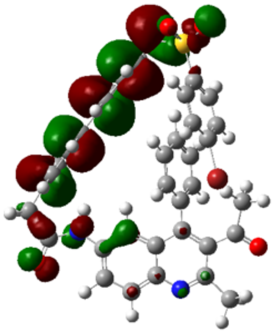

10f

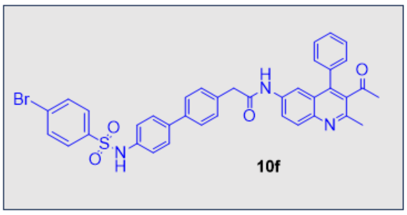

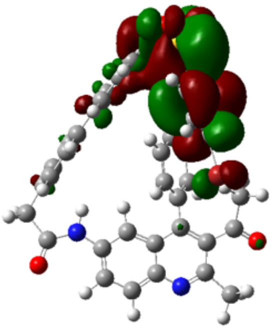

HOMO **LUMO**

Fig S3.7

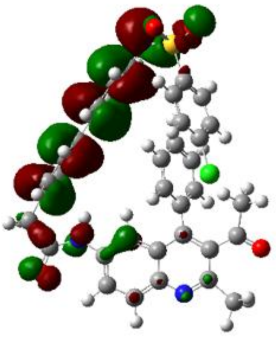

10g

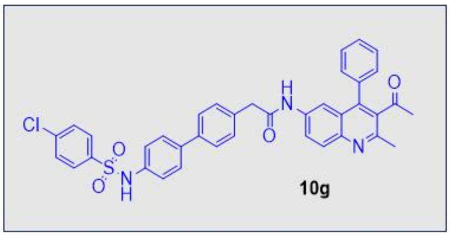

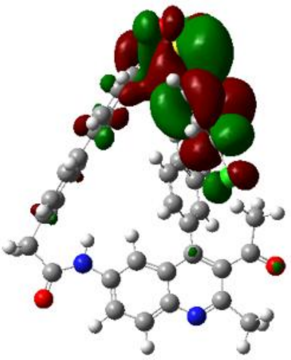

HOMO **LUMO**

Fig S3.8

10h

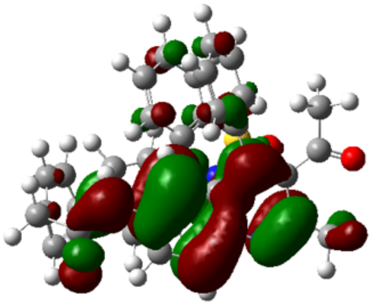

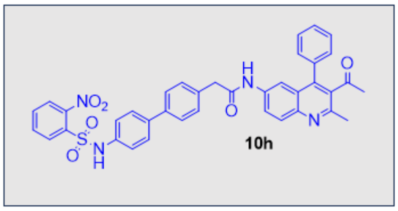

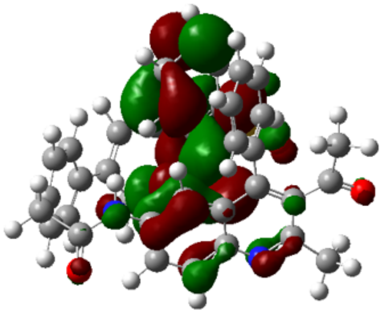

HOMO **LUMO**

Fig S3.9

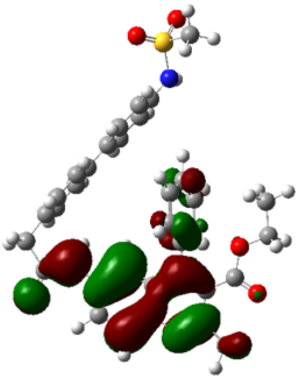

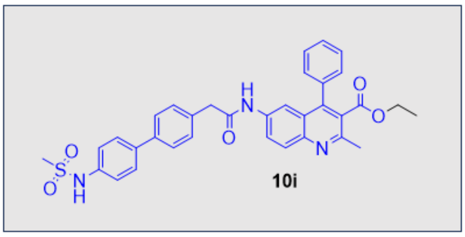

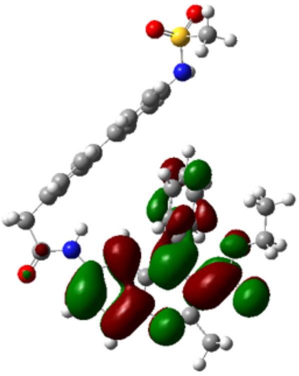

HOMO **LUMO**

Fig S3.10

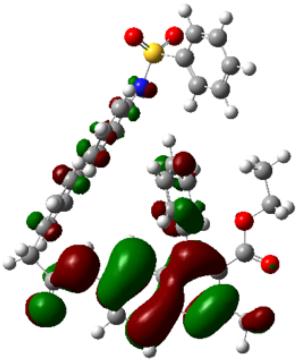

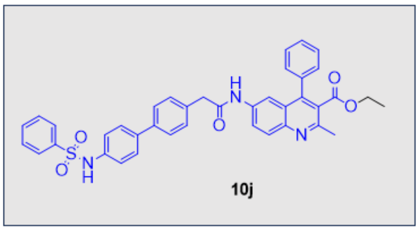

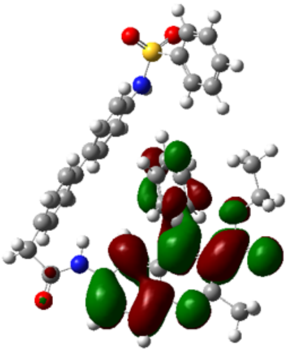

HOMO **LUMO**

Fig S3.11

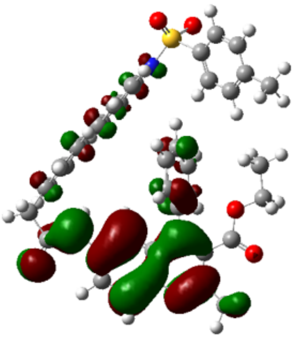

HOMO

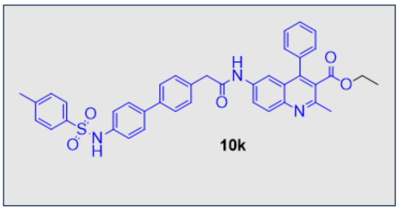

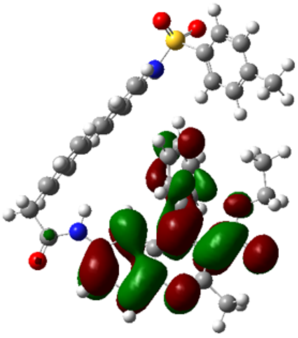

LUMO

Fig S3.12

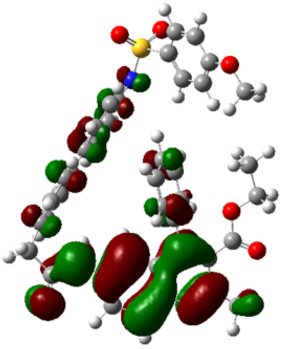

HOMO

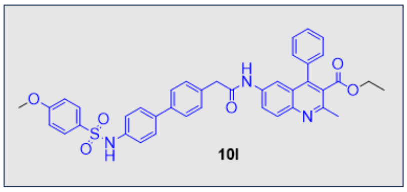

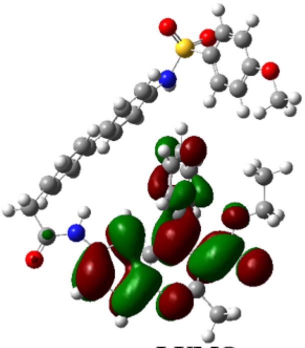

LUMO

Fig S3.13

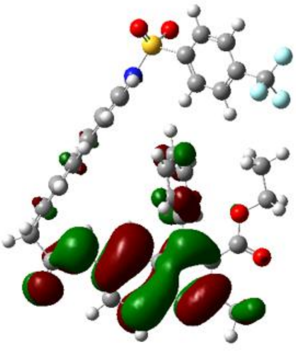

HOMO

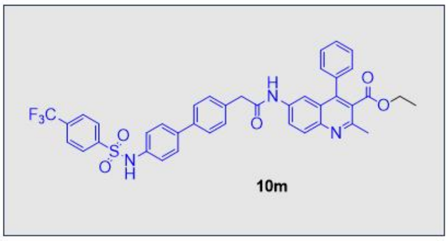

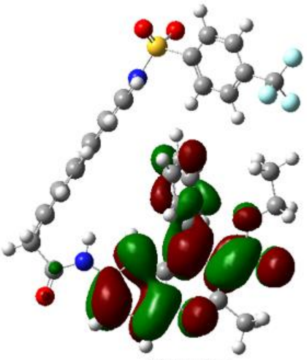

LUMO

Fig S3.14

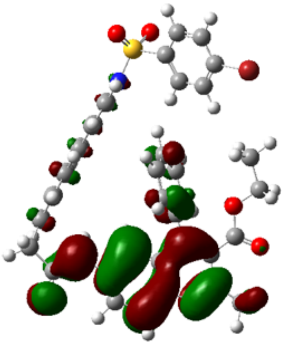

HOMO

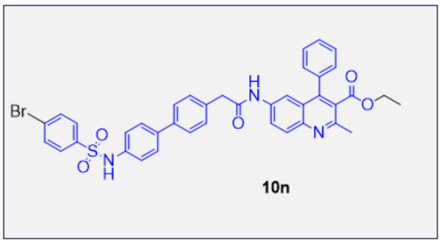

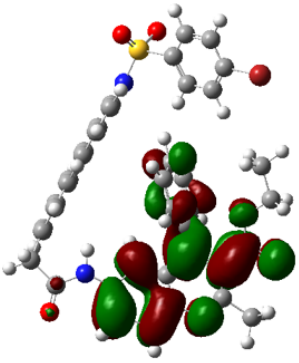

LUMO

Fig S3.15

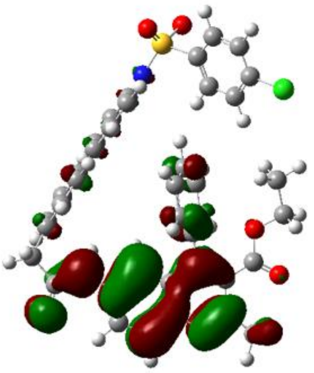

HOMO

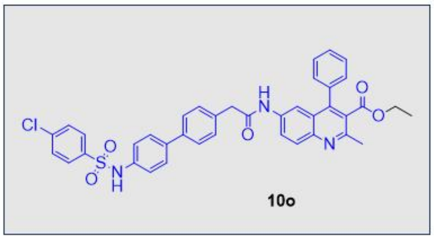

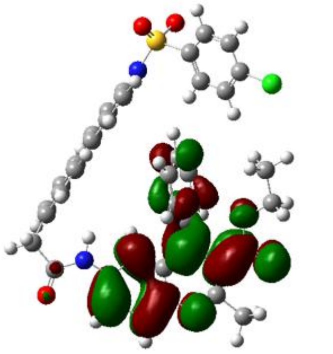

LUMO

Fig S3.16

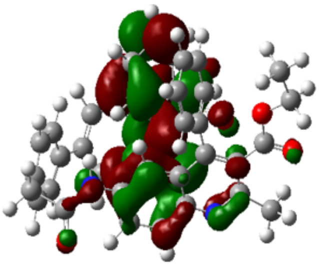

HOMO

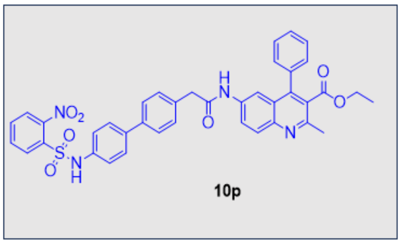

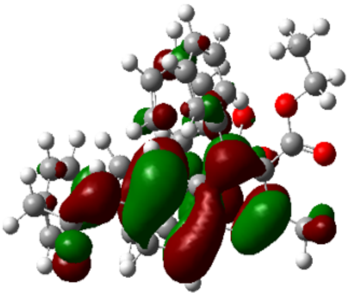

LUMO

Figure. S4 diagram of Molecular electrostatic Potential (10a-10p)

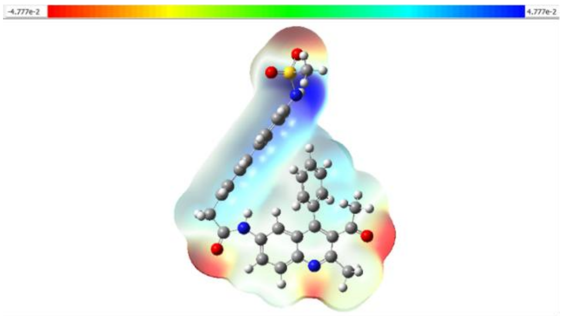

10a

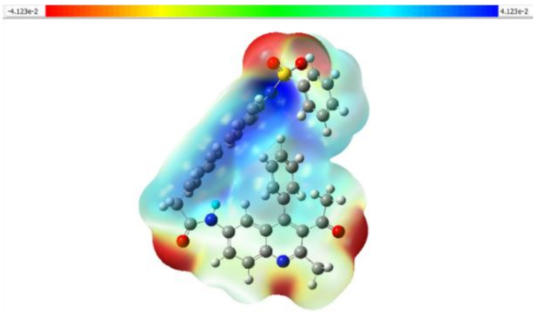

10b

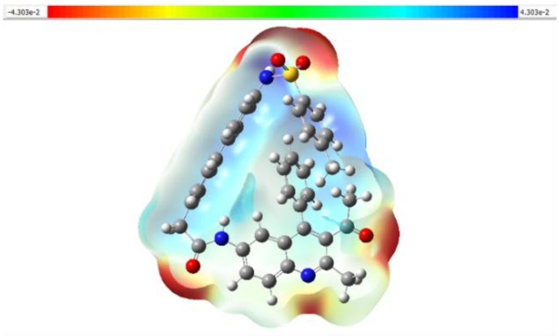

10c

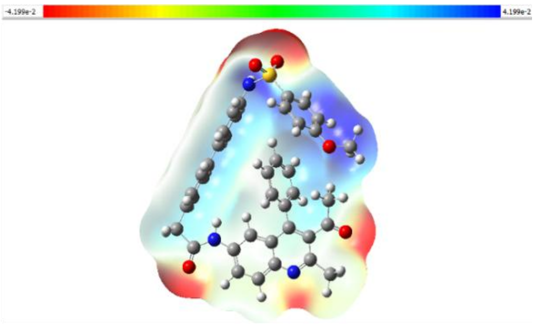

10d

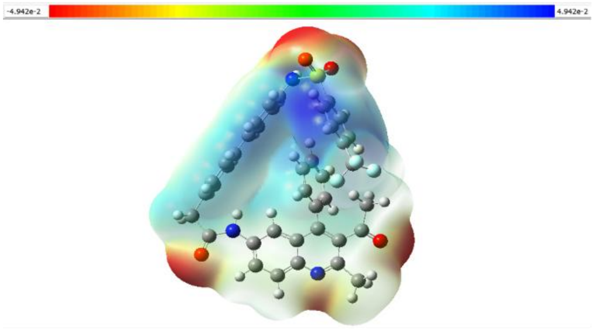

10e

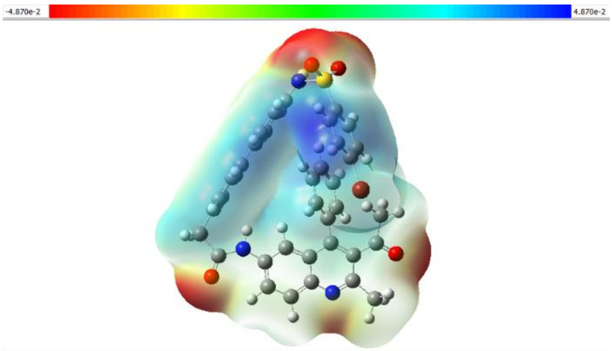

10f

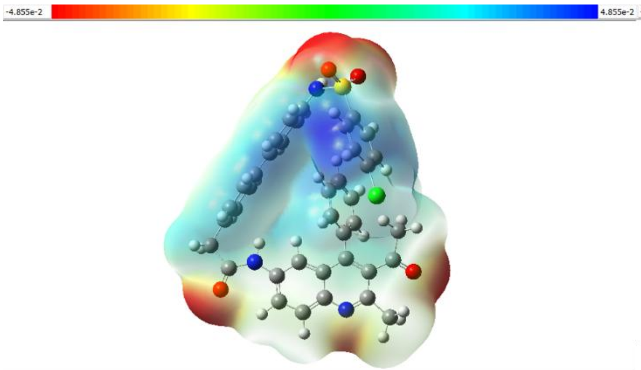

10g

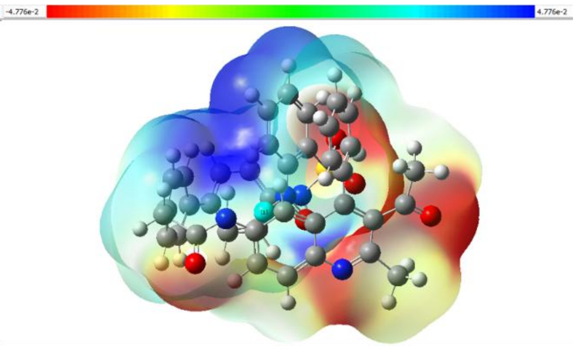

10h

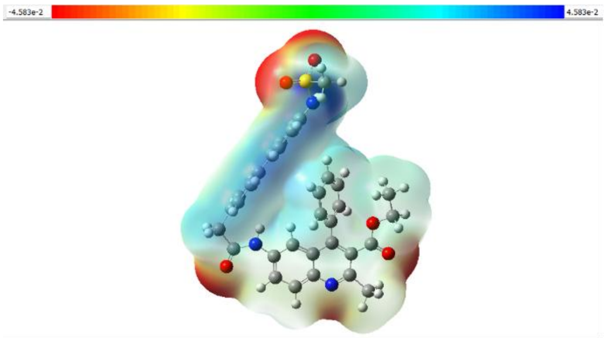

10i

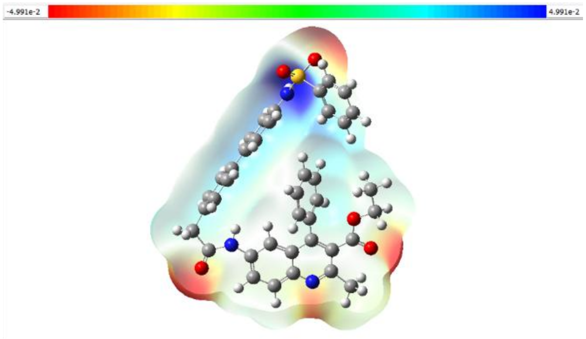

10j

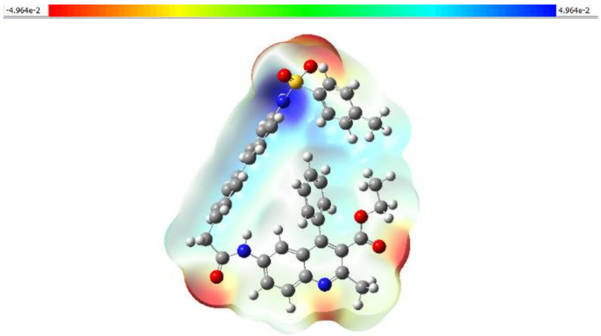

10k

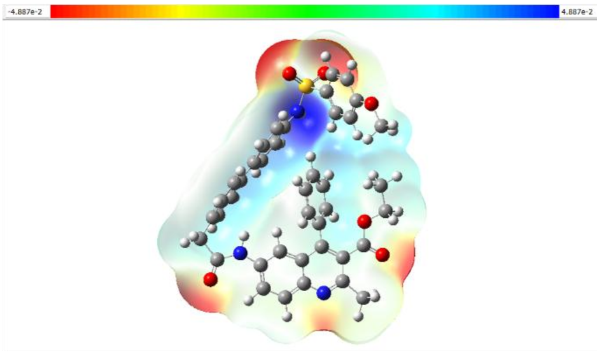

10l

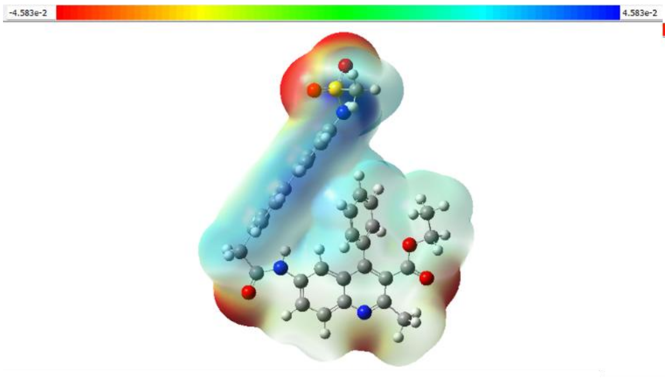

10m

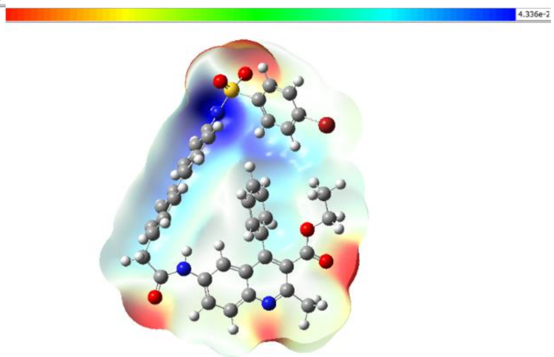

10n

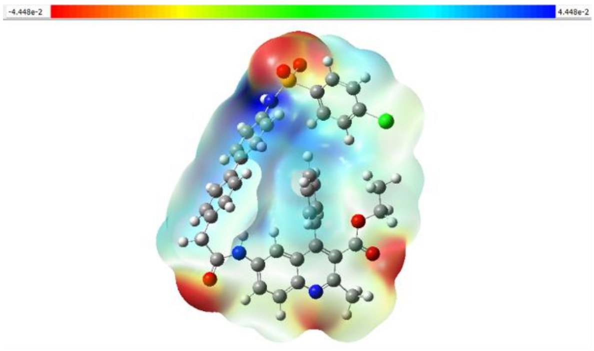

10o

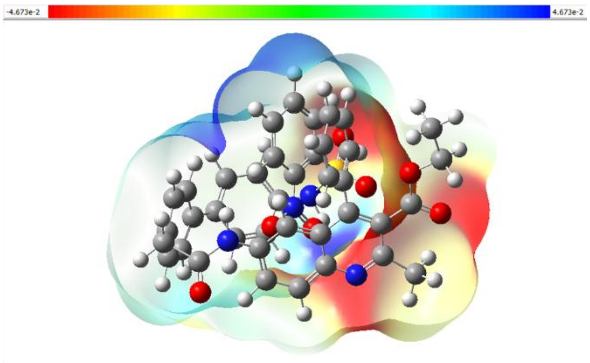

10p

Figure. S5 diagram of TD DFT UV Spectra (10a-10p)

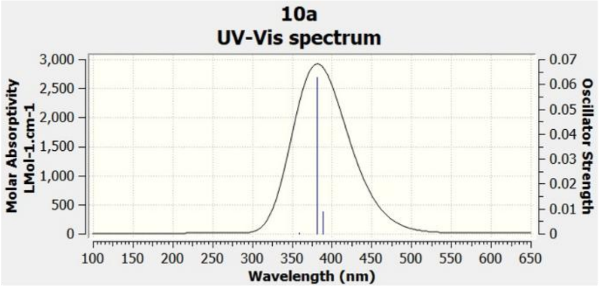

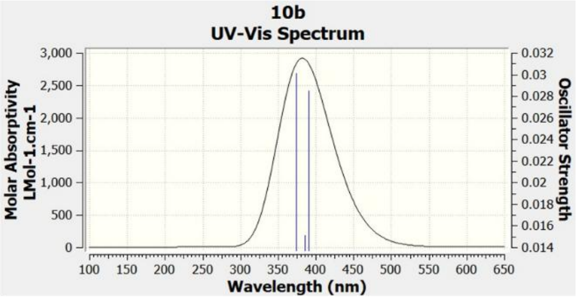

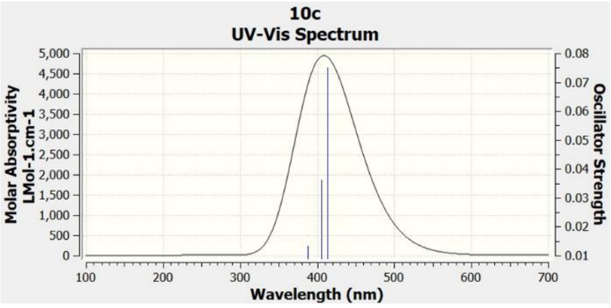

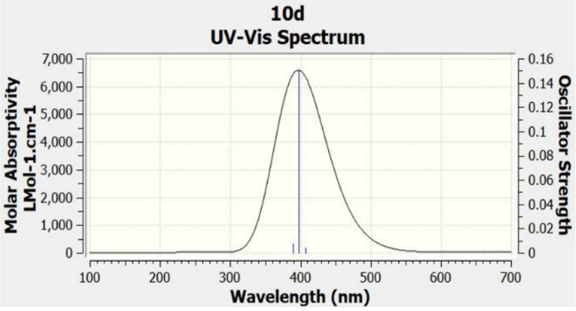

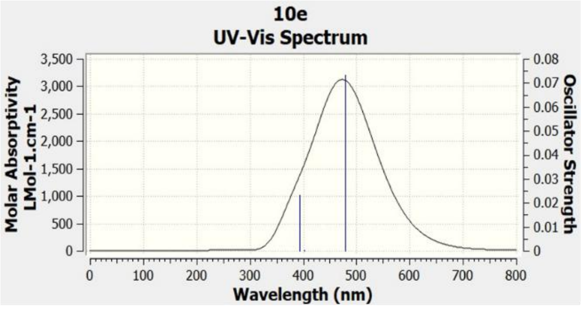
10e
UV-Vis Spectrum
Molar Absorptivity LMol-1.cm-1
Oscillator Strength
Wavelength (nm)

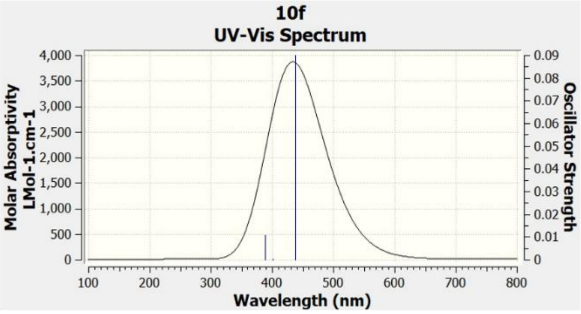
10f
UV-Vis Spectrum
Molar Absorptivity LMol-1.cm-1
Oscillator Strength
Wavelength (nm)

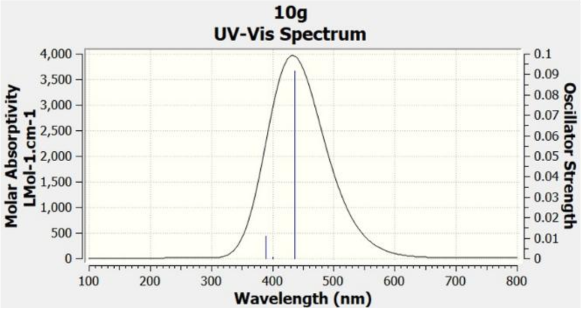
10g
UV-Vis Spectrum
Molar Absorptivity LMol-1.cm-1
Oscillator Strength
Wavelength (nm)

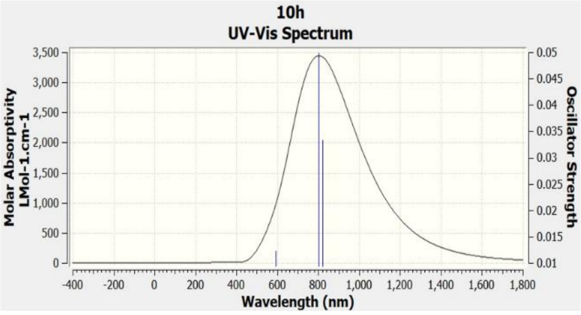
10h
UV-Vis Spectrum
Molar Absorptivity LMol-1.cm-1
Oscillator Strength
Wavelength (nm)

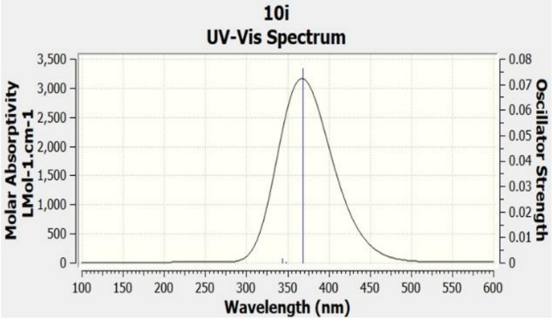
10i
UV-Vis Spectrum
Molar Absorptivity LMol-1.cm-1
Oscillator Strength
Wavelength (nm)

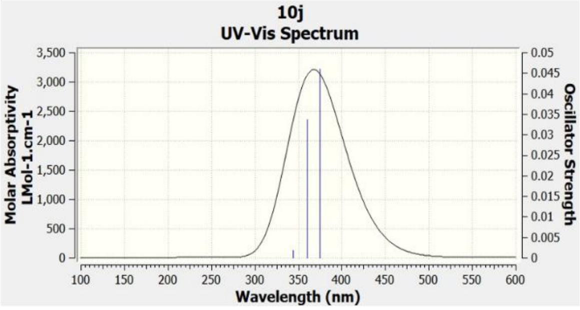
10j
UV-Vis Spectrum
Molar Absorptivity LMol-1.cm-1
Oscillator Strength
Wavelength (nm)

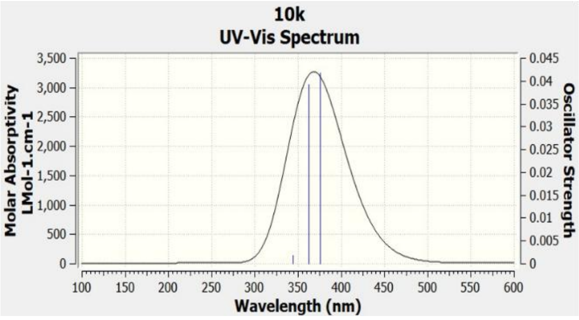
10k
UV-Vis Spectrum
Molar Absorptivity LMol-1.cm-1
Oscillator Strength
Wavelength (nm)

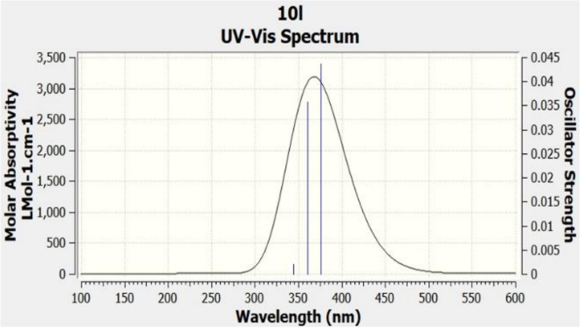
10l
UV-Vis Spectrum
Molar Absorptivity LMol-1.cm-1
Oscillator Strength
Wavelength (nm)

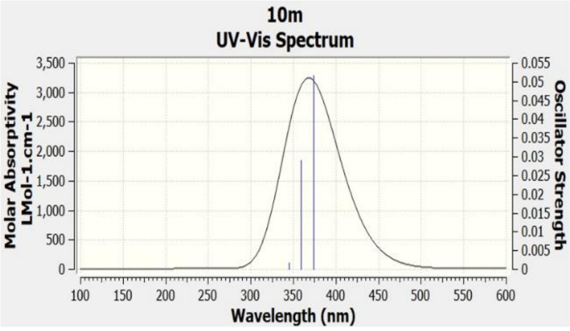

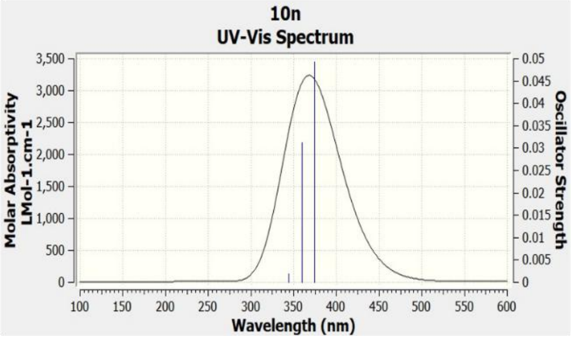

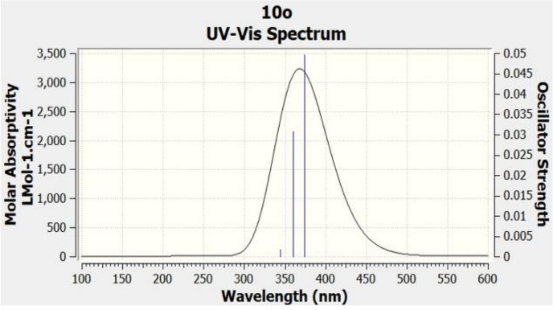

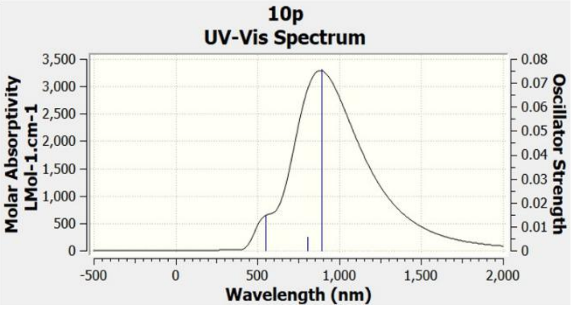

Table S1. Selected Major contribution transition states of compounds (10a-10p).

Entry	Experimental λAbs	Theoretical λAbs	Oscillation Strength, f	Energy, ΔE	Selected Major contributions (%)
10a	339.63	388.53<br>381.27<br>358.96	0.0090<br>0.0628<br>0.0004	3.1911<br>3.2518<br>3.4540	H+2→L (52.99)<br>H→L (78.96)<br>H+1→L (88.86)
10b	340.00	390.09<br>385.61<br>373.69	0.0285<br>0.0151<br>0.0301	3.1784<br>3.2153<br>3.3179	H→L (46.68)<br>H→L (35.09)<br>H→L+1 77.96
10c	341.21	413.58<br>405.69<br>388.21	0.0750<br>0.0362<br>0.0133	2.9979<br>3.0561<br>3.1937	H→L-1 (52.53)<br>H→L (53.56)<br>H+2→L (48.39)
10d	340.04	406.83<br>397.25<br>388.97	0.0042<br>0.1508<br>0.0074	3.0475<br>3.1211<br>3.1875	H→L (99.12)<br>H→L-1 (97.56)<br>H+2→L (56.73)
10e	338.41	478.79<br>401.98<br>393.12	0.0734<br>0.0004<br>0.0231	2.5895<br>3.0843<br>3.1538	H→L (97.77)<br>H→L-1 (98.42)<br>H+1→L (83.83)
10f	341.55	437.48<br>401.33<br>389.07	0.0899<br>0.0004<br>0.0110	2.8340<br>3.0893<br>3.1867	H→L (54.40)<br>H→L-1 (54.58)<br>H+2→L-1 (29.84)
10g	340.39	436.60<br>400.99<br>388.92	0.0917<br>0.0007<br>0.0112	2.8397<br>3.0920<br>3.1879	H→L (54.11)<br>H→L-1 (54.31)<br>H+2→L (30.86)
10h	337.83	820.71<br>803.45<br>592.81	0.0333<br>0.0499<br>0.0122	1.5107<br>1.5431<br>2.0915	H→L (60.15)<br>H+2→L (61.43)<br>H+6→L (36.81)

10i	340.22	368.13<br>347.69<br>343.63	0.0763<br>0.0005<br>0.0017	3.3680<br>3.5659<br>3.6080	H→L (94.54)<br>H+1→L (94.28)<br>H+2→L (95.69)
10j	341.73	374.42<br>360.43<br>344.17	0.0459<br>0.0337<br>0.0019	3.3113<br>3.4399<br>3.6025	H→L (81.21)<br>H+1→L (79.91)<br>H+2→L (98.15)
10k	338.99	375.26<br>362.28<br>344.43	0.0417<br>0.0393<br>0.0018	3.3040<br>3.4223<br>3.5997	H→L (80.29)<br>H+1→L (78.84)<br>H+2→L (98.31)
10l	340.80	375.90<br>360.69<br>344.19	0.0436<br>0.0358<br>0.0020	3.2983<br>3.4374<br>3.6022	H→L (80.29)<br>H+1→L (78.84)<br>H+2→L (98.31)
10m	340.22	374.17<br>359.44<br>344.65	0.0517<br>0.0291<br>0.0017	3.3136<br>3.4494<br>3.5974	H→H\L (79.25)<br>H+1→L (78.40)<br>H+2→L (98.27)
10n	338.41	374.57<br>360.10<br>344.71	0.0493<br>0.0312<br>0.0018	3.3101<br>3.4430<br>3.5968	H→L (79.65)<br>H+1→L (78.63)<br>H+2→L (98.24)
10o	340.11	374.27<br>360.03<br>344.60	0.0497<br>0.0307<br>0.0017	3.3127<br>3.4438<br>3.5980	H→L (78.85)<br>H+1→L (77.87)<br>H+2→L (98.29)
10p	339.98	890.59<br>807.52<br>548.11	0.0759<br>0.0056<br>0.0148	1.3922<br>1.5354<br>2.2620	H→L (95.98)<br>H+1→L (99.47)<br>H+4→L (40.12)

5. ^{1}H, ^{13}C, DEPT-135 and 2D NMR and HRMS Spectra.

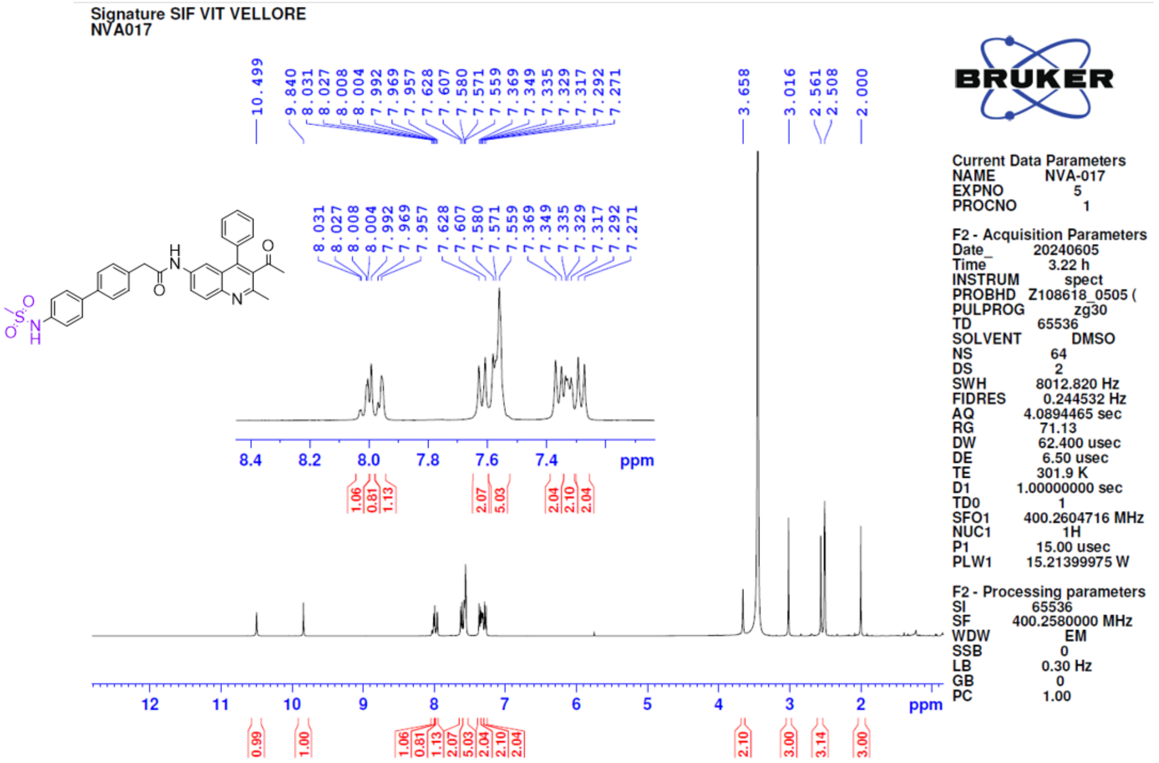

Fig. S6 ^{1}H NMR spectrum of compound-10a

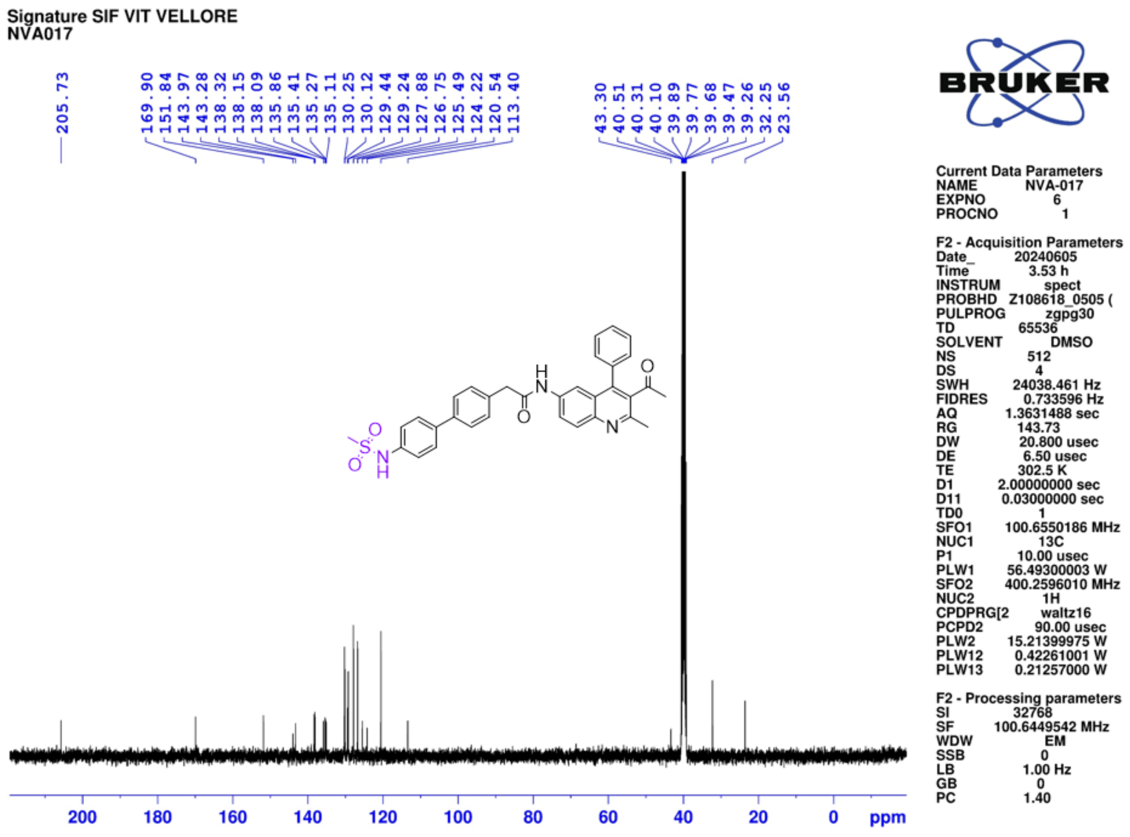

Fig. S7 ^{13}C NMR spectrum of compound-10a

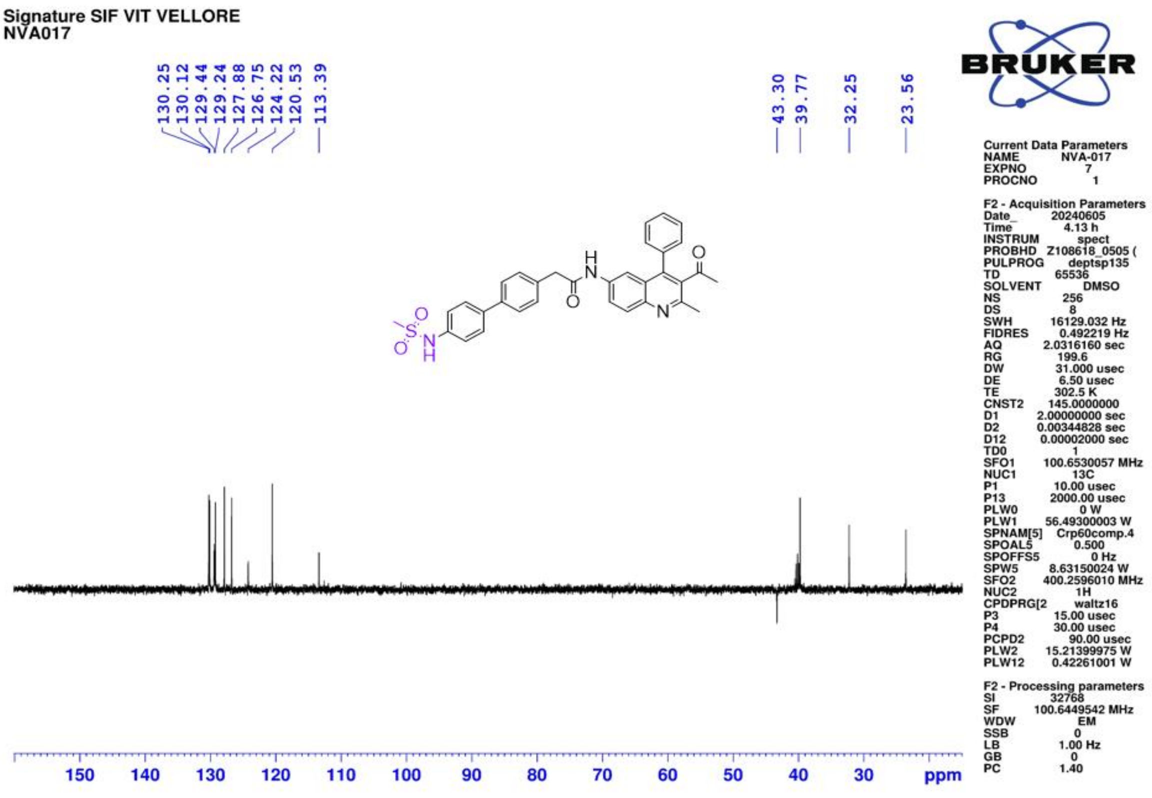

Fig. S8 DEPT-135 NMR spectrum of compound-10a

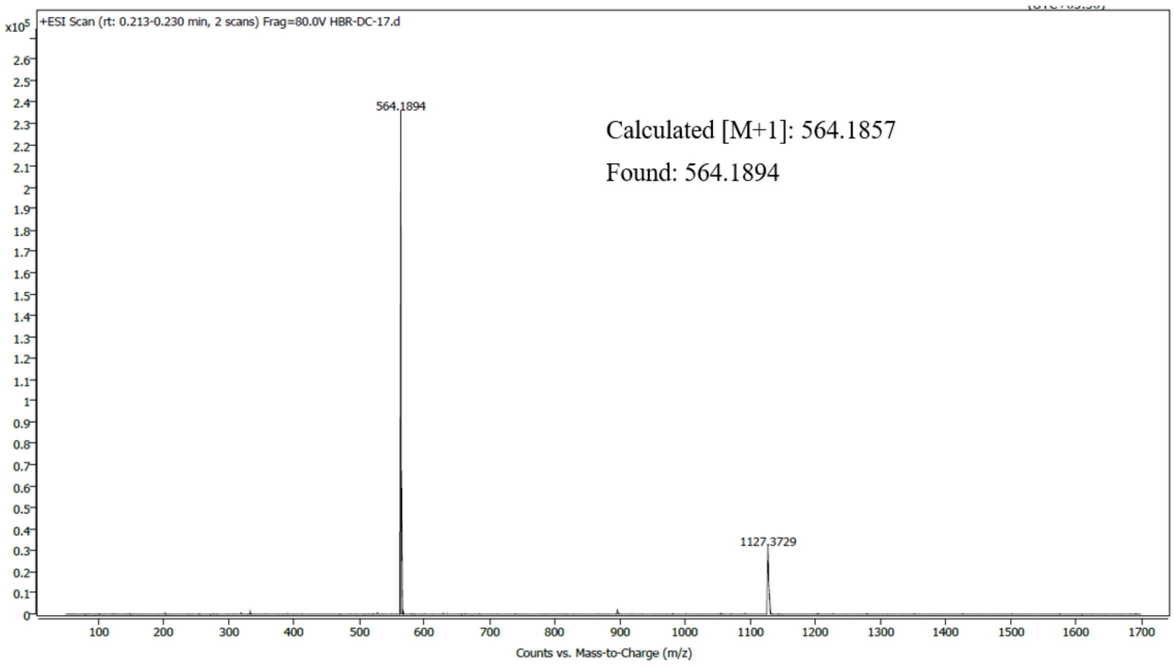

Fig. S9 HRMS of compound-10a

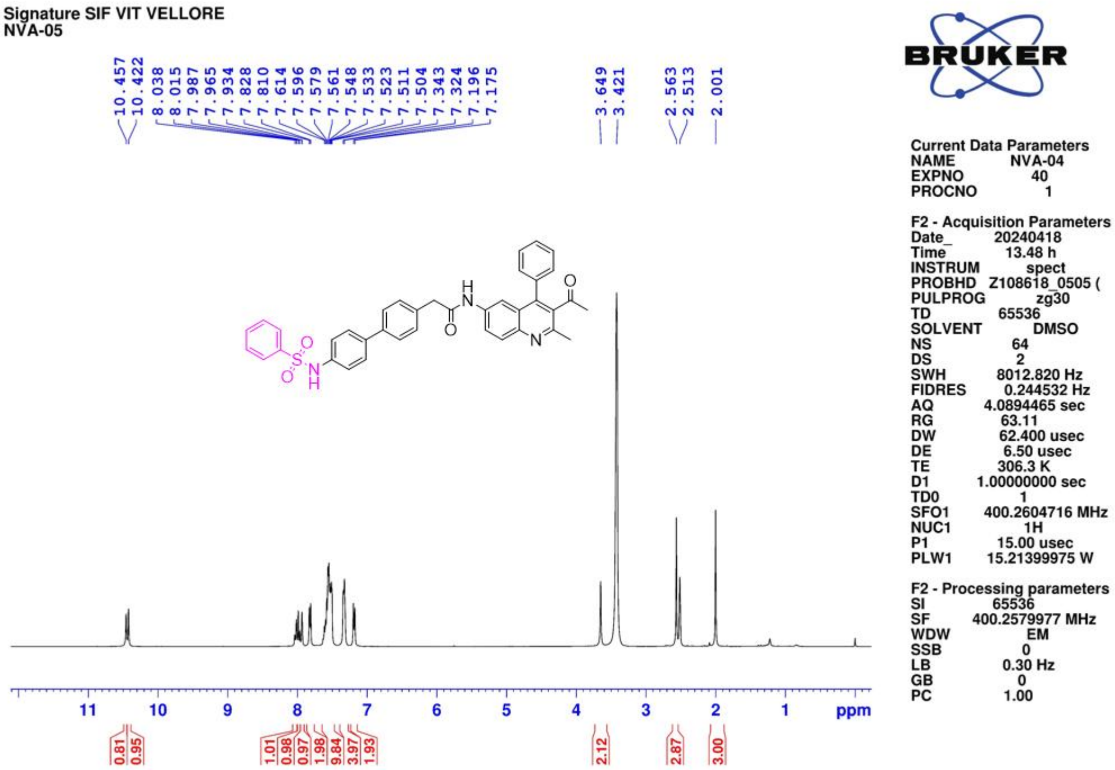

Fig. S10 ^{1}H NMR spectrum of compound 10b

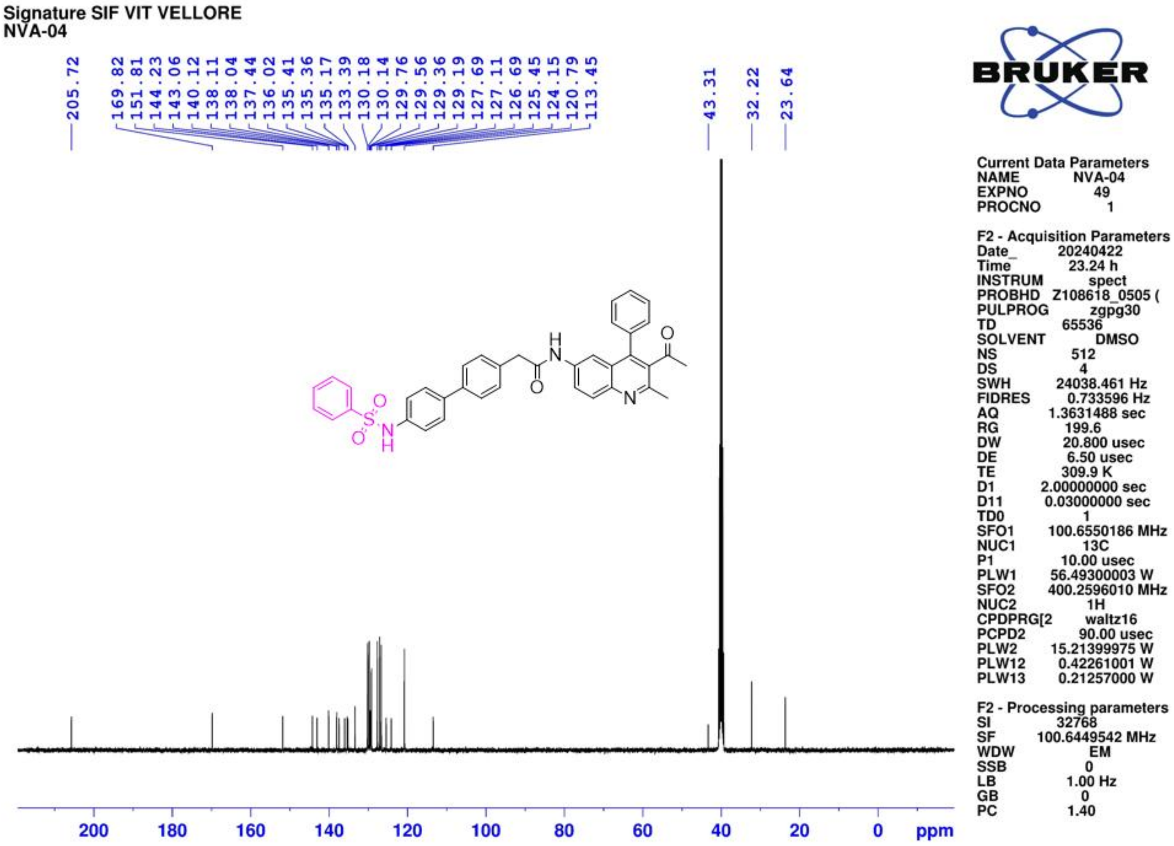

Fig. S11 ^{13}C NMR spectrum of compound 10b

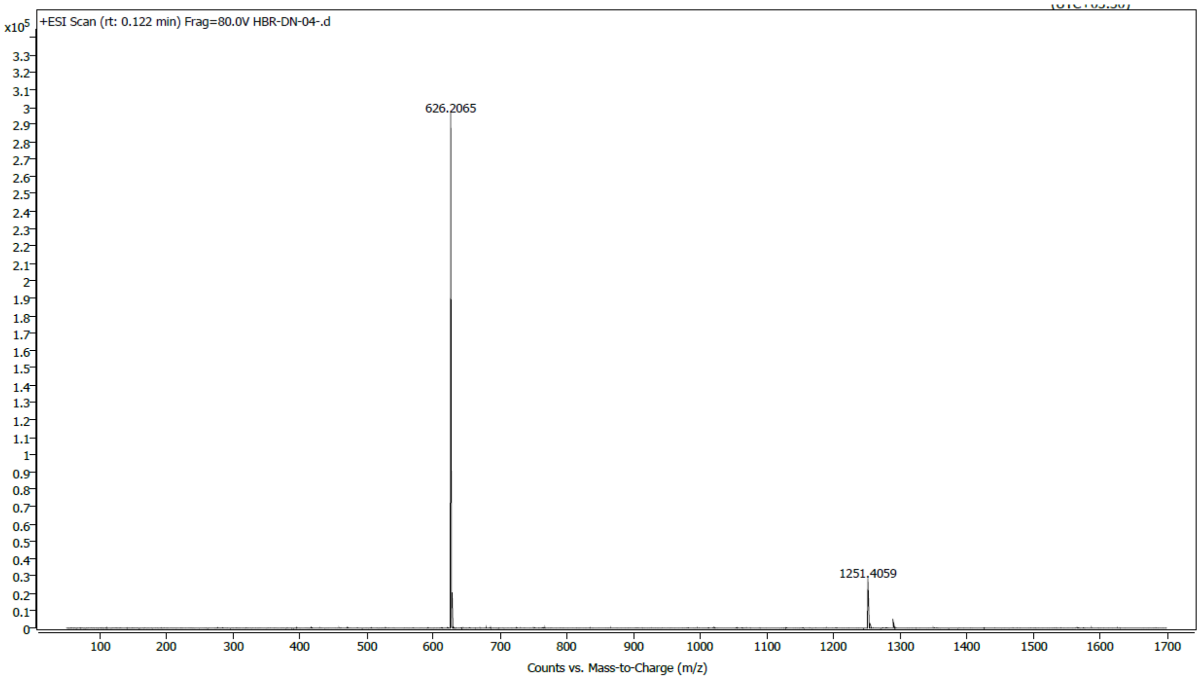

Fig. S12 HRMS of compound 10b

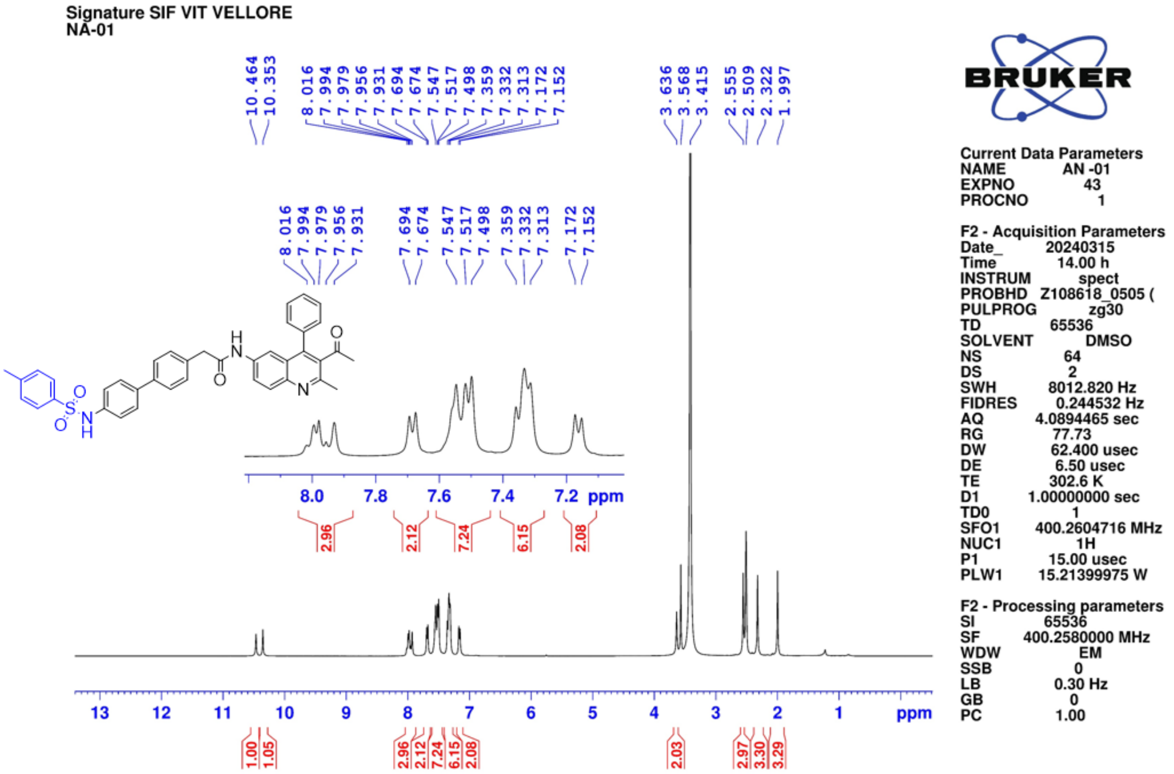

Fig. S13 ^{1}H NMR spectrum of compound 10c

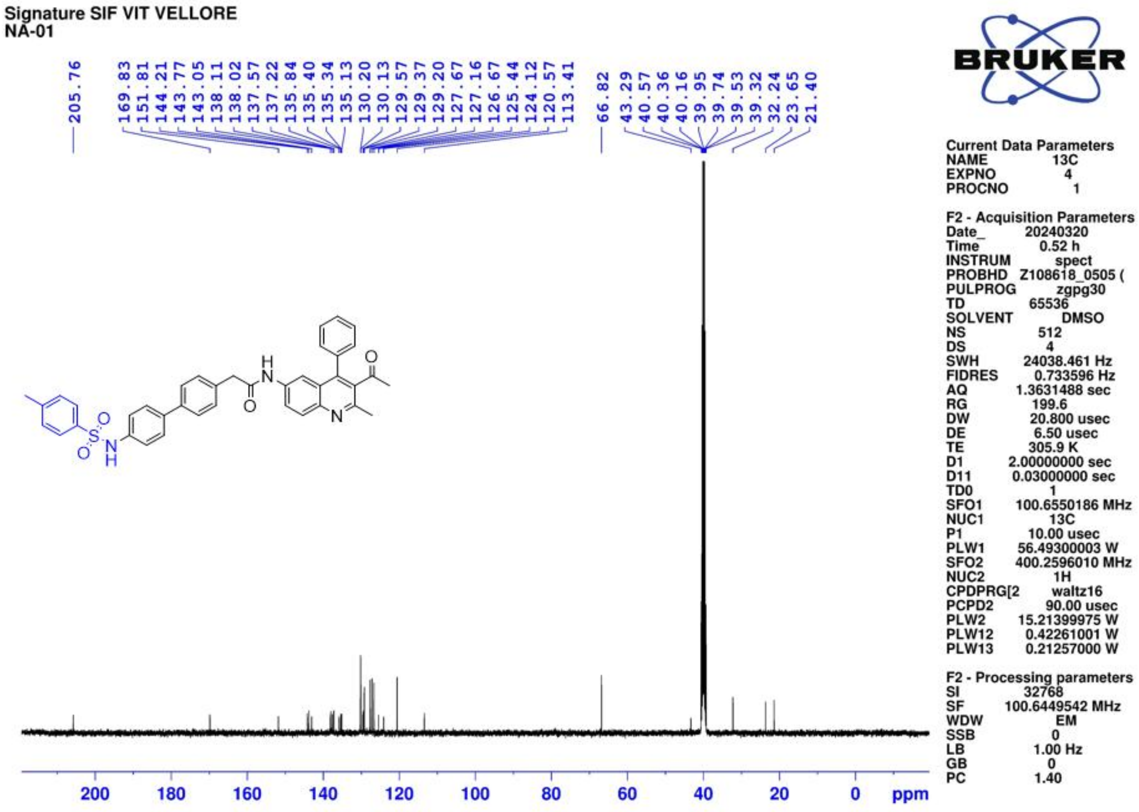

Fig. S14 ^{13}C NMR spectrum of compound 10c

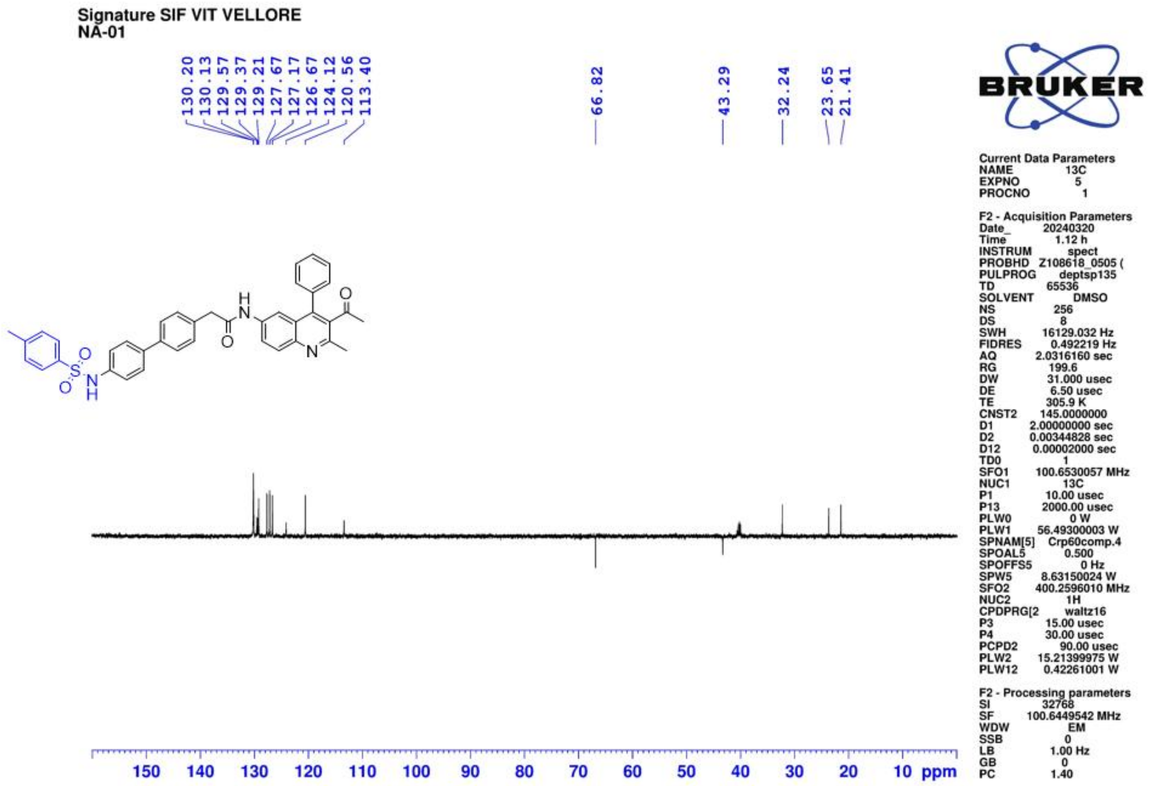

Fig. S15 DEPT-135 NMR spectrum of compound 10c

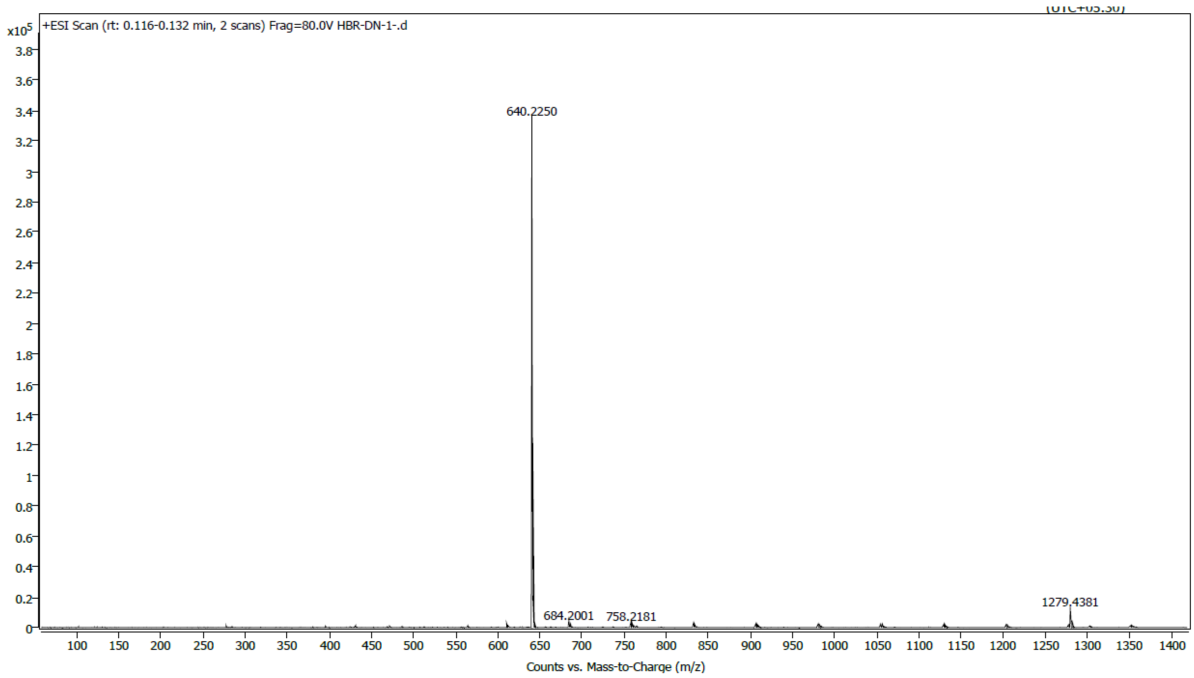

Fig. S16 HRMS of compound 10c

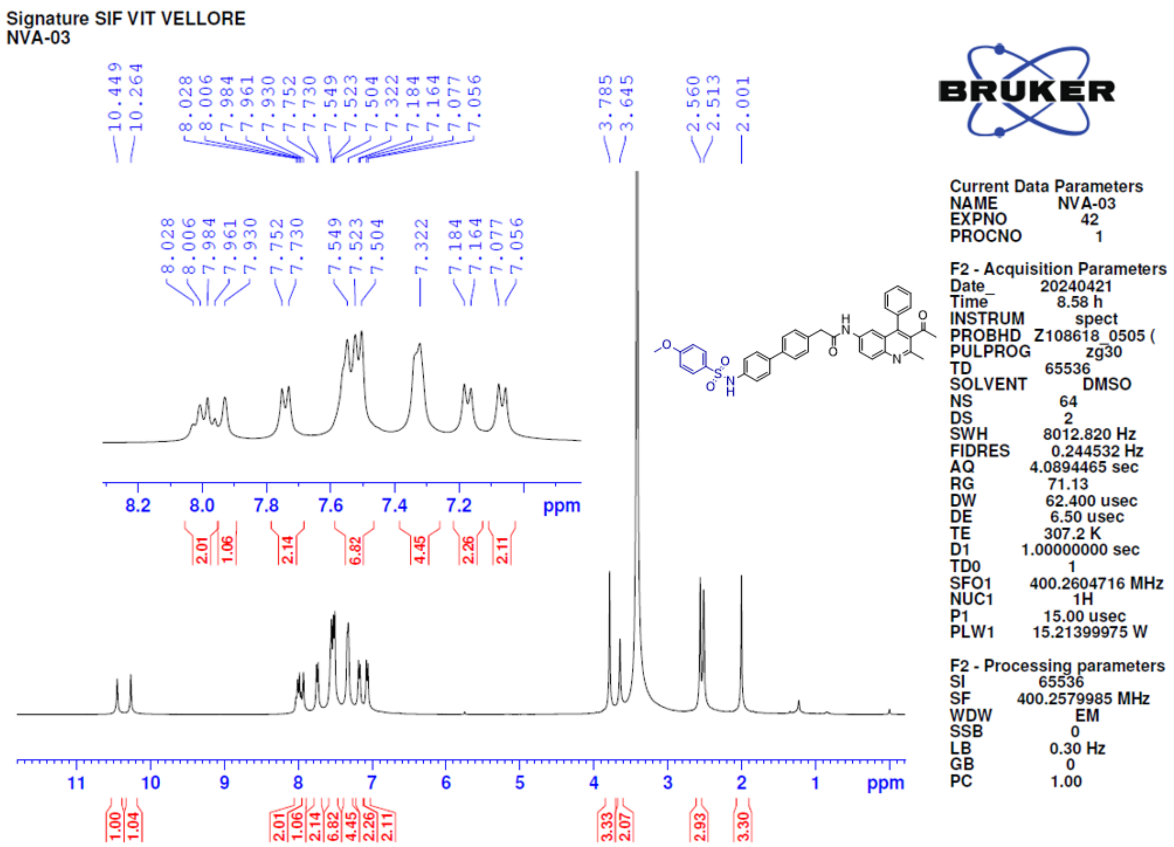

Fig. S17 ^{1}H NMR spectrum of compound 10d

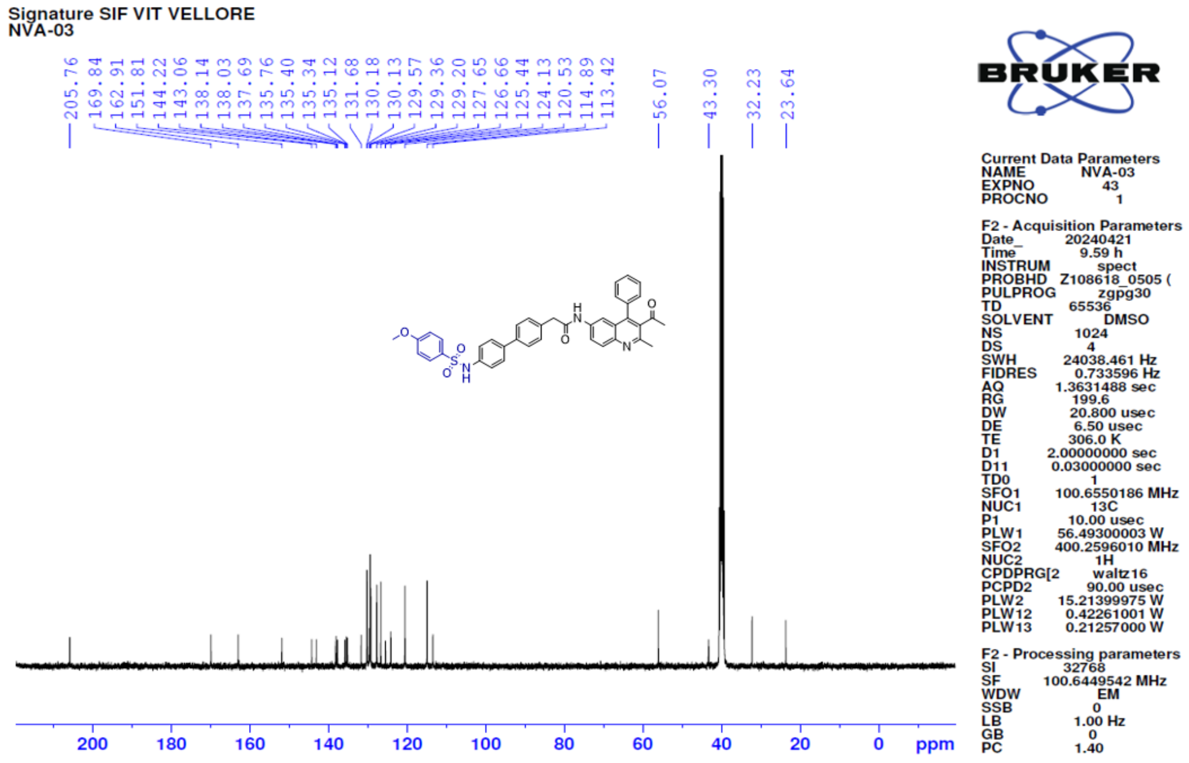

Fig. S18 ^{13}C NMR spectrum of compound 10d

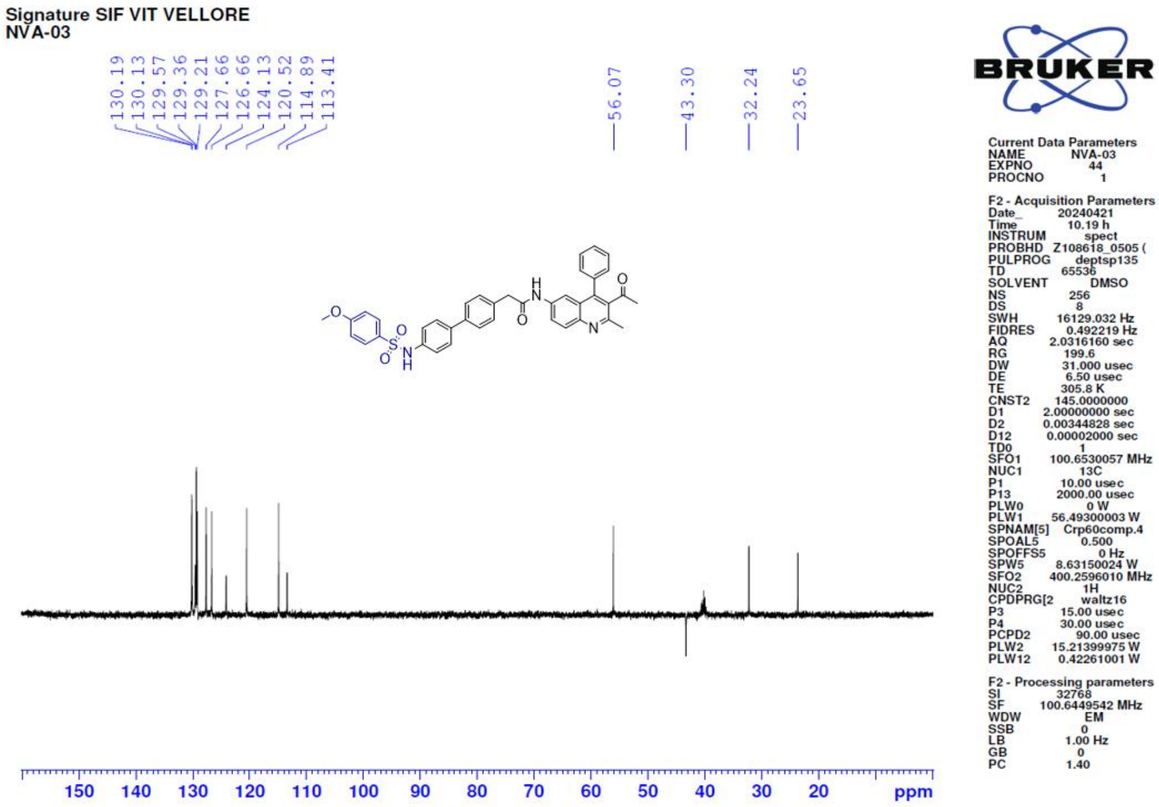

Fig. S19 DEPT-135 spectrum of compound 10d

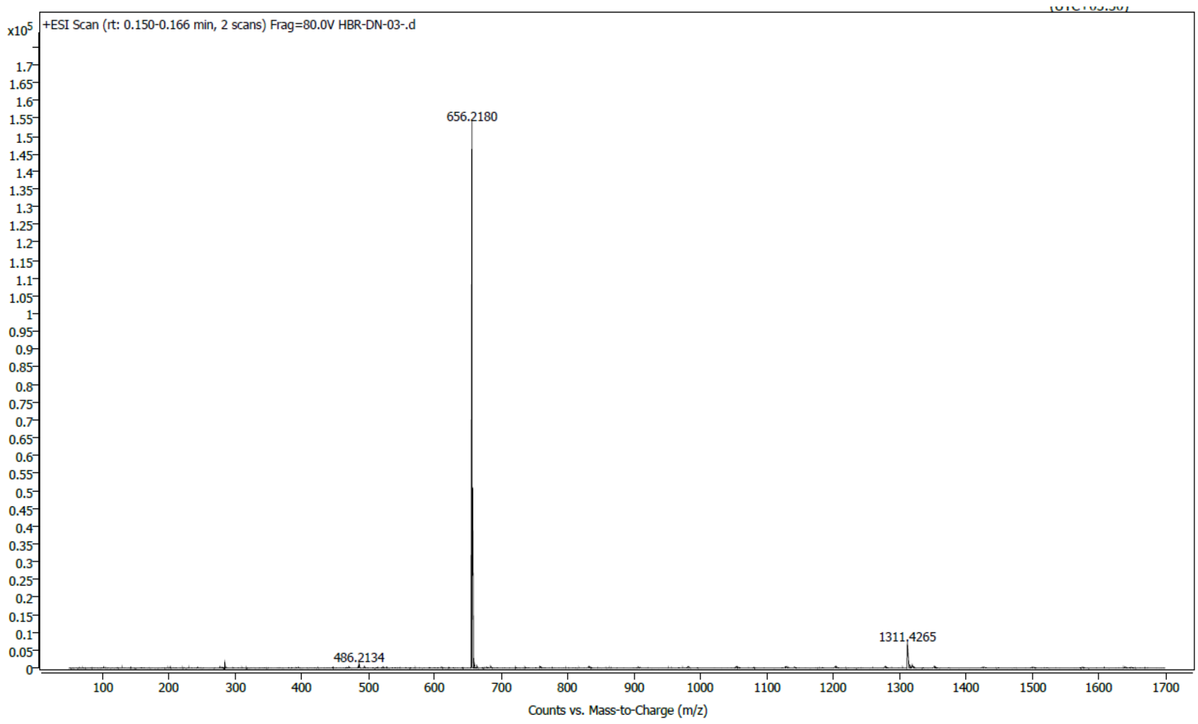

Fig. S20 HRMS of compound 10d

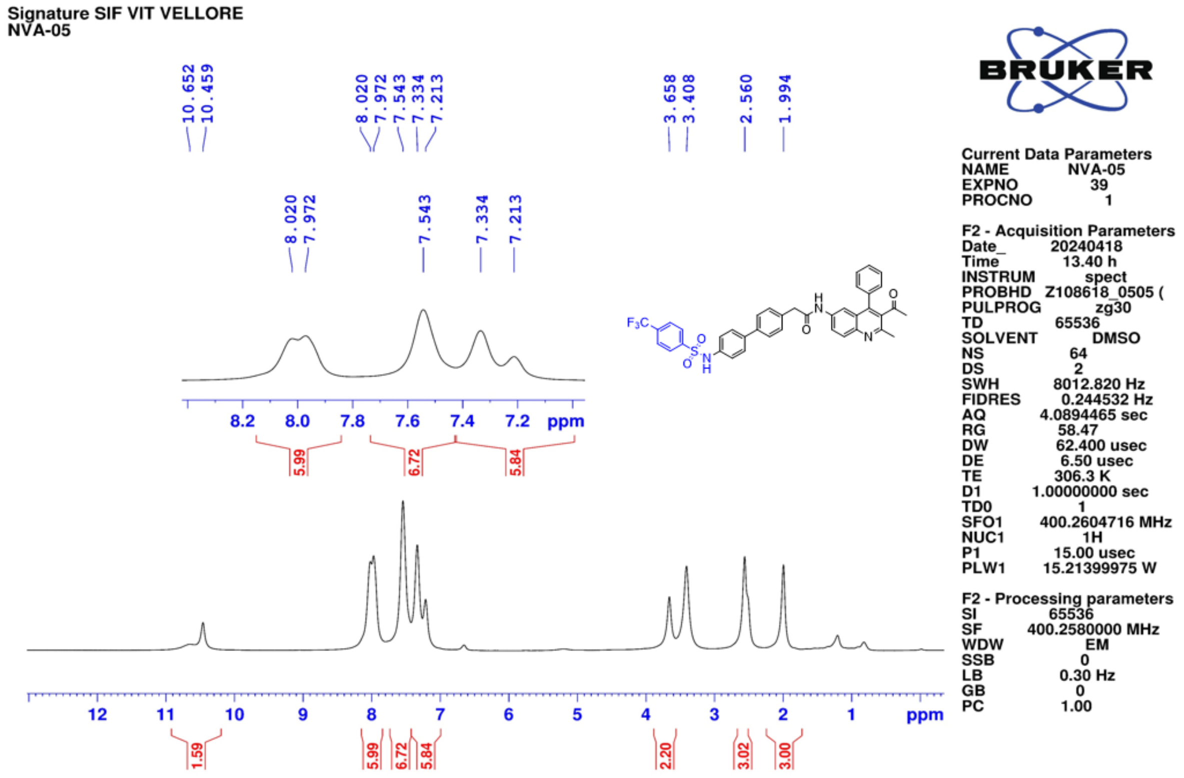

Fig. S21 ^{1}H NMR spectrum of compound 10e

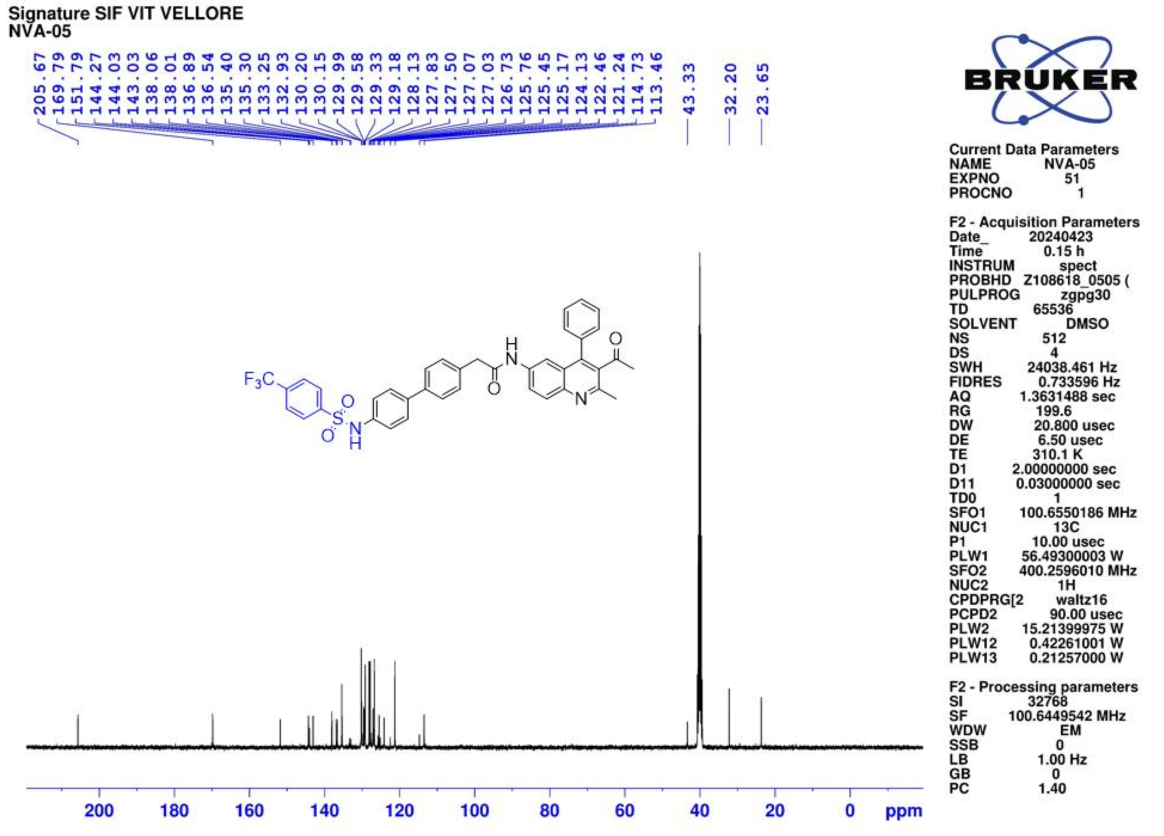

Fig. S22 ^{1}3C NMR spectrum of compound 10e

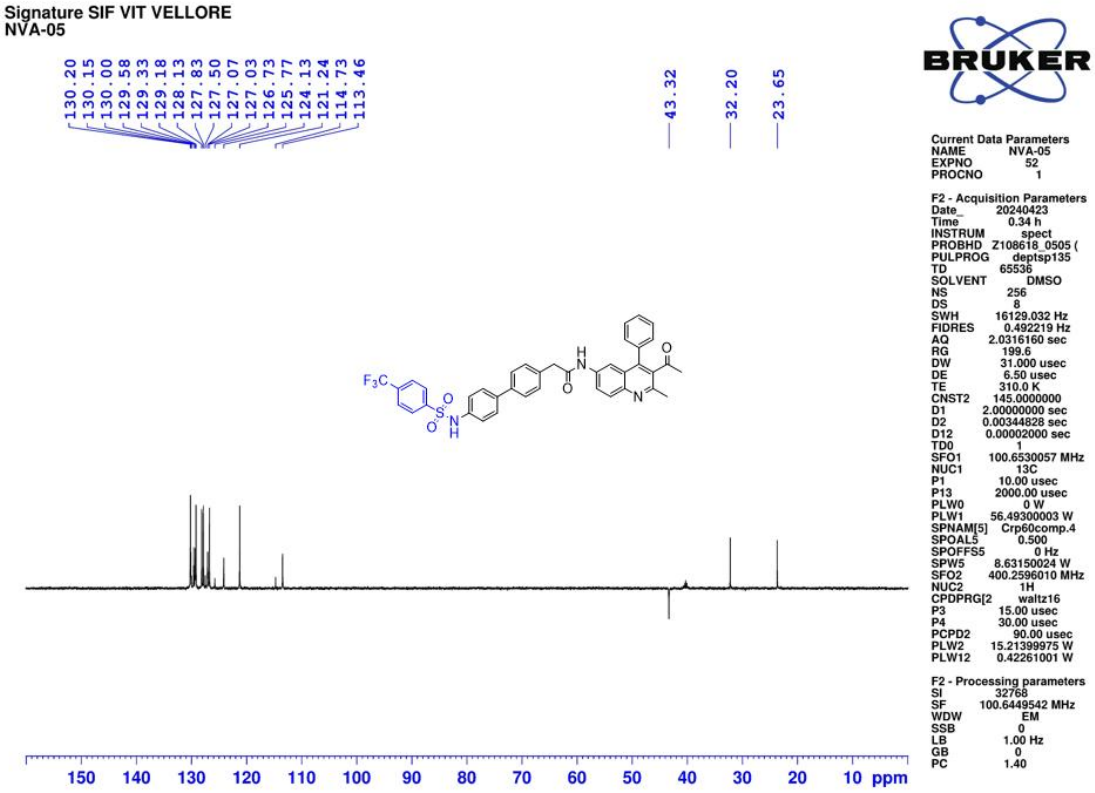

Fig. S23 DEPT-135 NMR spectrum of compound 10e

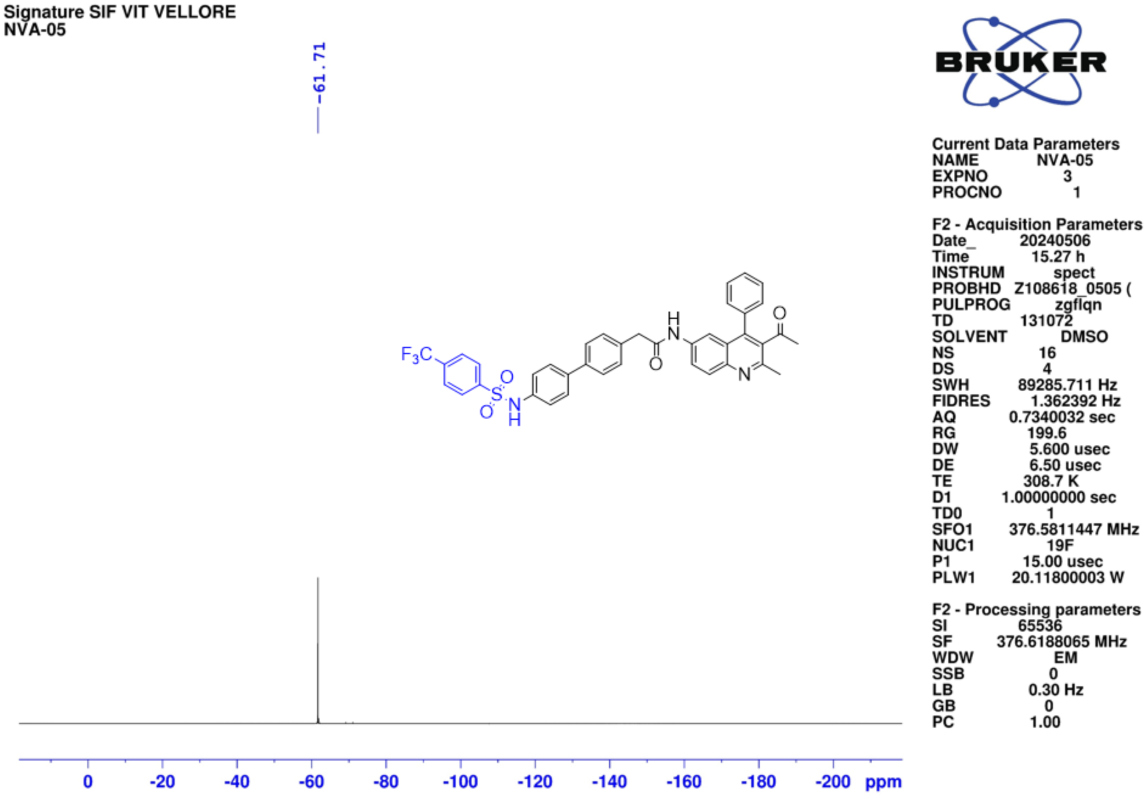

Fig. S24 ^{19}F NMR spectrum of compound 10e

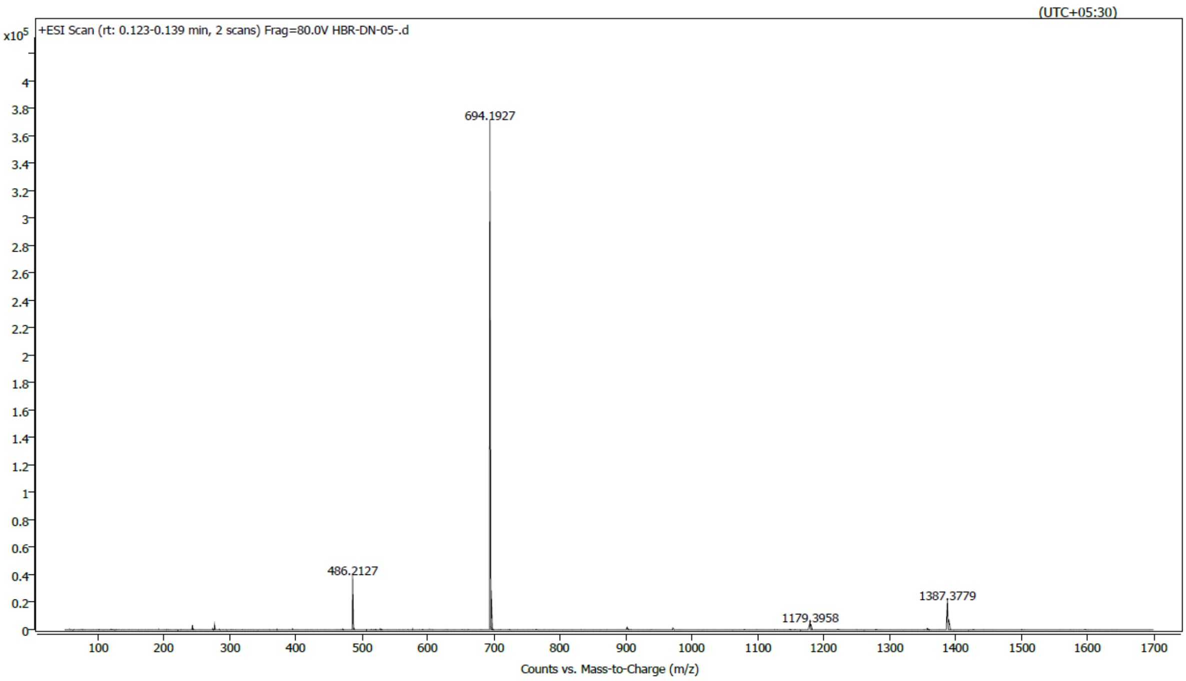

Fig. S25 HRMS of compound 10e.

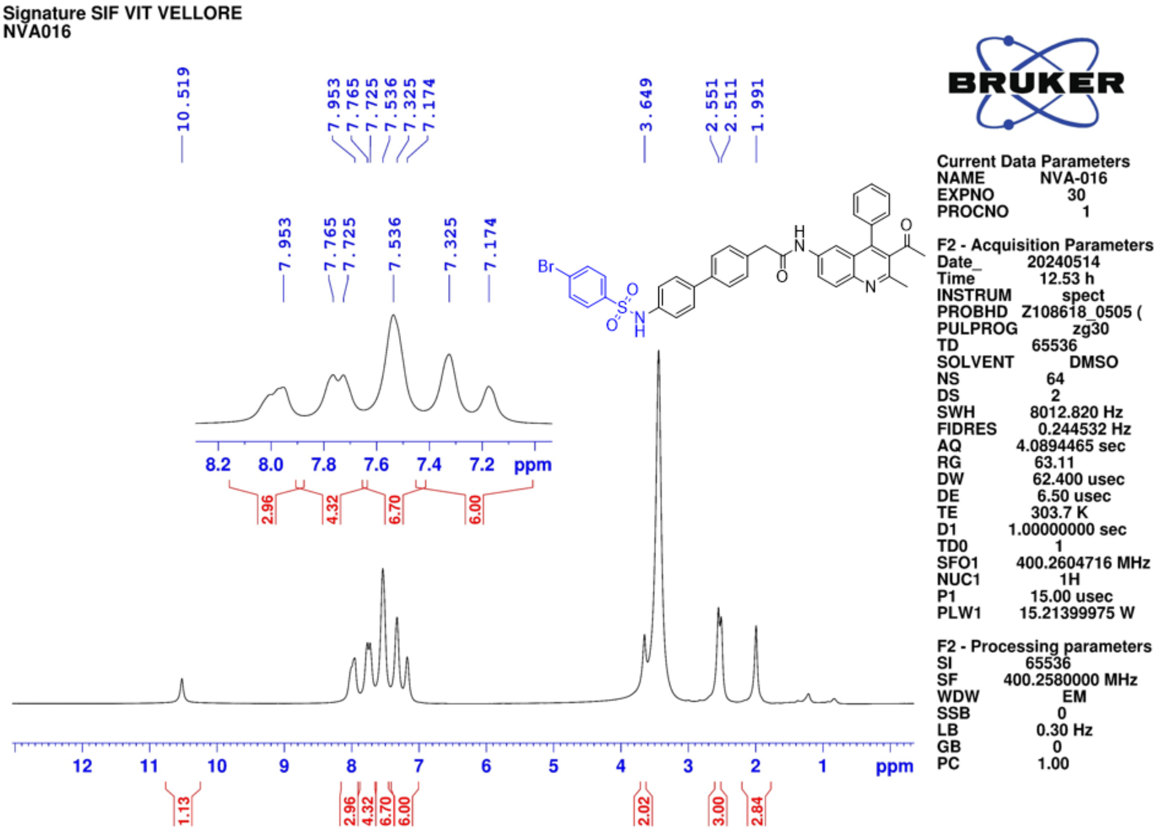

Fig. S26 ^{1}H NMR spectrum of compound 10f

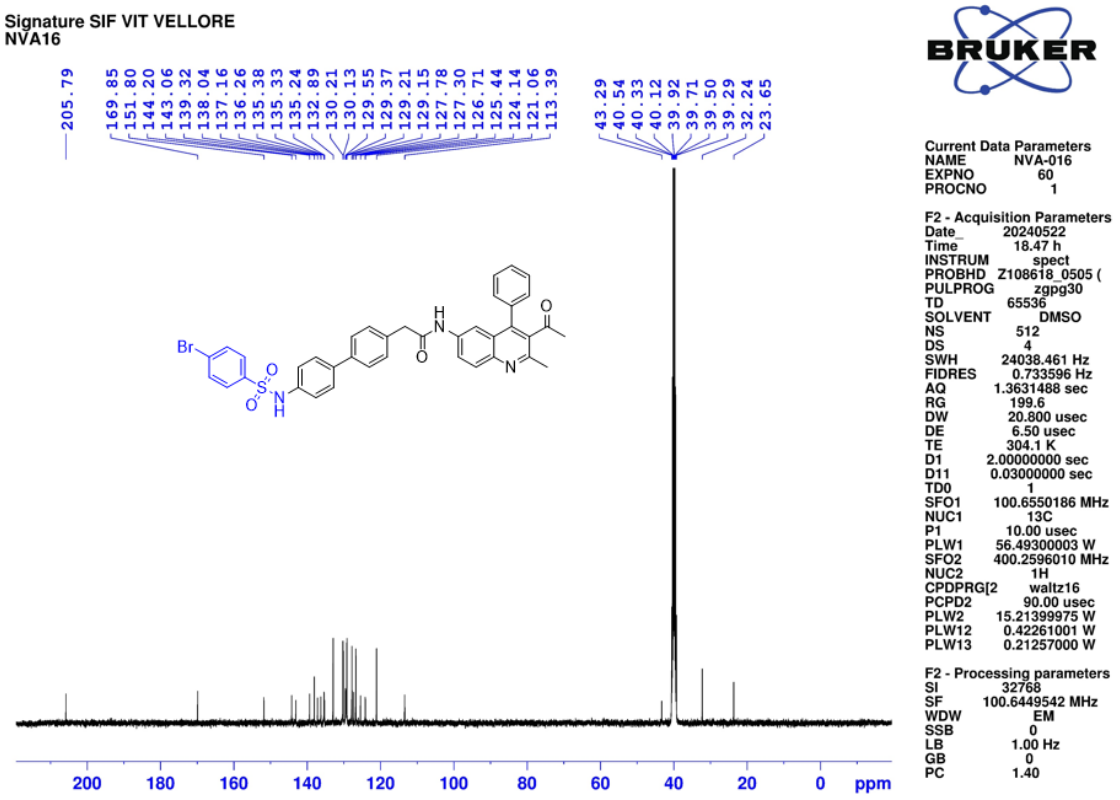

Fig. S27 ^{13}C NMR spectrum of compound 10f

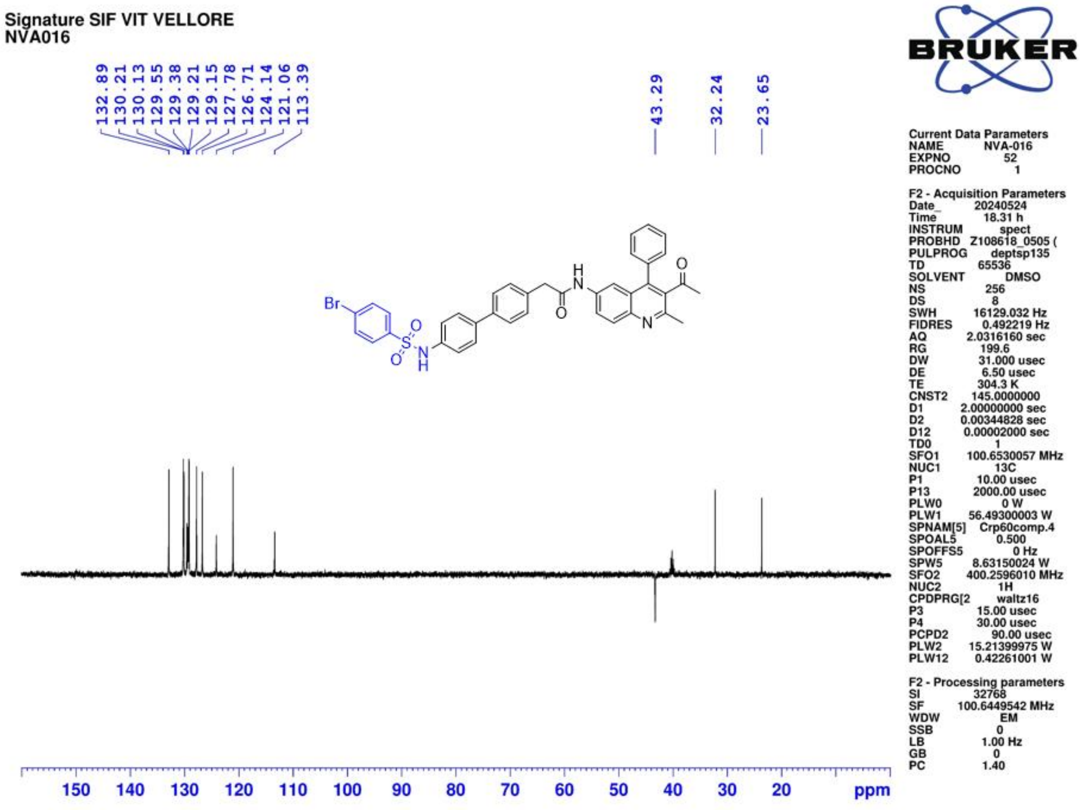

Fig. S28 DEPT-135 NMR spectrum of compound 10f

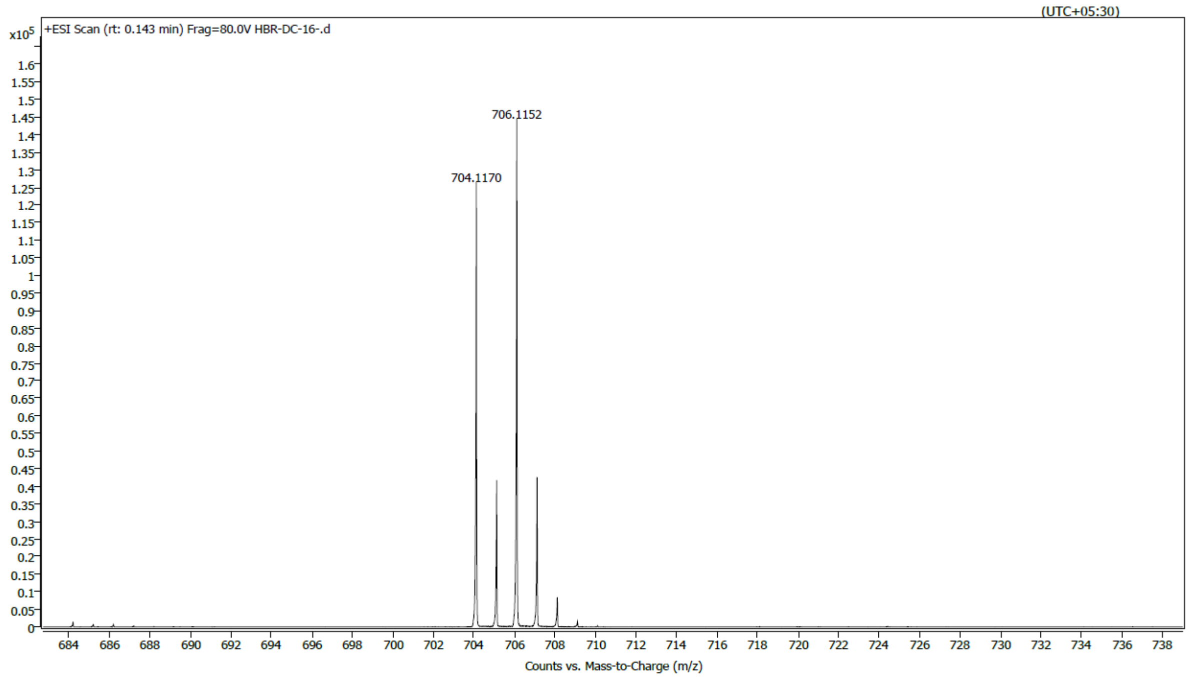

Fig. S29 HRMS of compound 10f

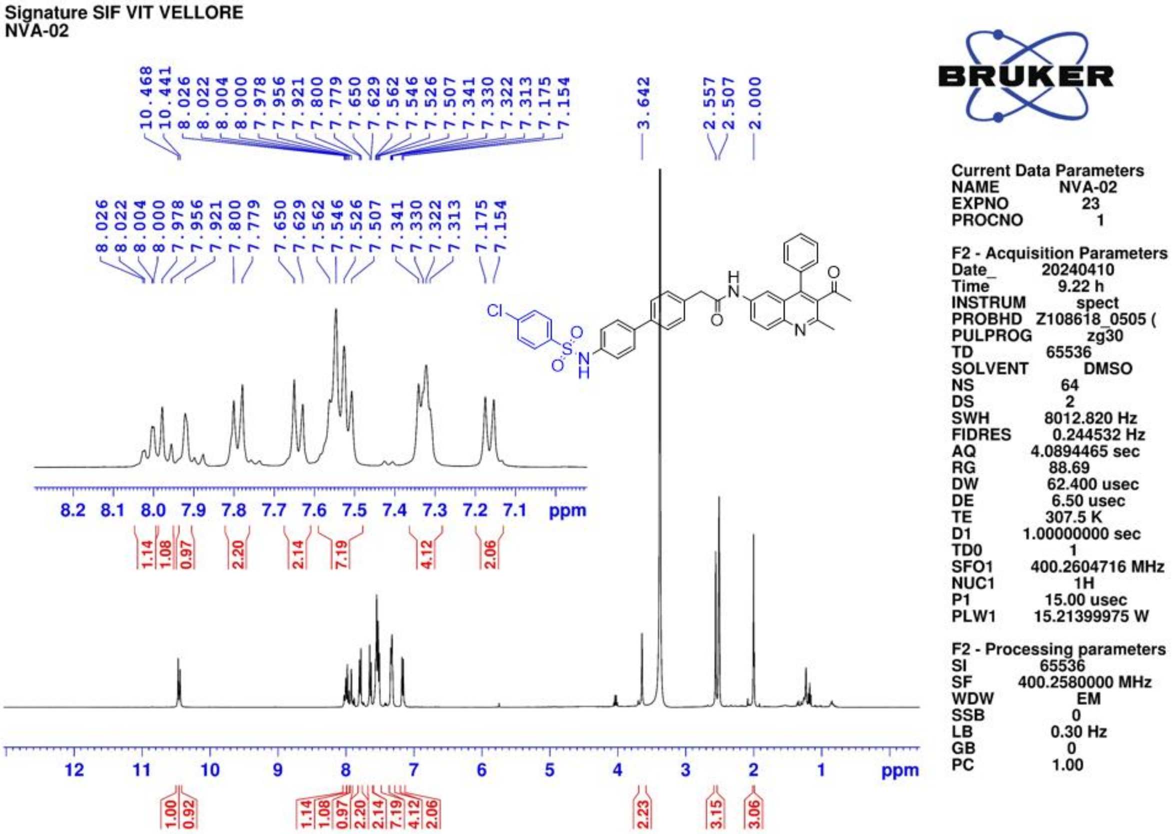

Fig. S30 ^{1}H NMR spectrum of compound 10g

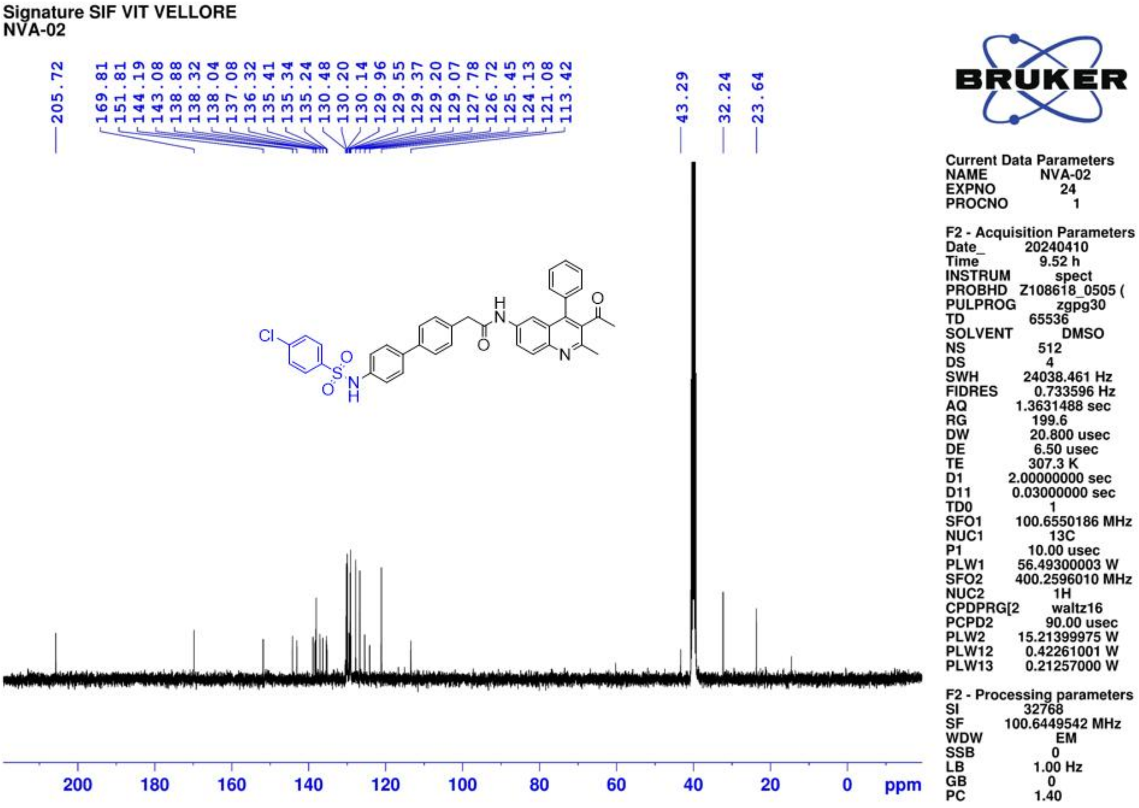

Fig. S31 ^{13}C NMR spectrum of compound 10g

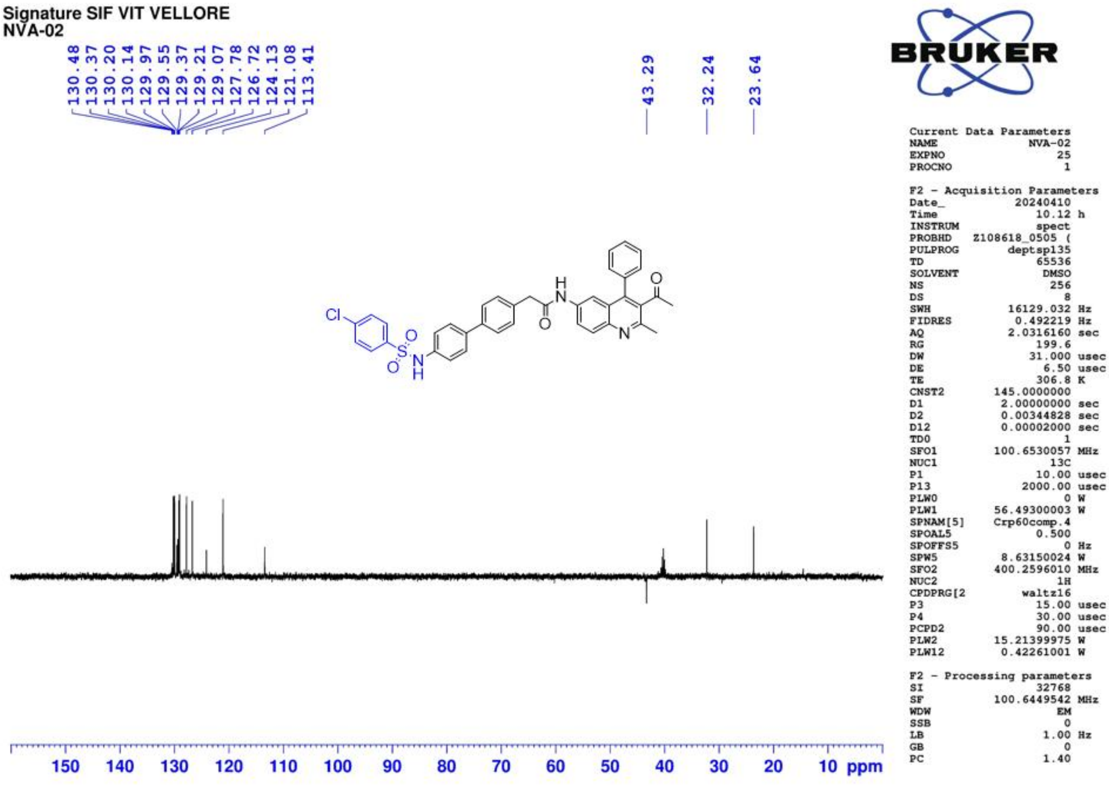

Fig. S32 DEPT-135 NMR spectrum of compound 10g

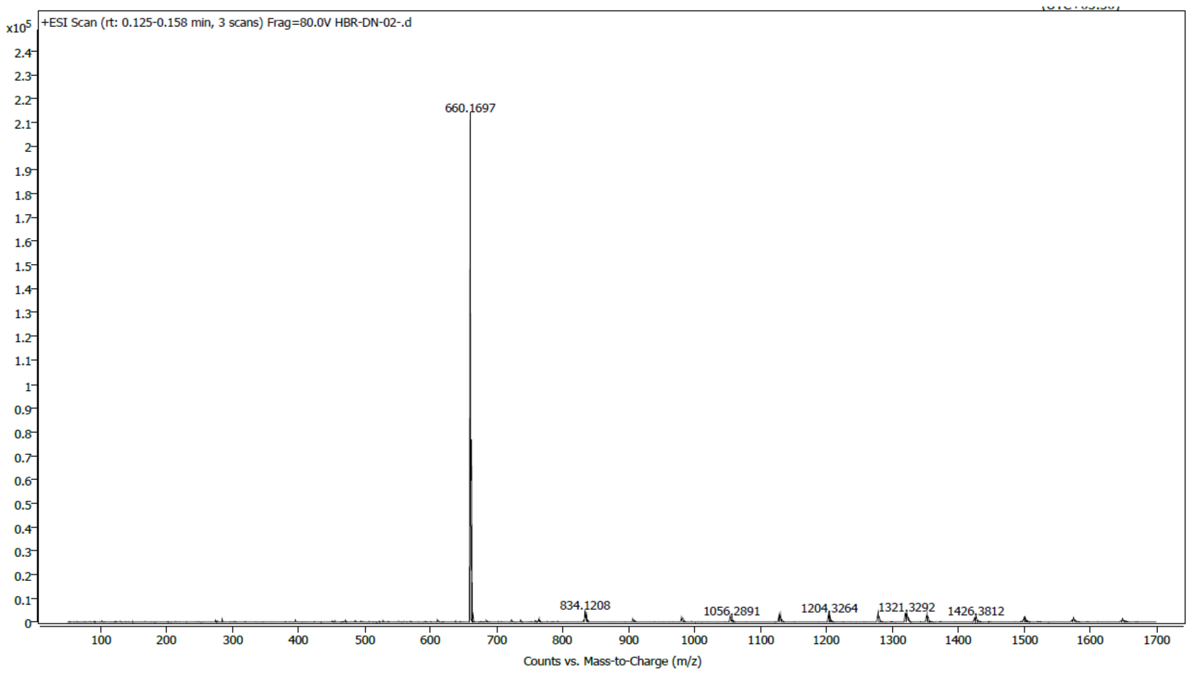

Fig. S33 HRMS of compound 10g

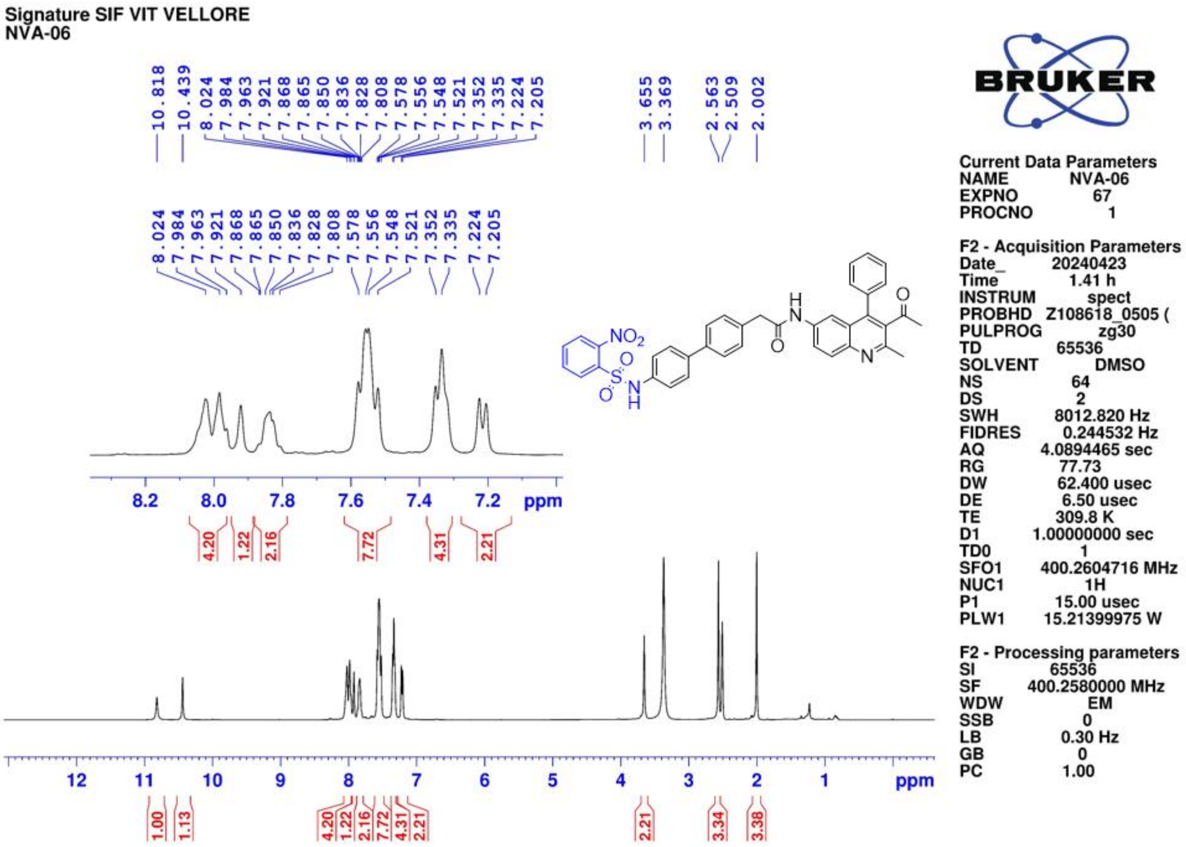

Fig. S34 ^{1}H NMR spectrum of compound 10h

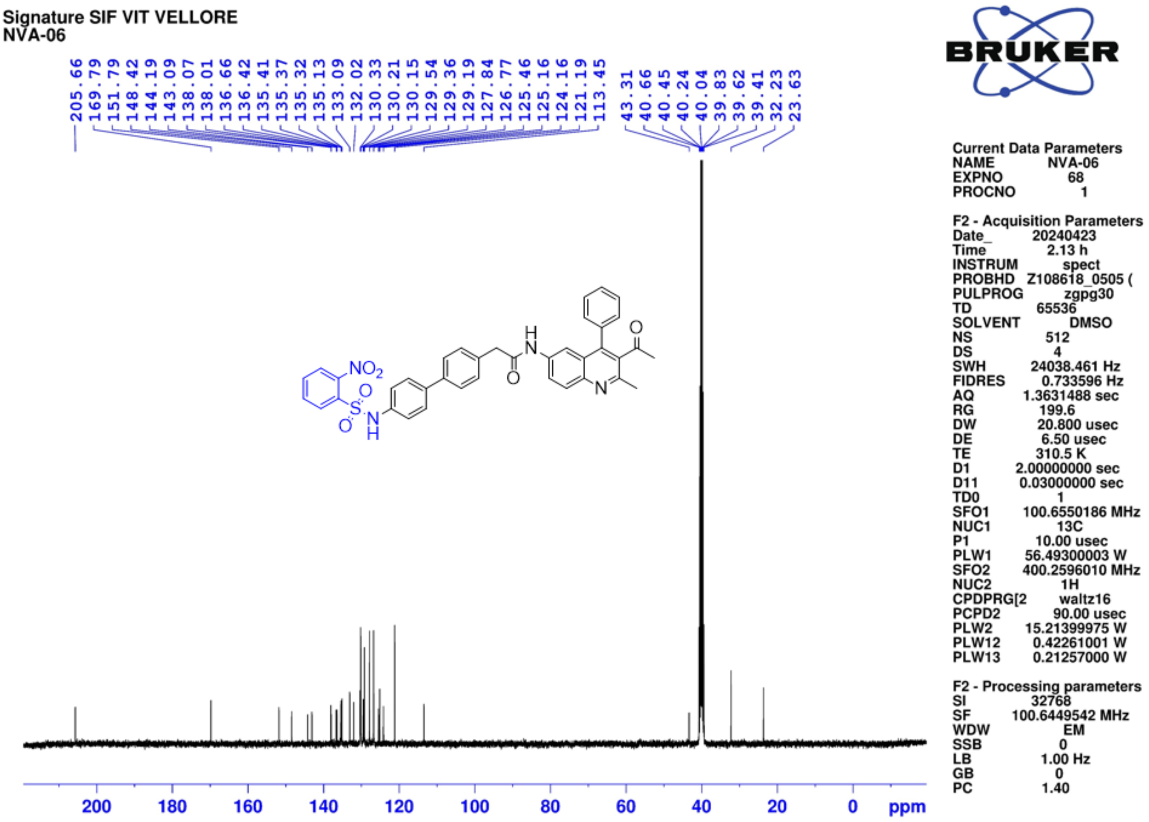

Fig. S35 ^{13}C NMR spectrum of compound 10h

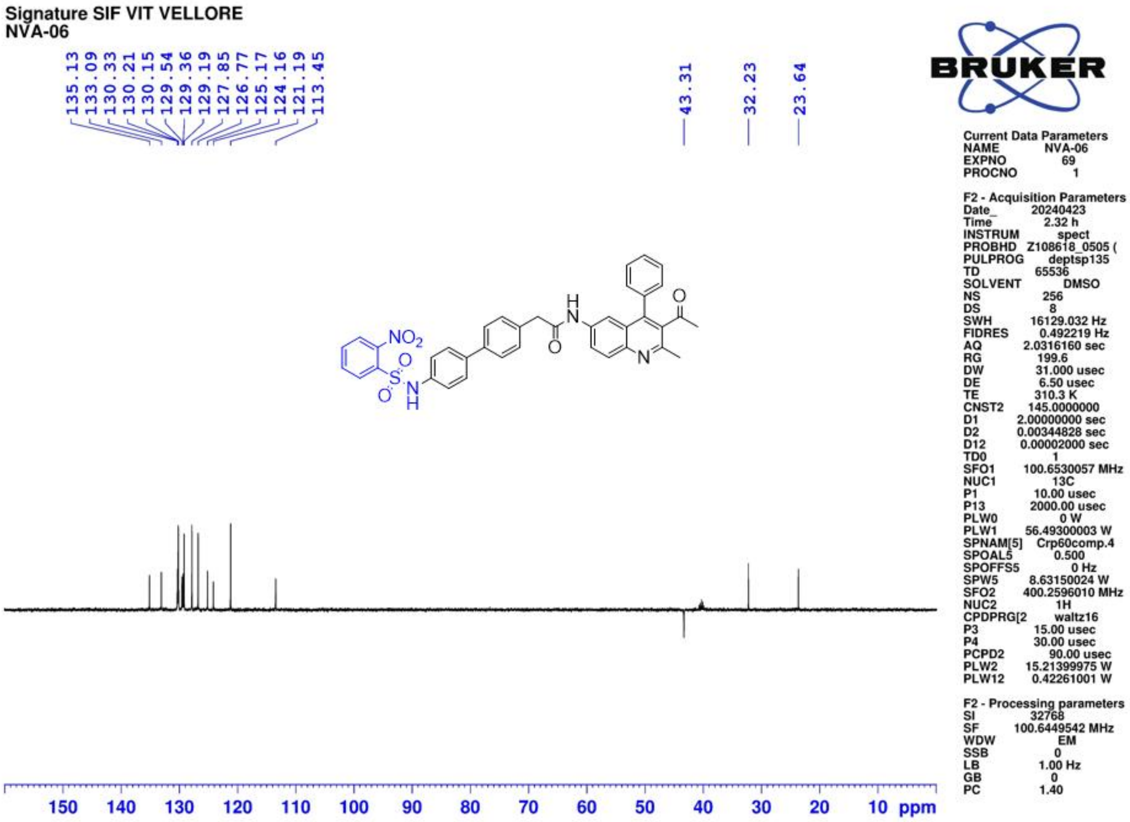

Fig. S36 DEPT-135 NMR spectrum of compound 10h

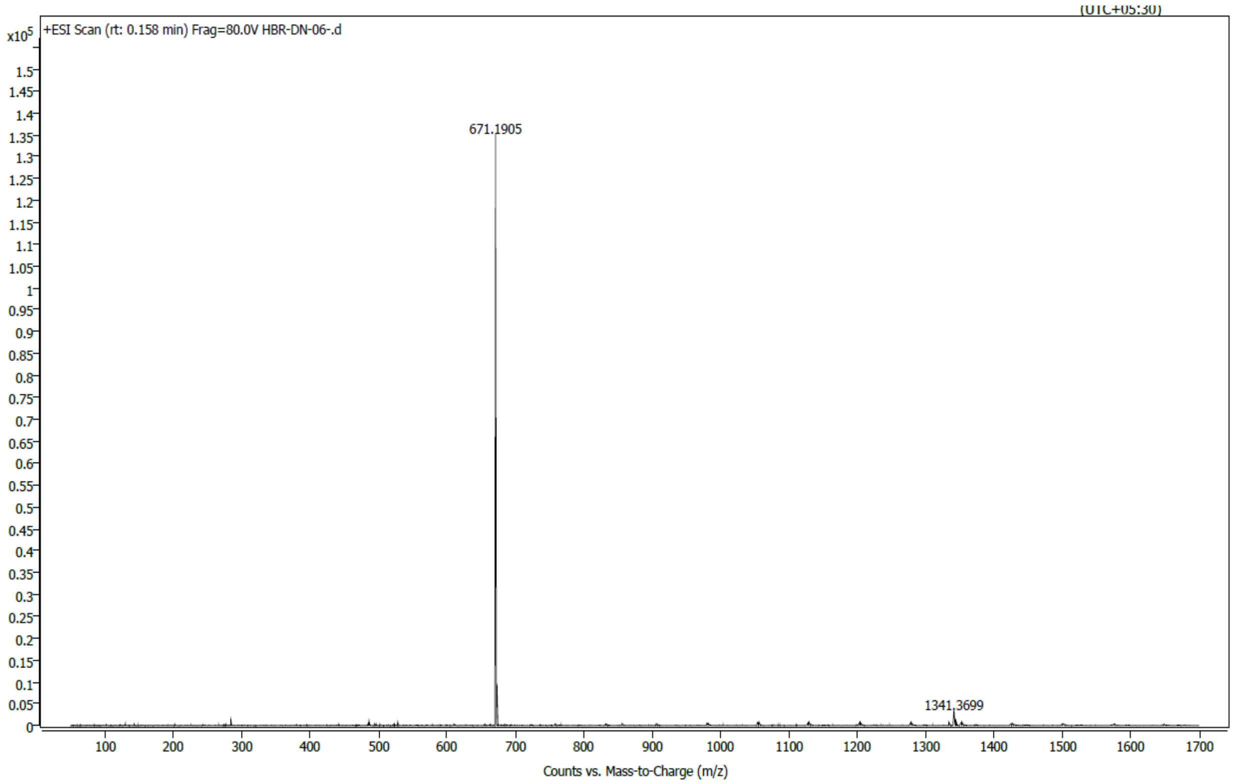

Fig. S37 HRMS of compound 10h

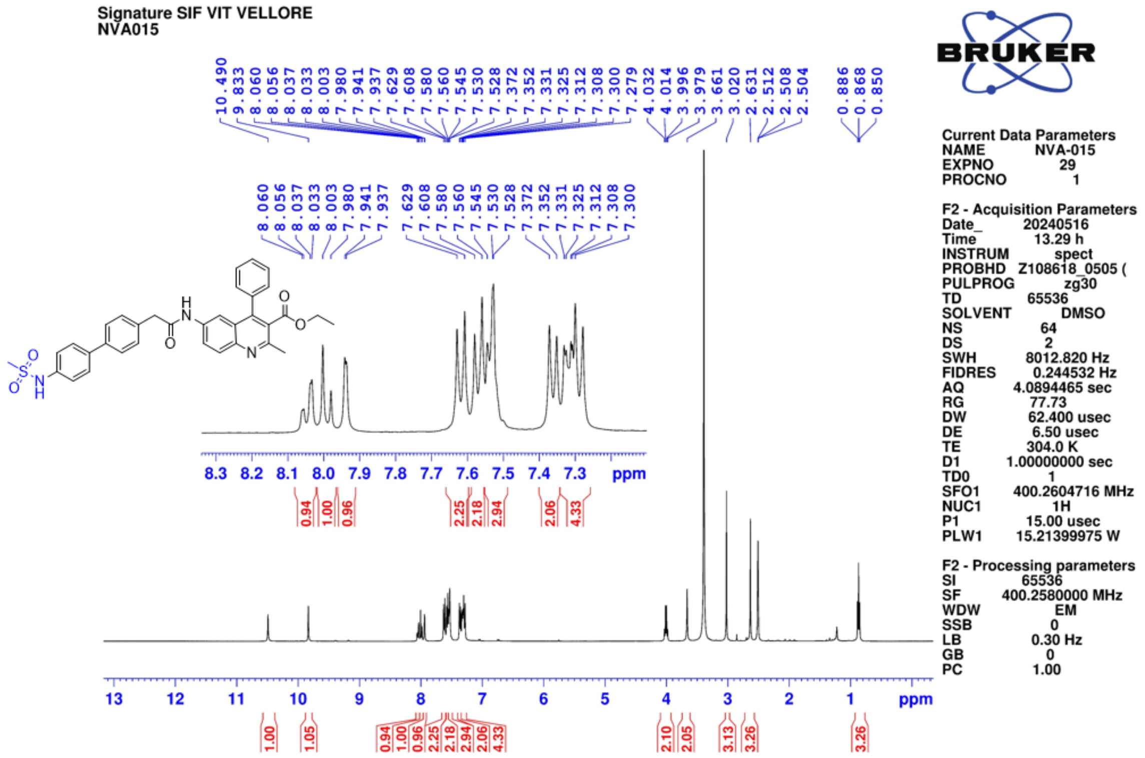

Fig. S38 ^{1}H NMR spectrum of compound 10i

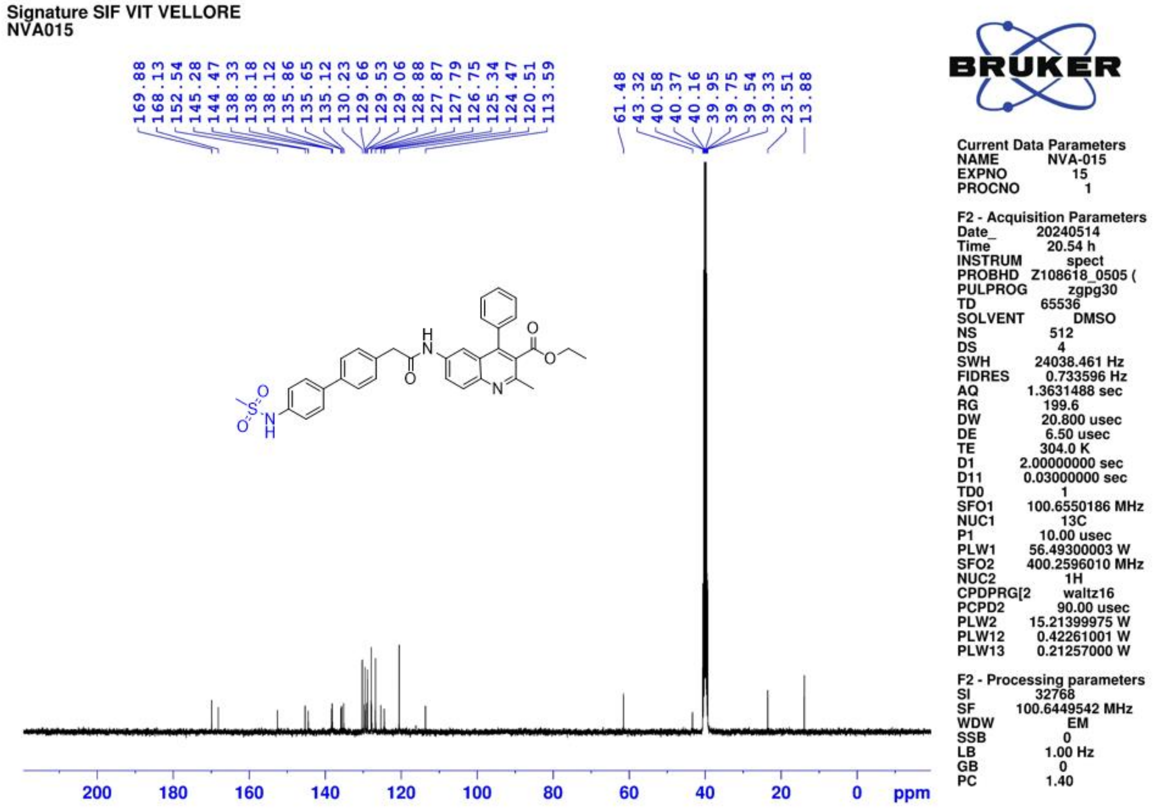

Fig. S39 ^{13}C NMR spectrum of compound 10i

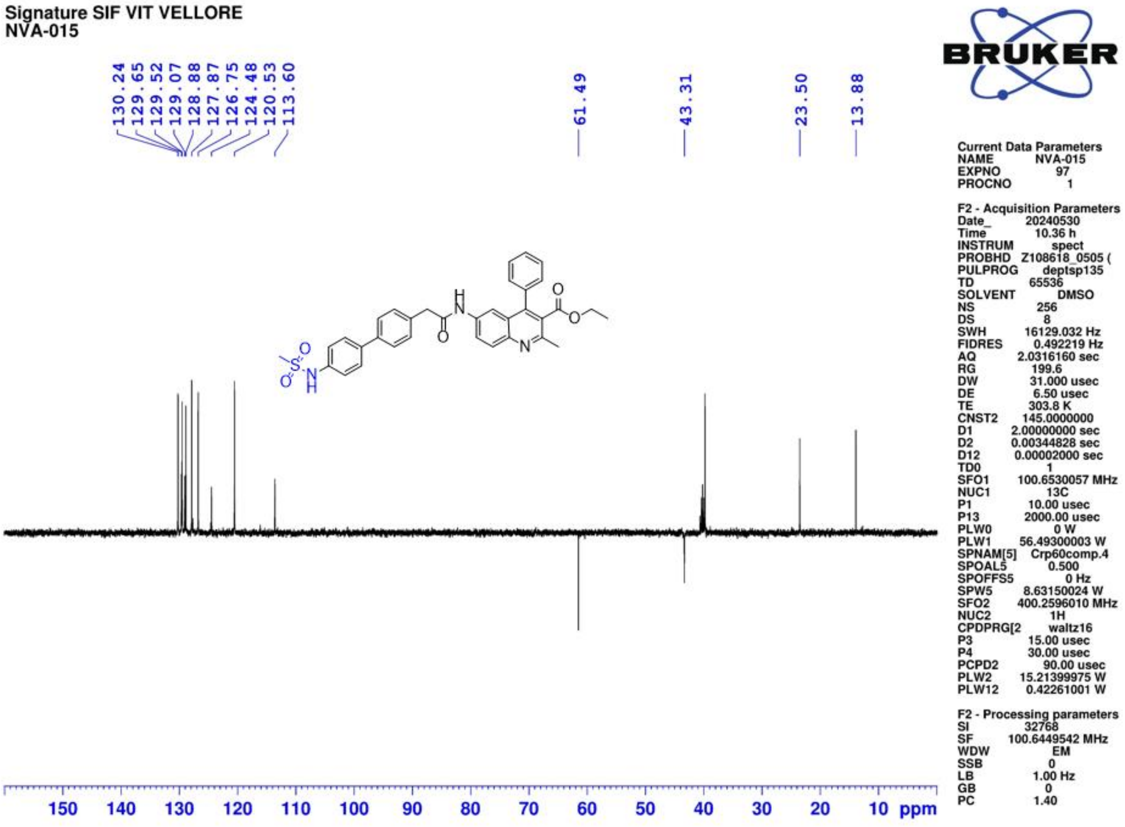

Fig. S40 DEPT-135 NMR spectrum of compound 10i

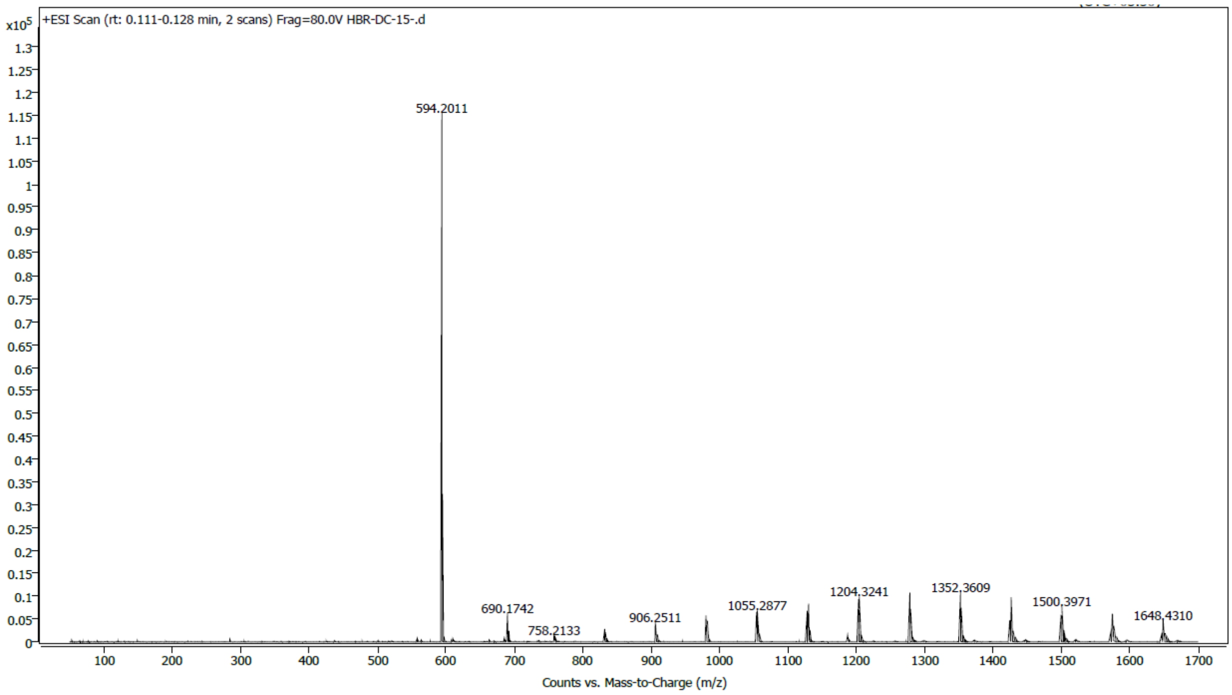

Fig. S41 HRMS of compound 10i

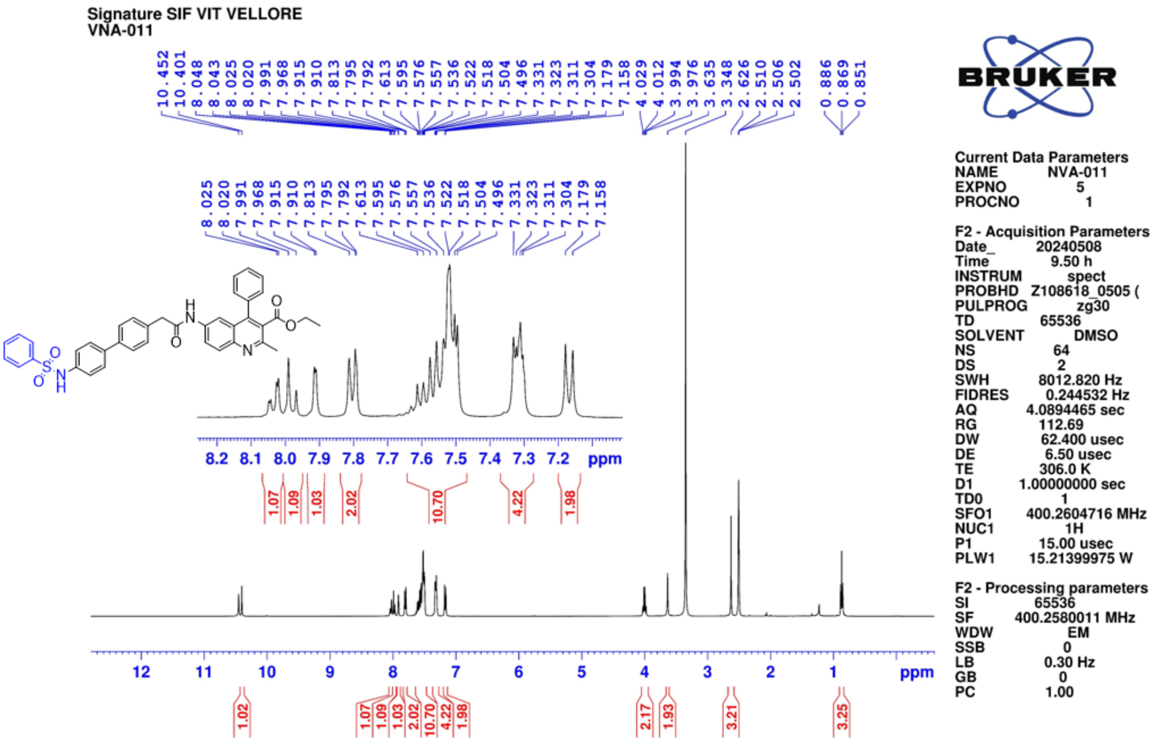

Fig. S42 ^{1}H NMR spectrum of compound 10j

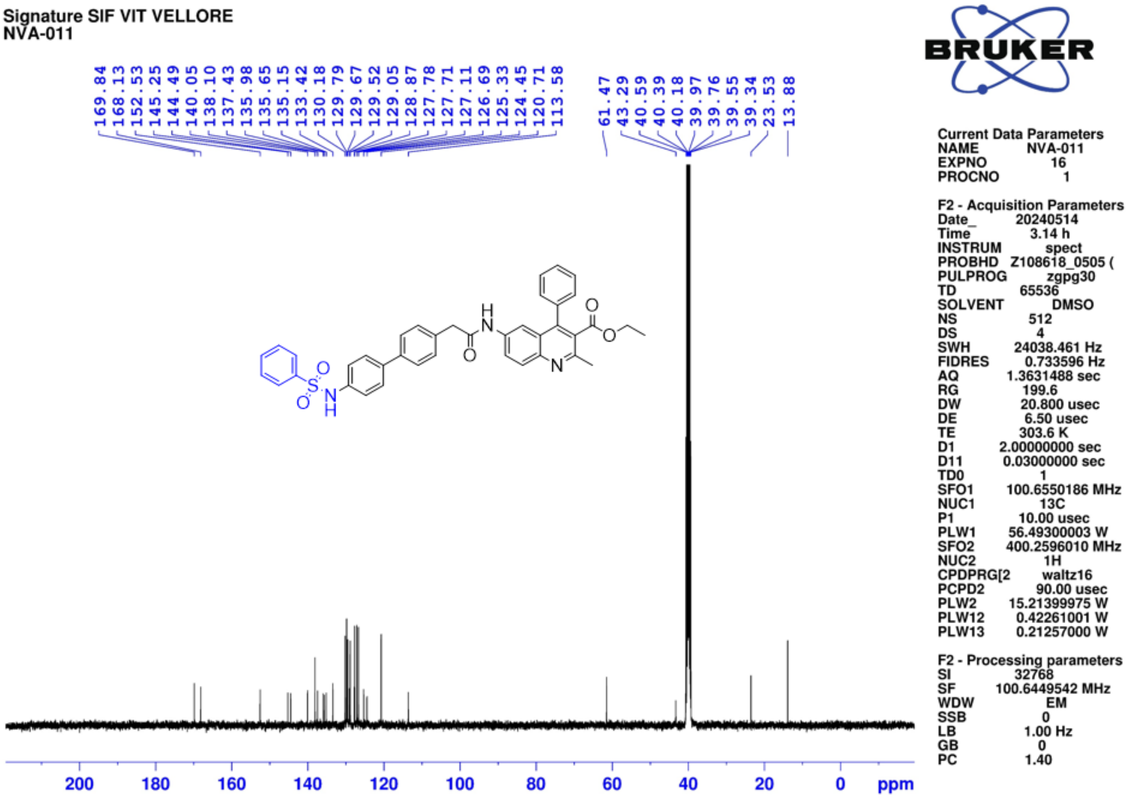

Fig. S43 ^{13}C NMR spectrum of compound 10j

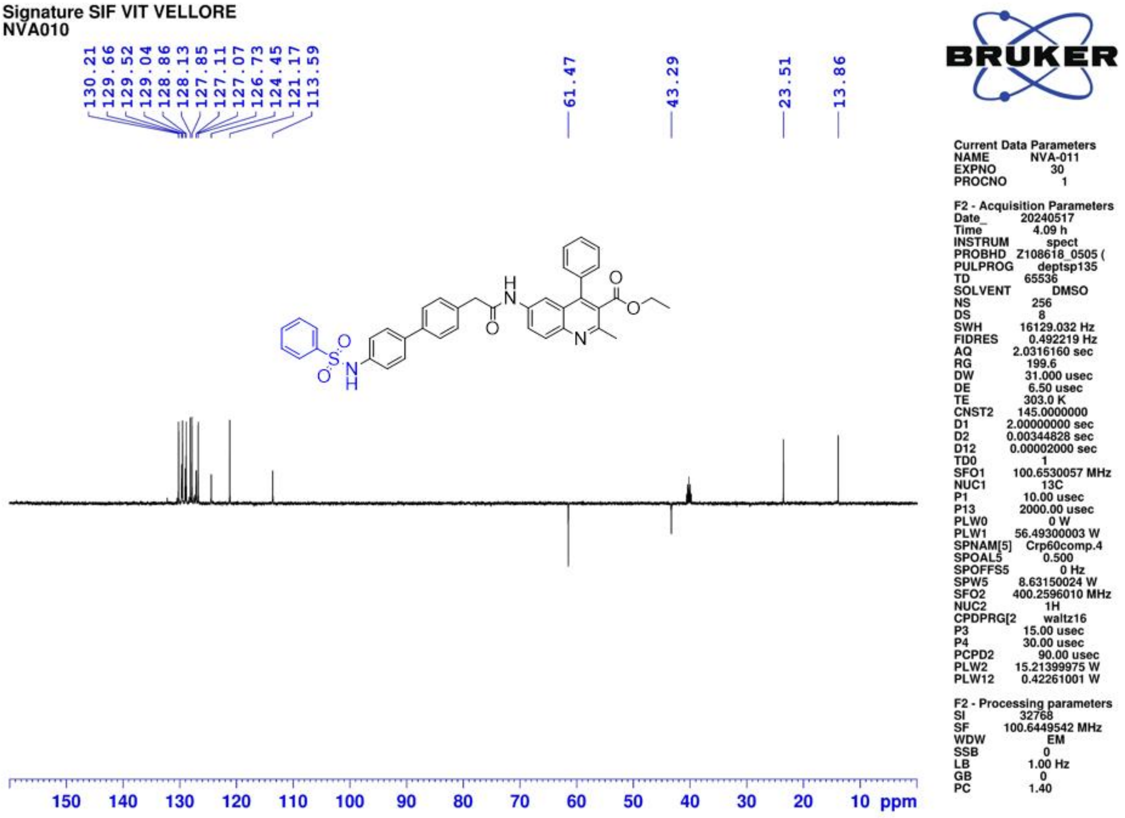

Fig. S44 DEPT-135 spectrum of compound 10j

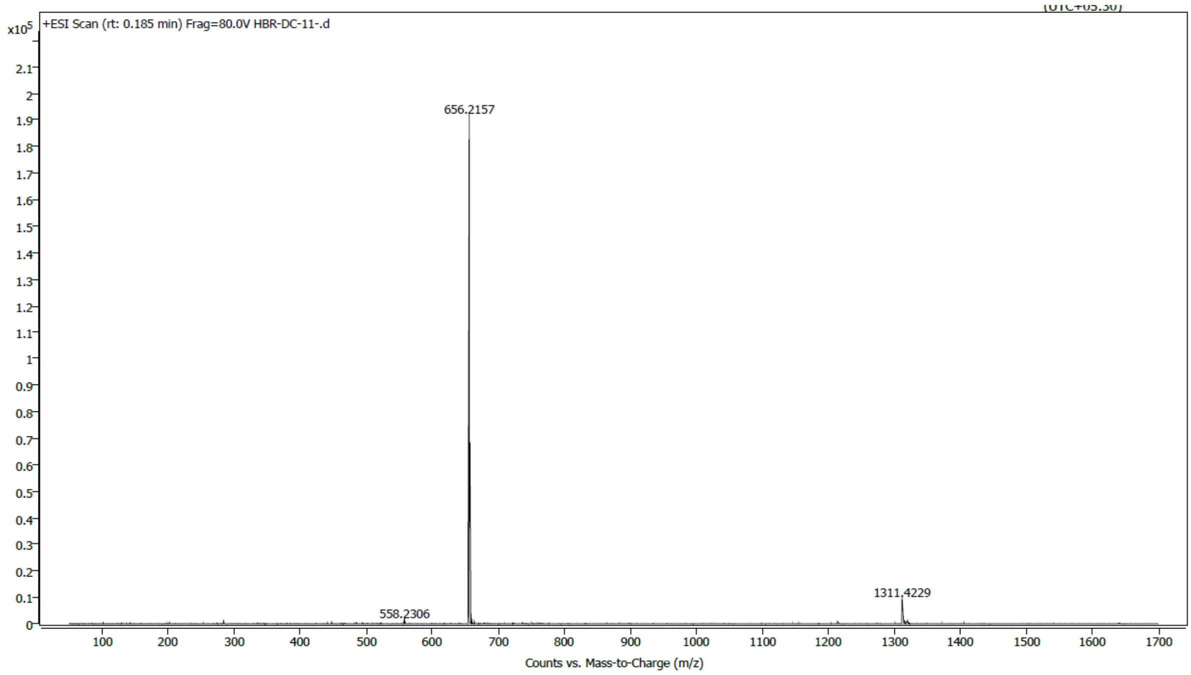

Fig. S45 HRMS of compound 10j

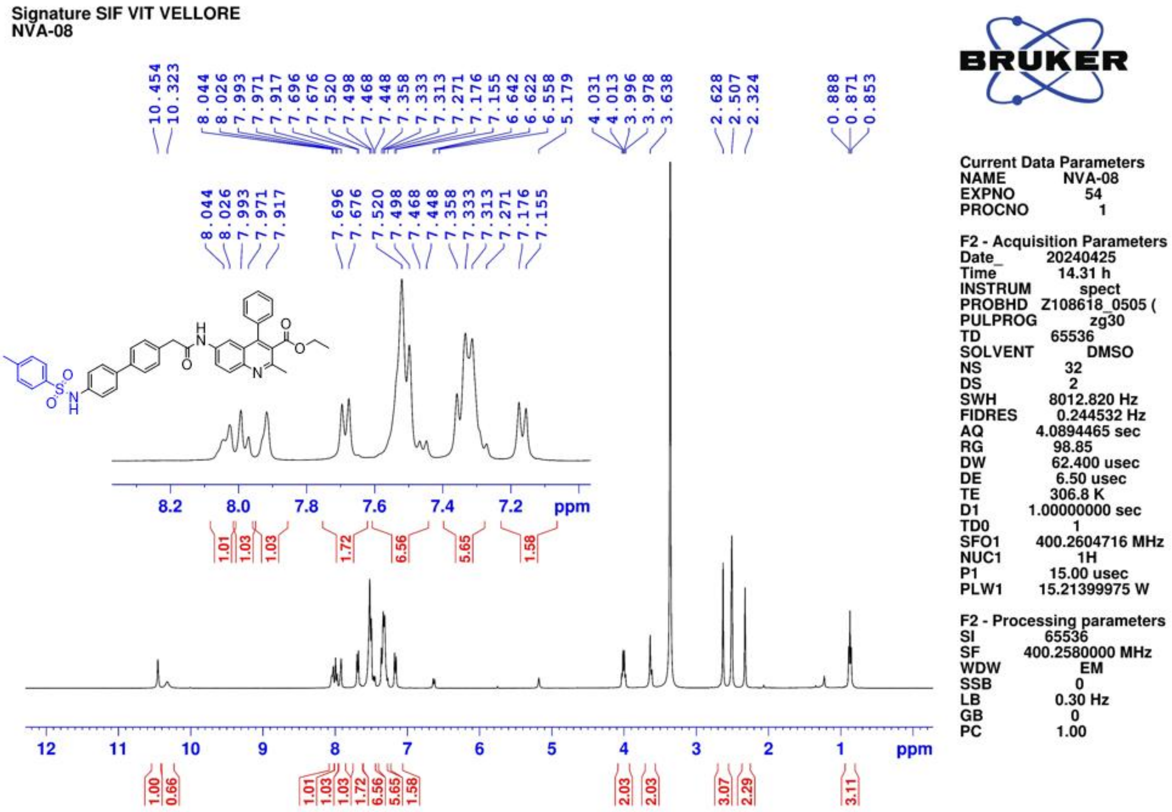

Fig. S46 ^{1}H NMR spectrum of compound 10k

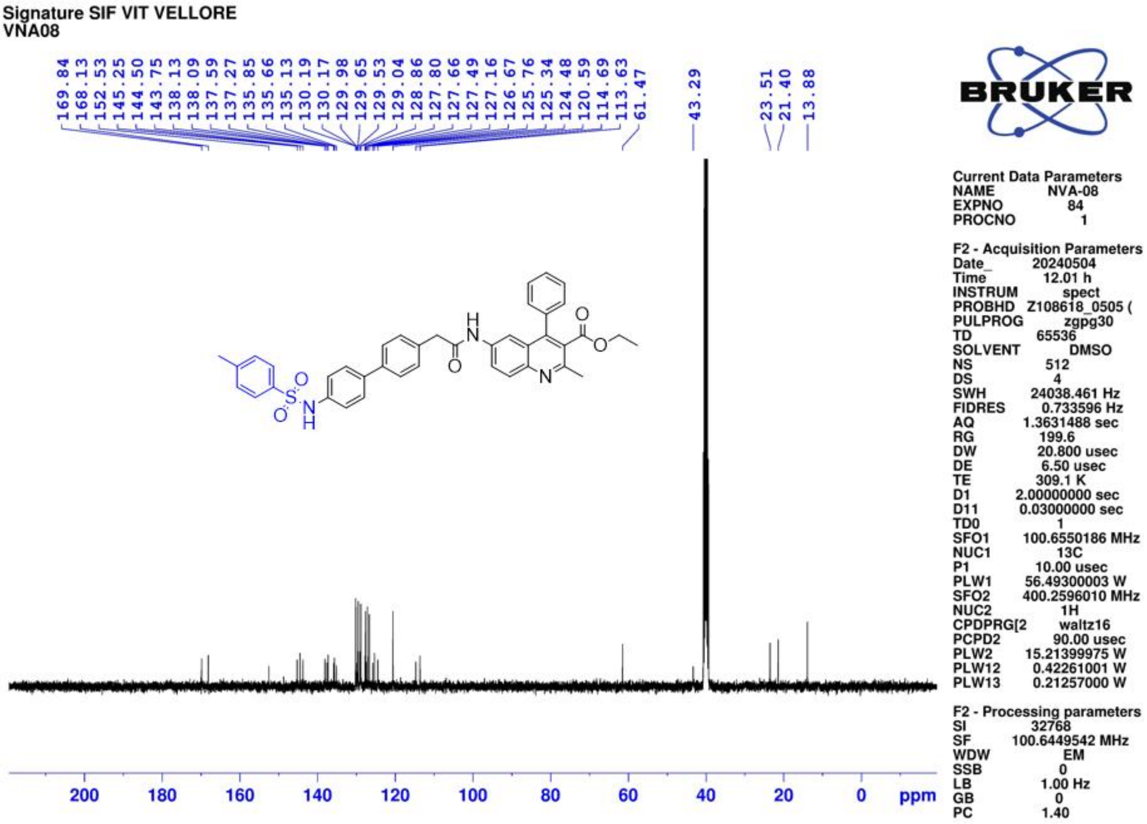

Fig. S47 ^{13}C NMR spectrum of compound 10k

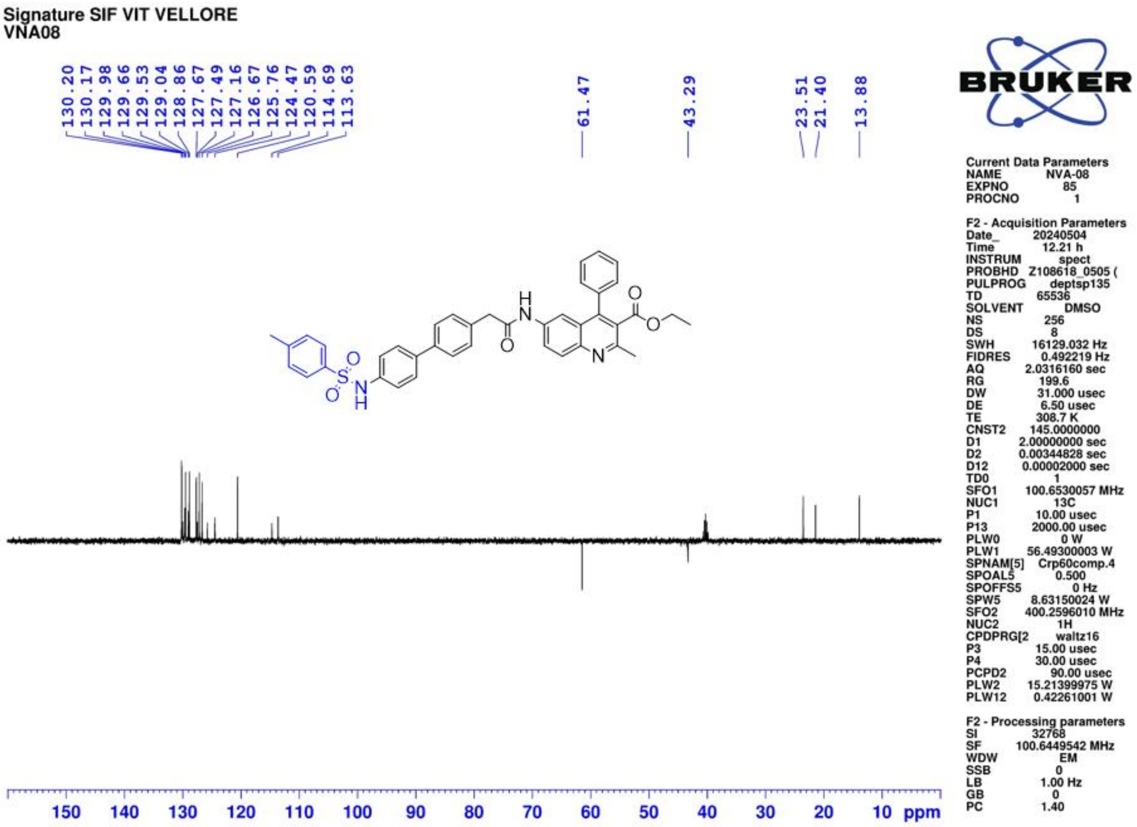

Fig. S48 DEPT-135 NMR spectrum of compound 10k

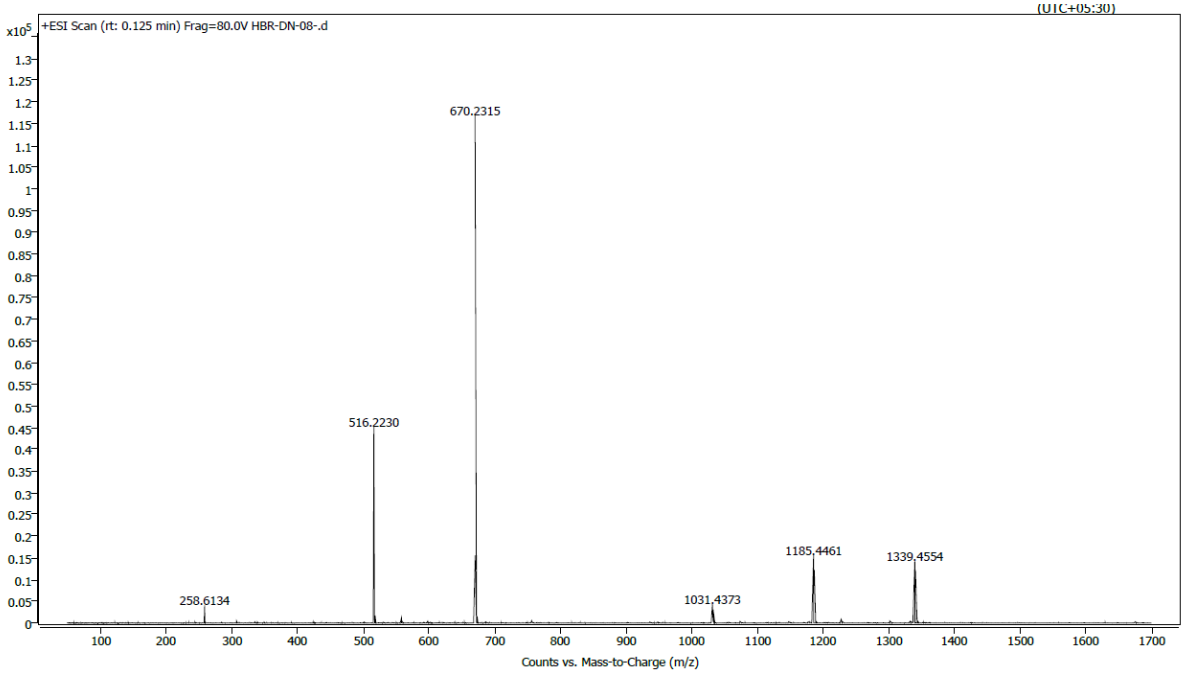

Fig. S49 HRMS of compound 10k

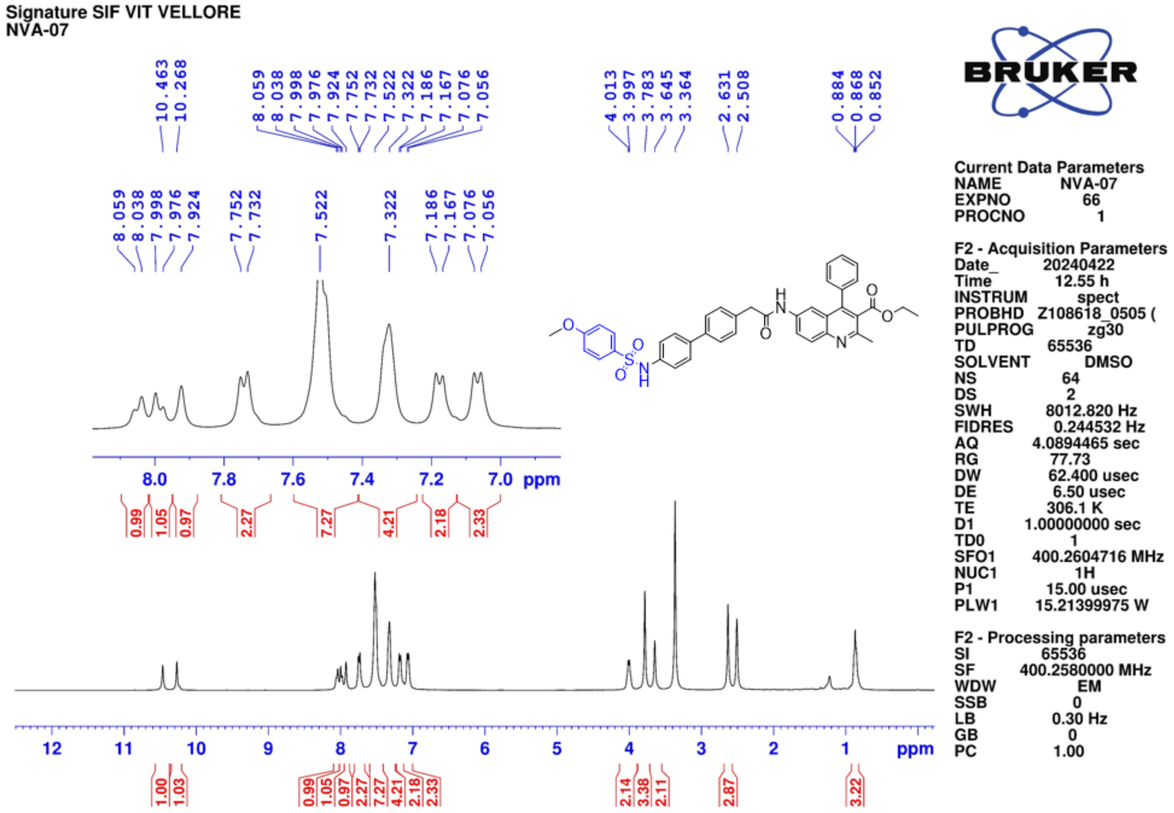

Fig. S50 ^{1}H NMR spectrum of compound 10l

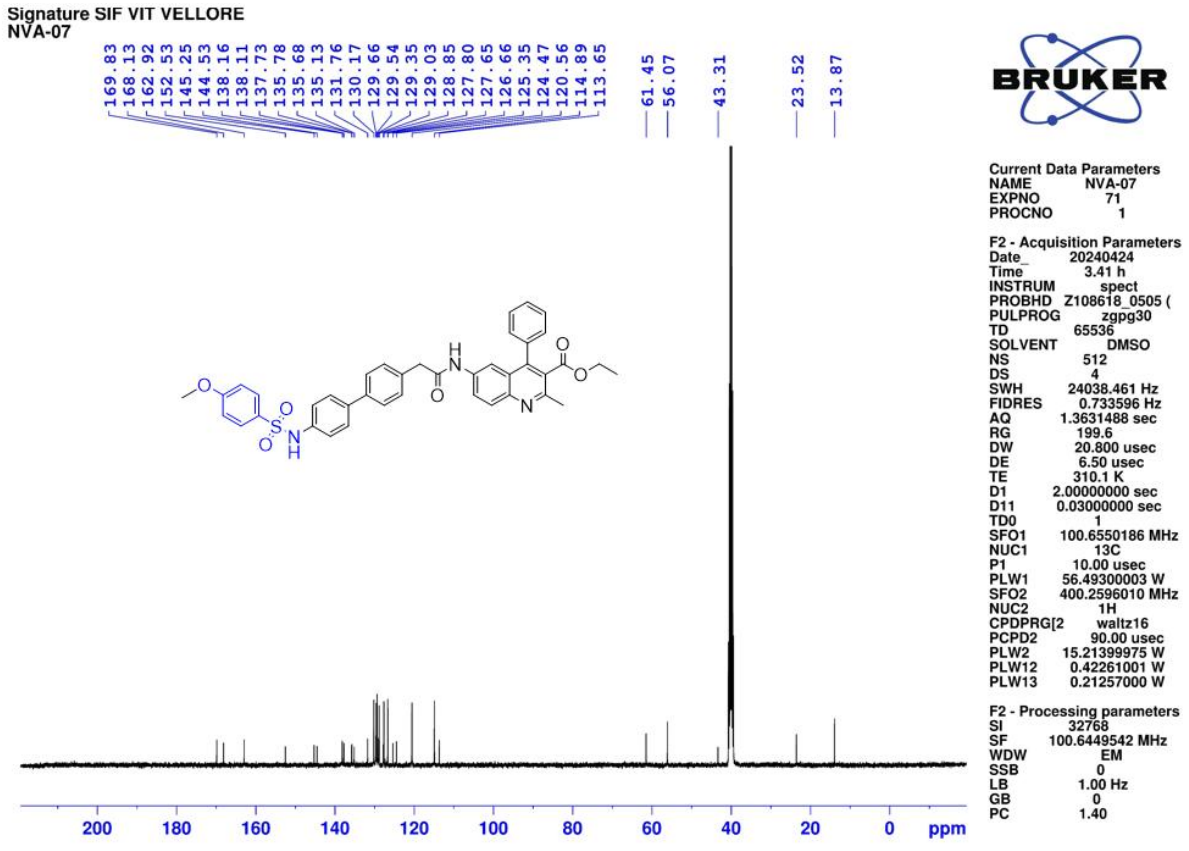

Fig. S51 ^{13}C NMR spectrum of compound 10l

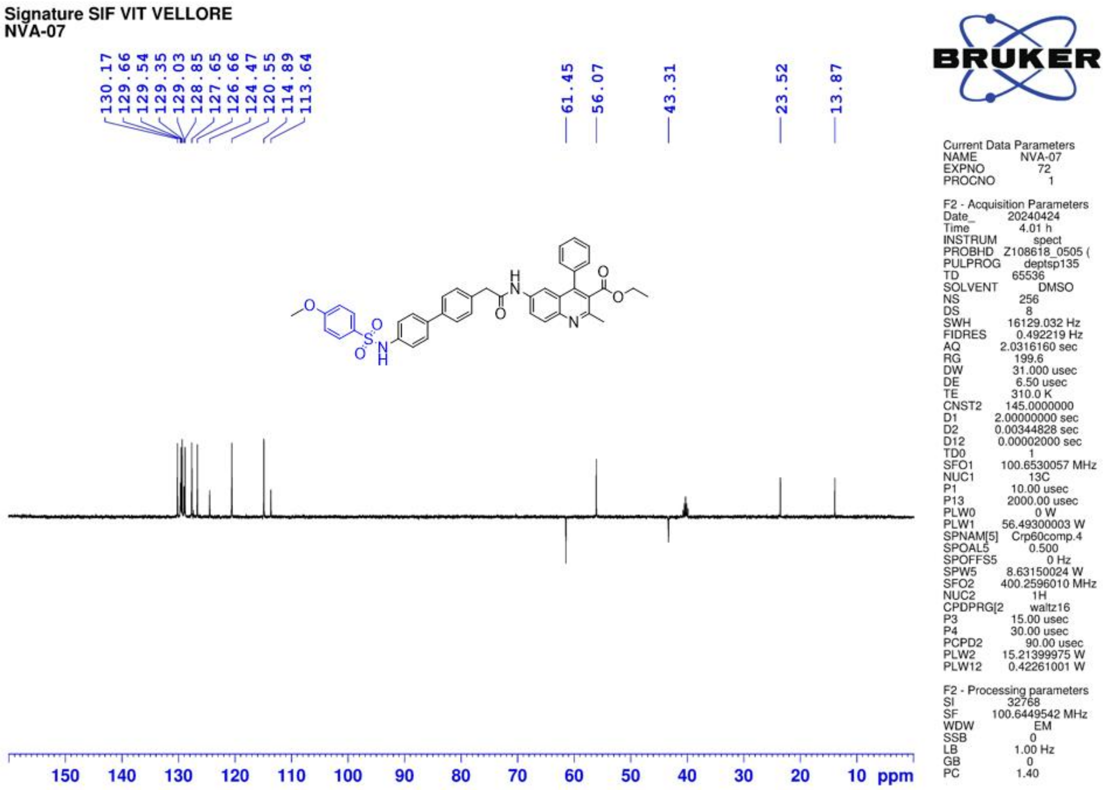

Fig. S52 DEPT-135 of compound 10l

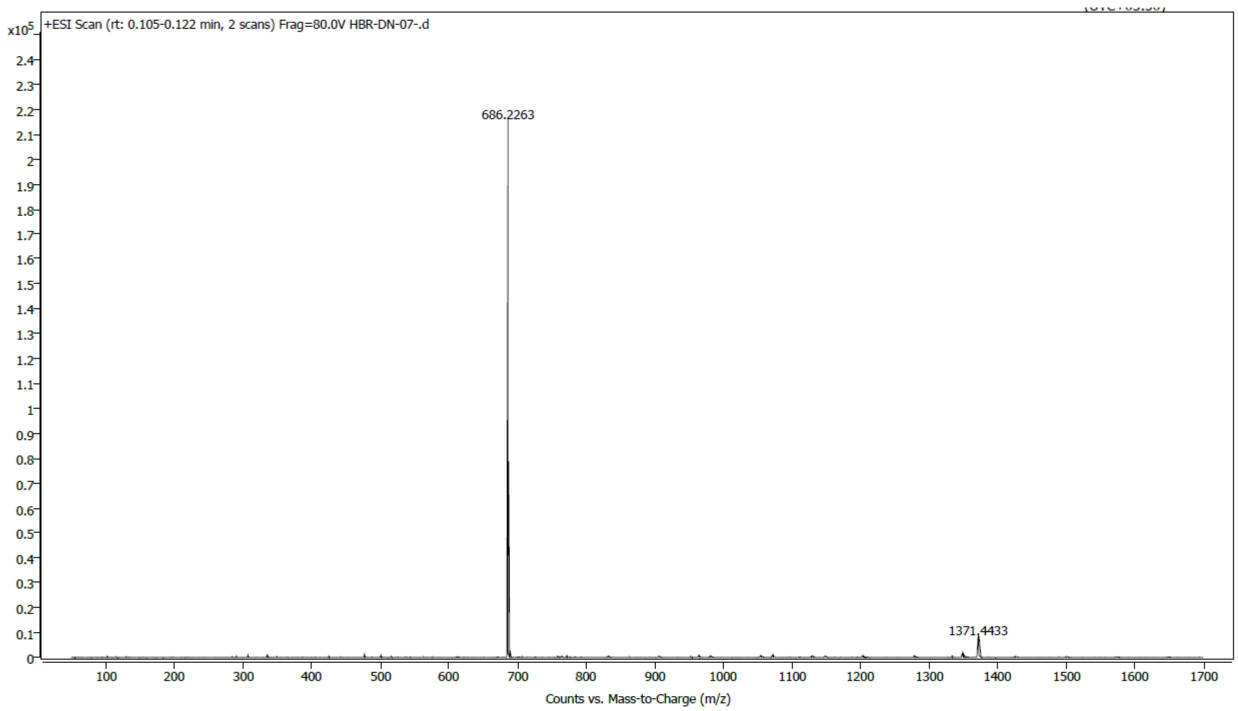

Fig. S53 HRMS of compound 10l

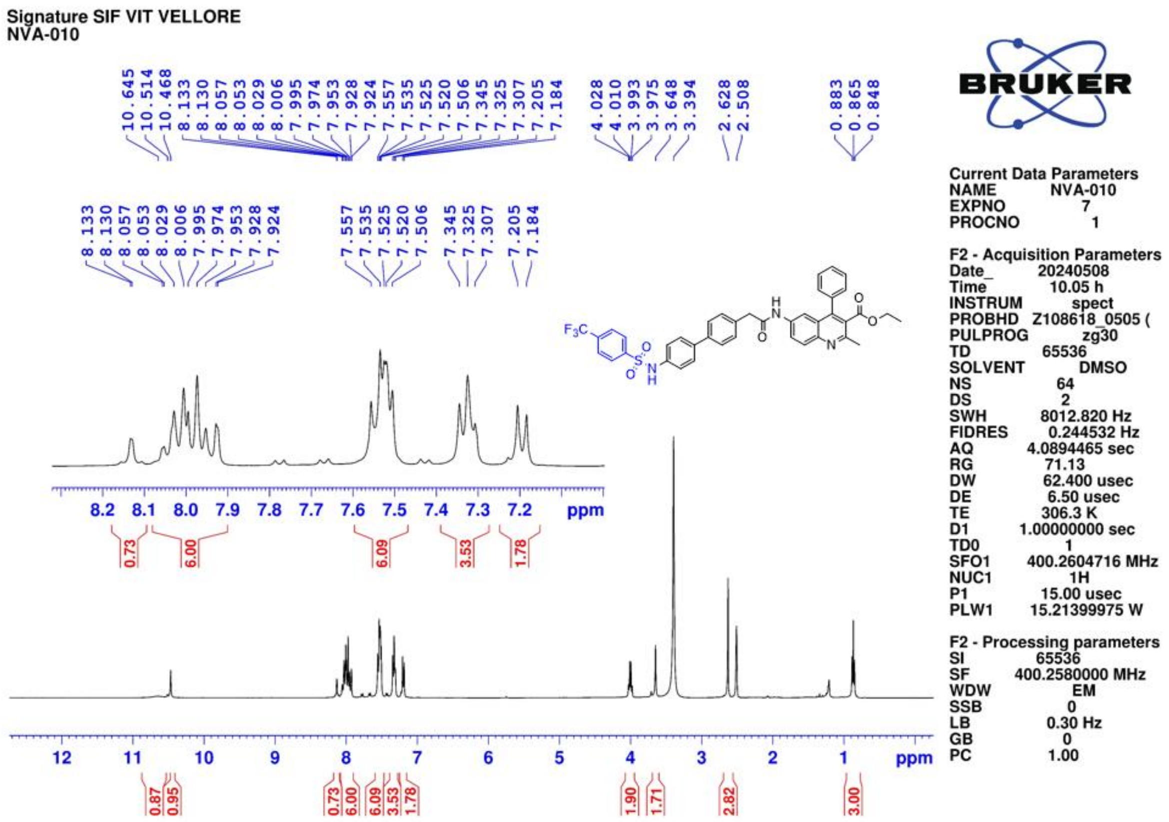

Fig. S54 ^{1}H NMR of compound 10m

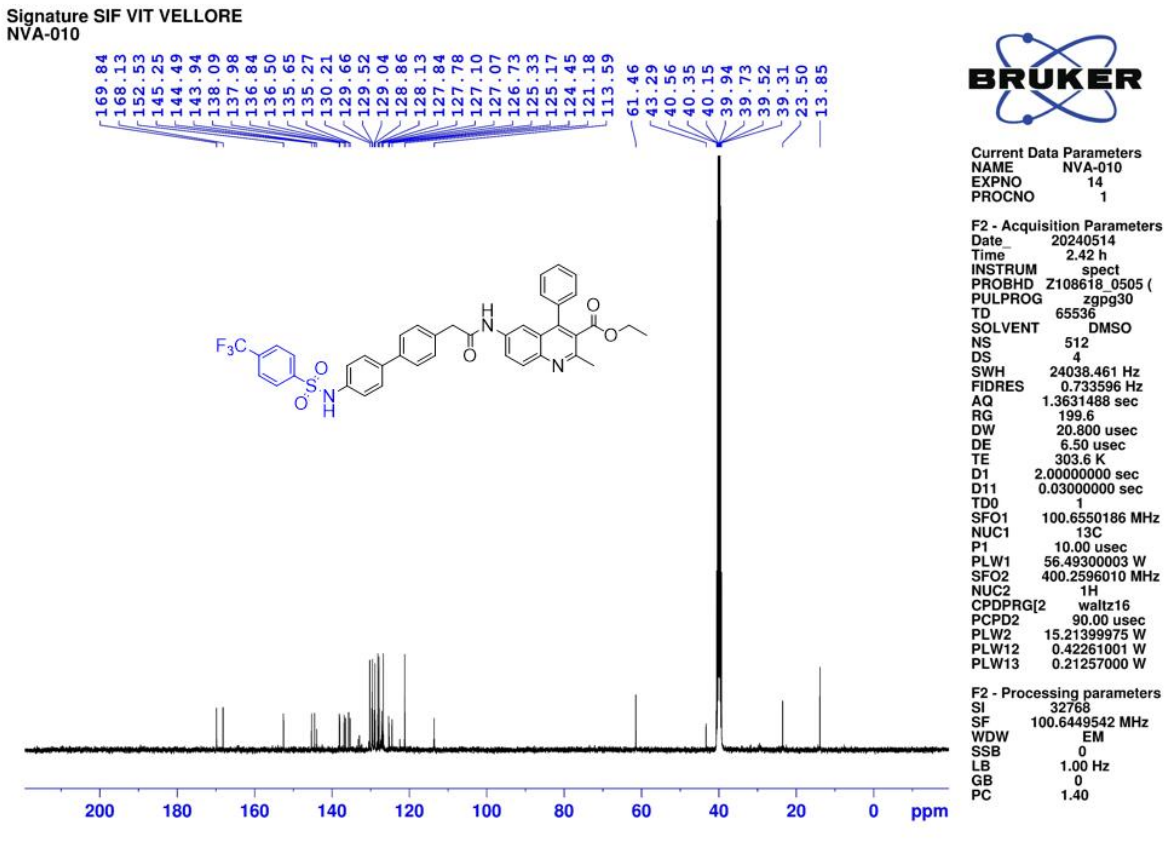

Fig. S55 ^{13}C NMR of compound 10m

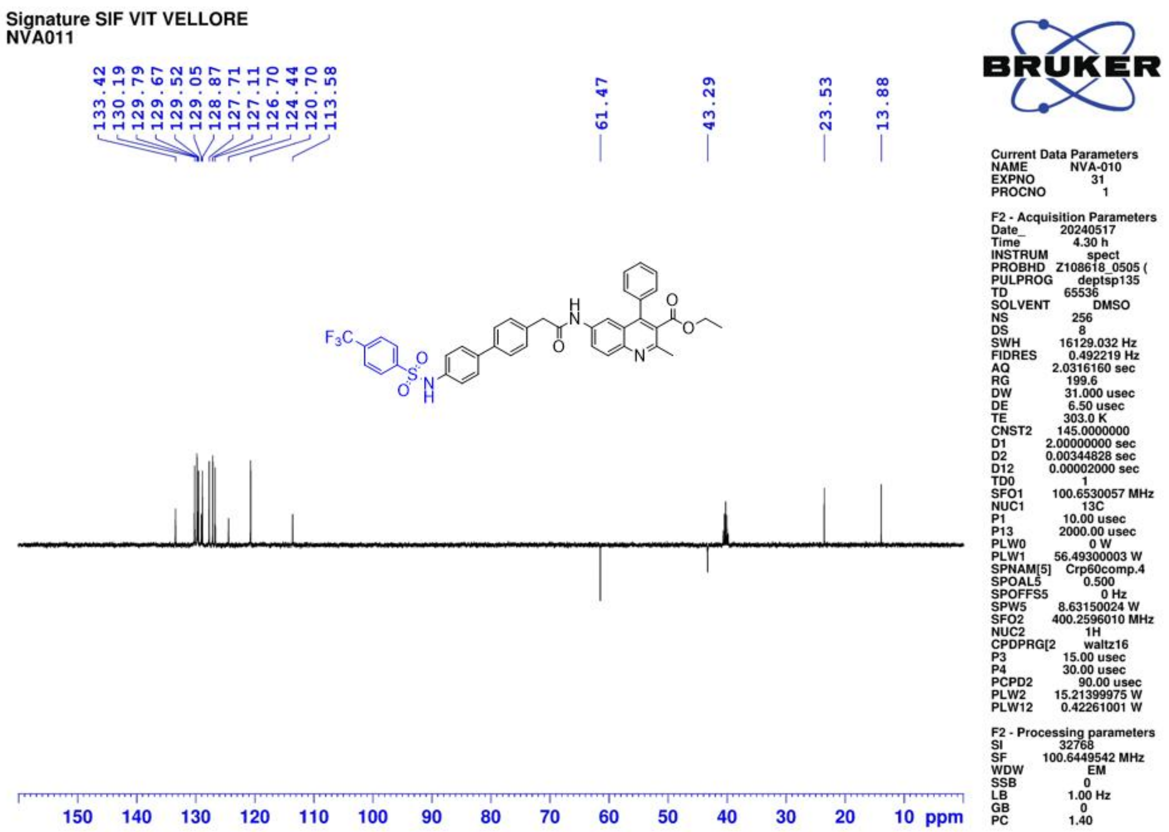

Fig. S56 DEPT-135 NMR spectrum of compound 10m

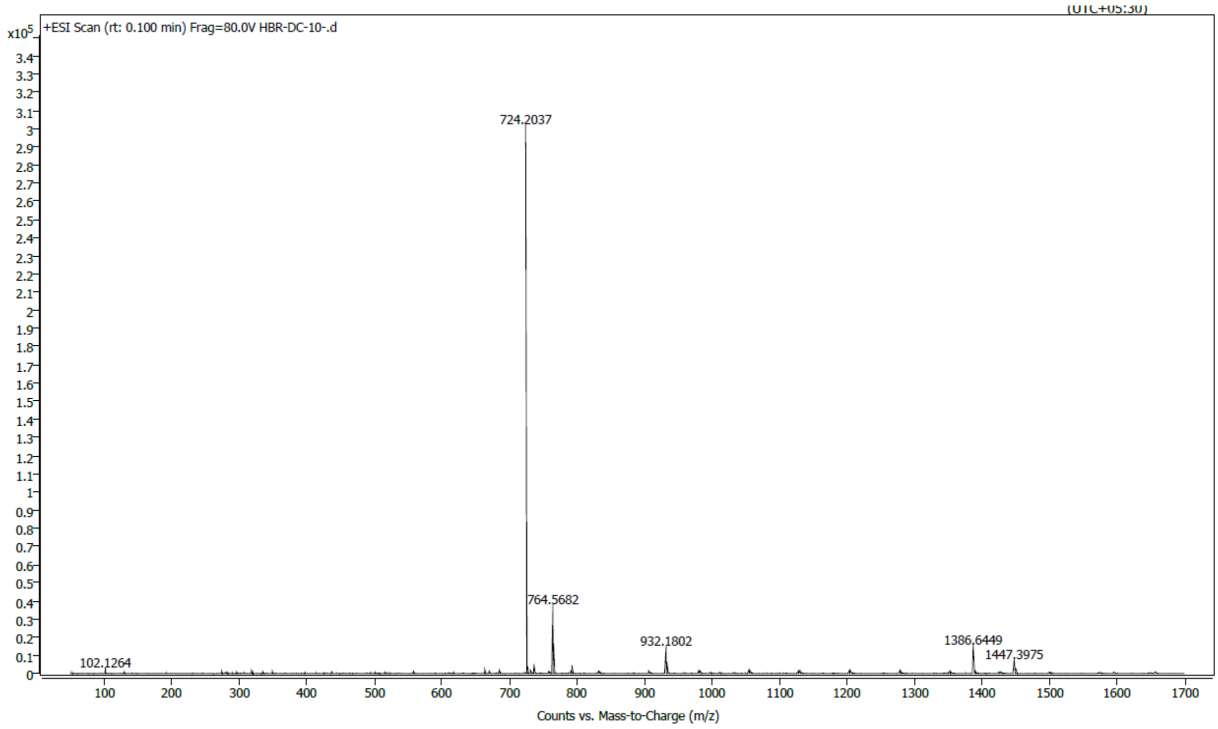

Fig. S57 HRMS of compound 10m

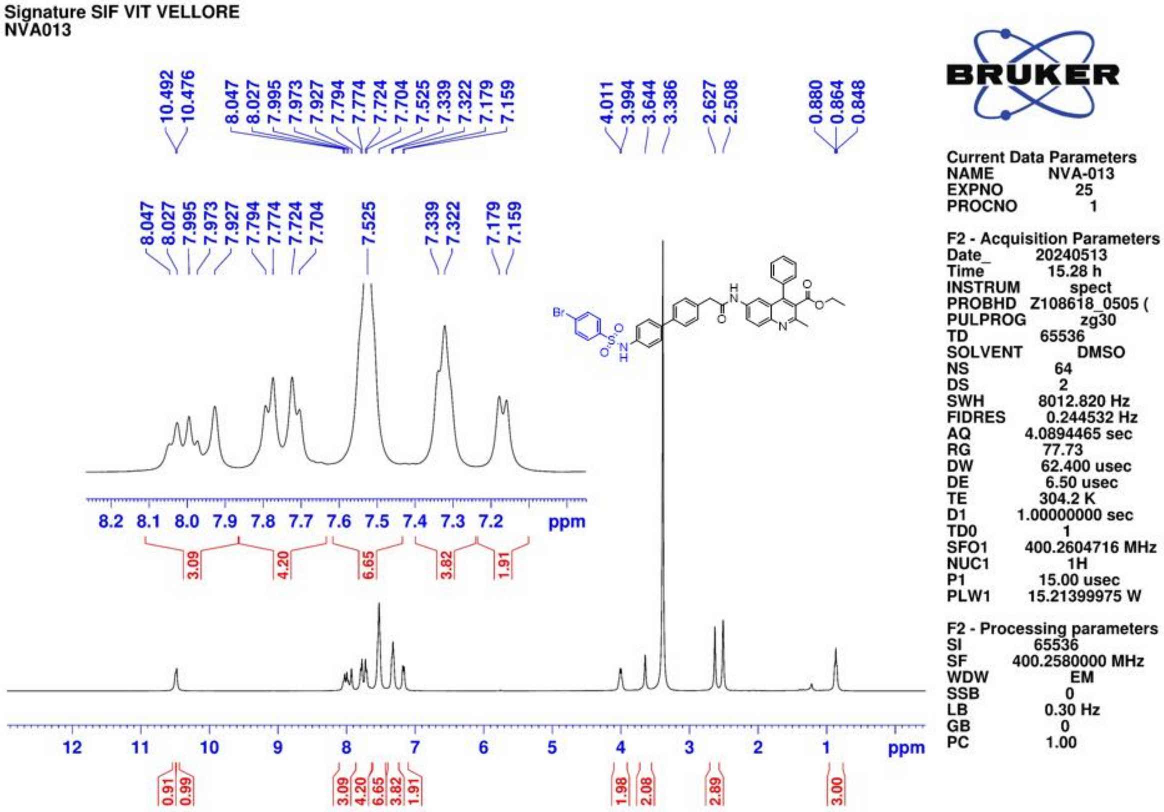

Fig. S58 ^{1}H NMR spectrum of compound 10n

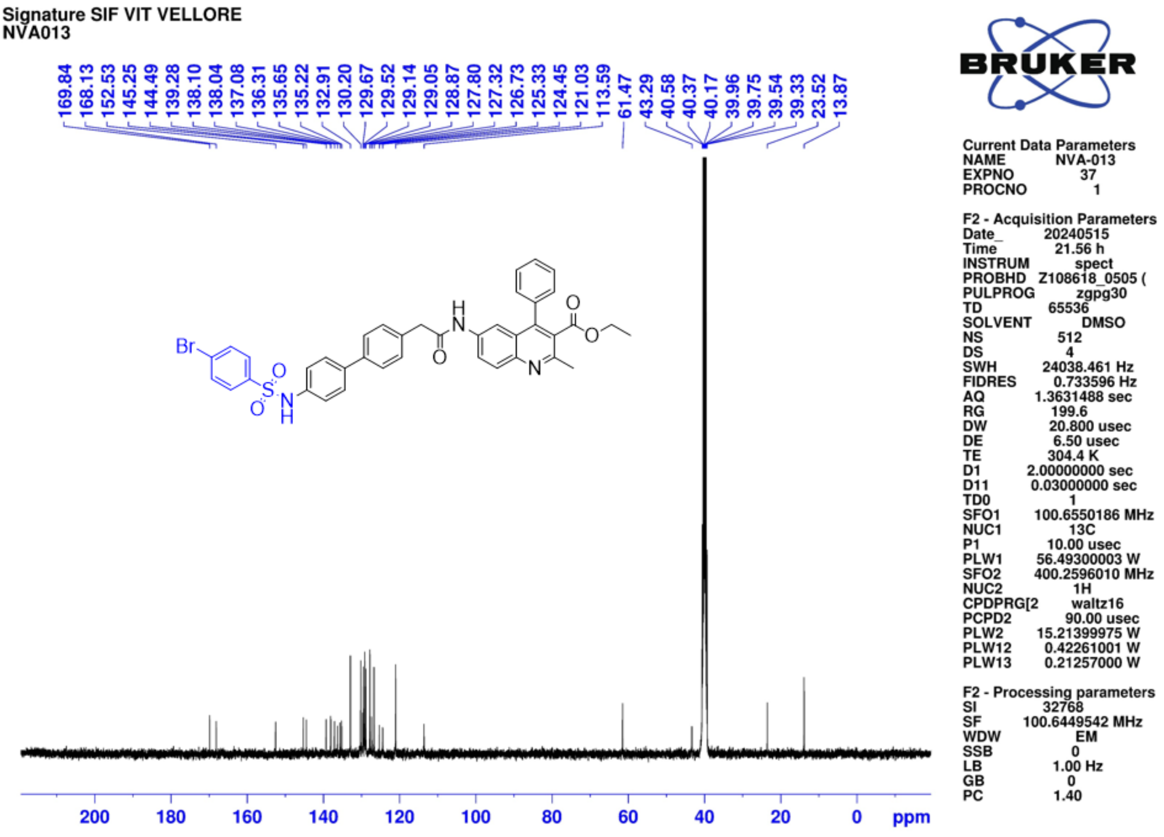

Fig. S59 ^{13}C NMR spectrum of compound 10n

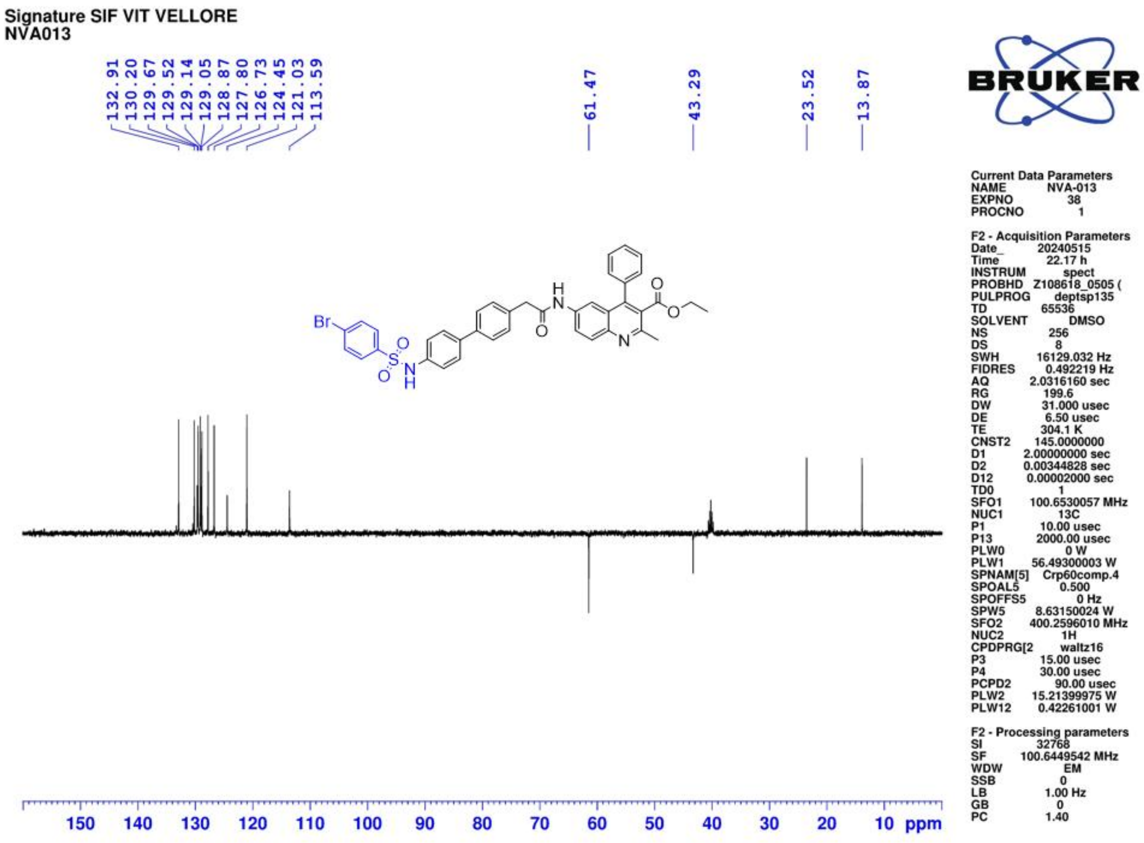

Fig. S60 DEPT-135 spectrum of compound 10n

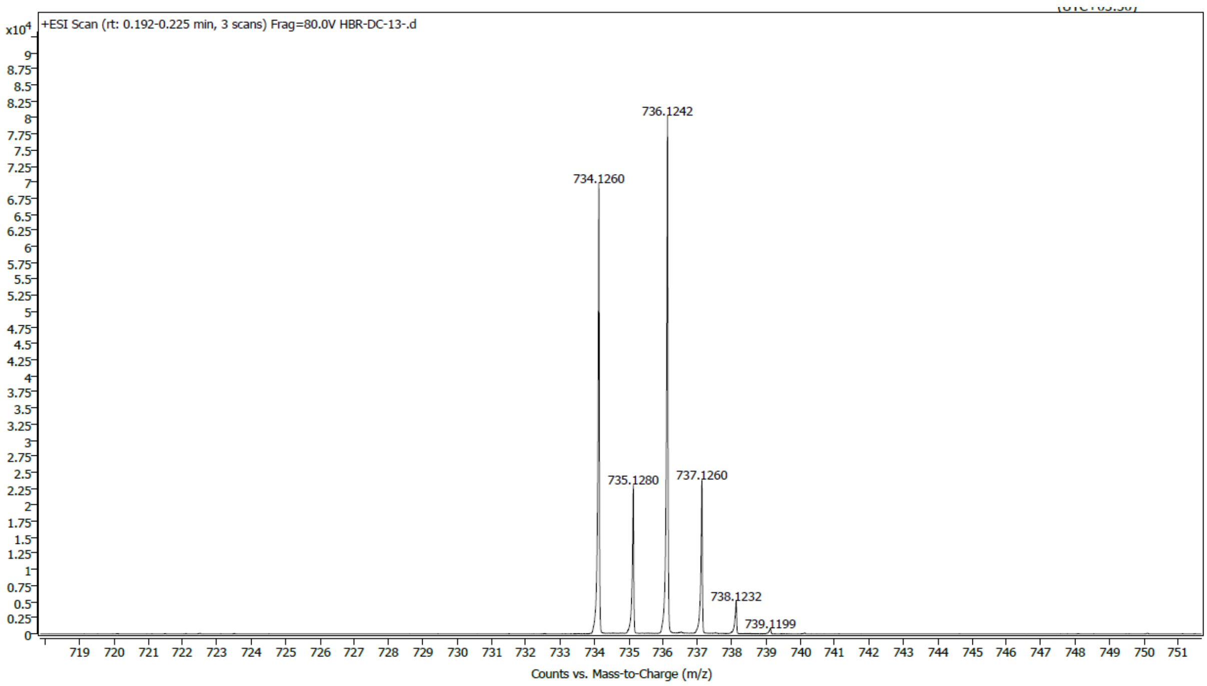

Fig. S61 HRMS of compound 10n

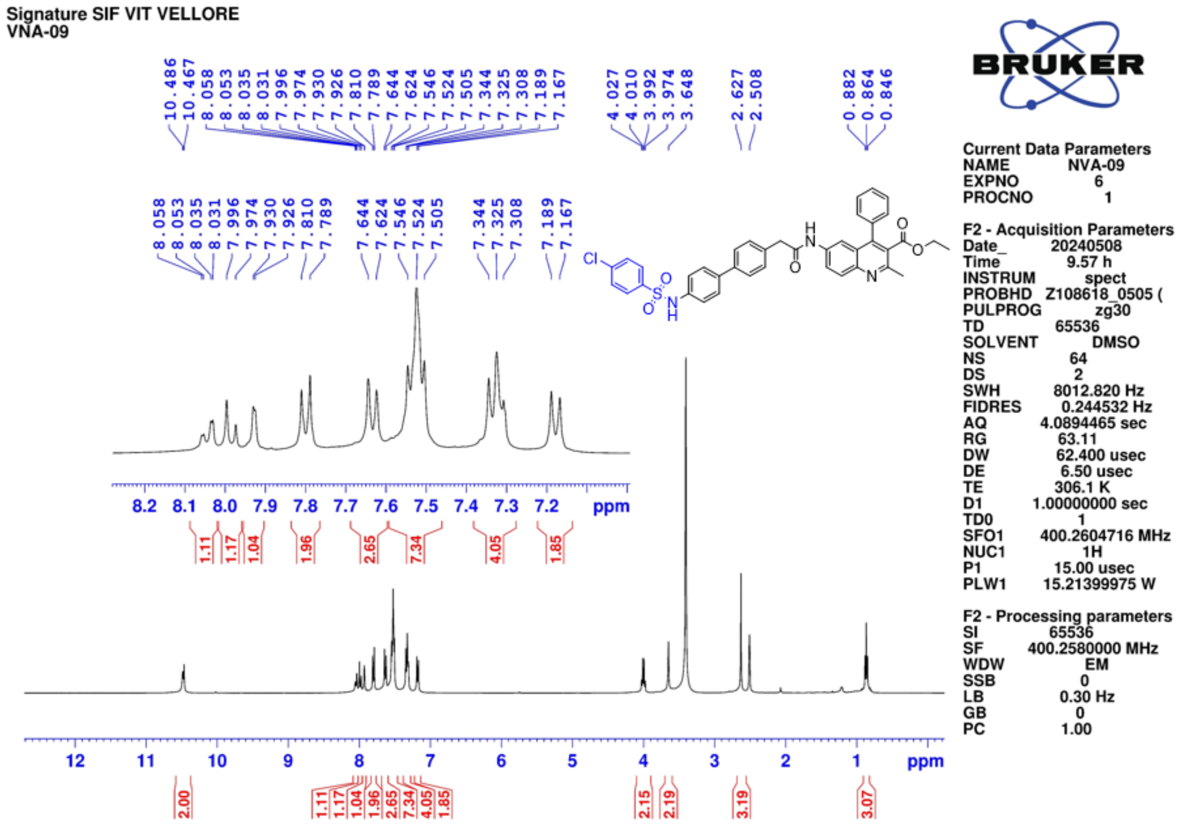

Fig. S62 ^{1}H NMR spectrum of compound 10o

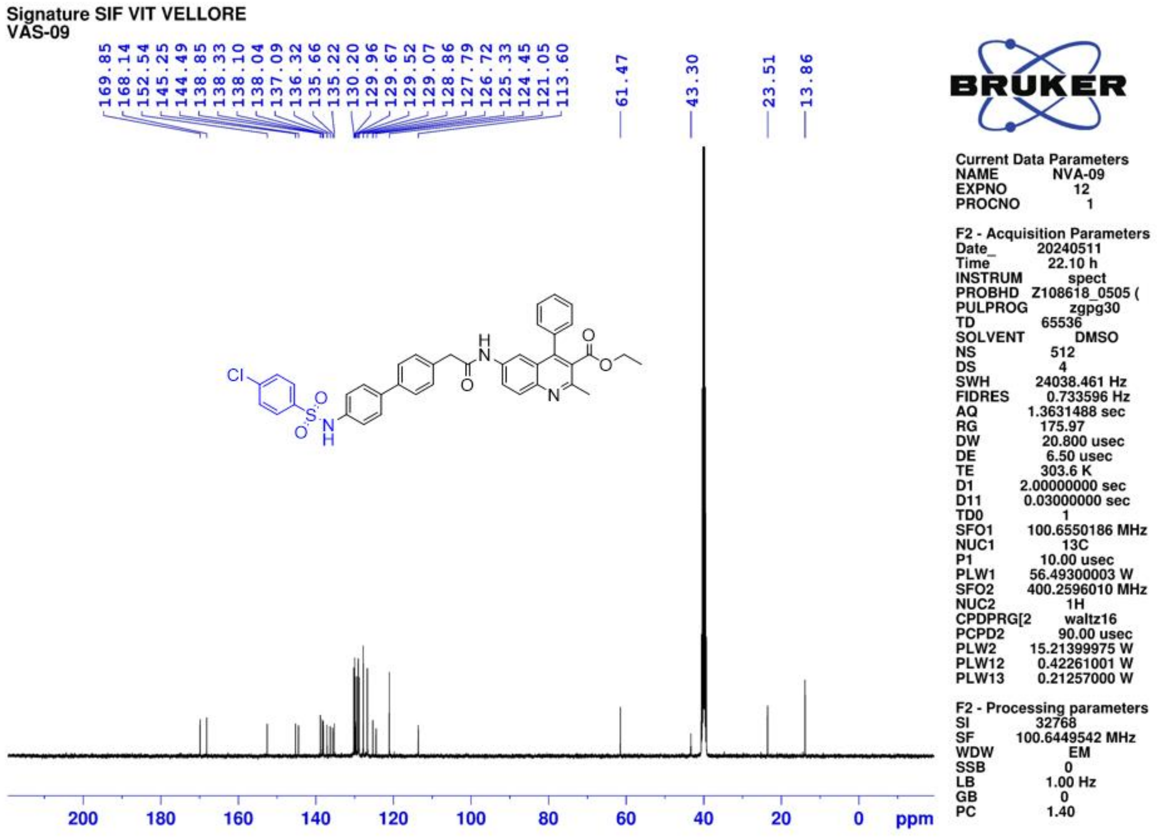

Fig. S63 ^{13}C NMR spectrum of compound 10o

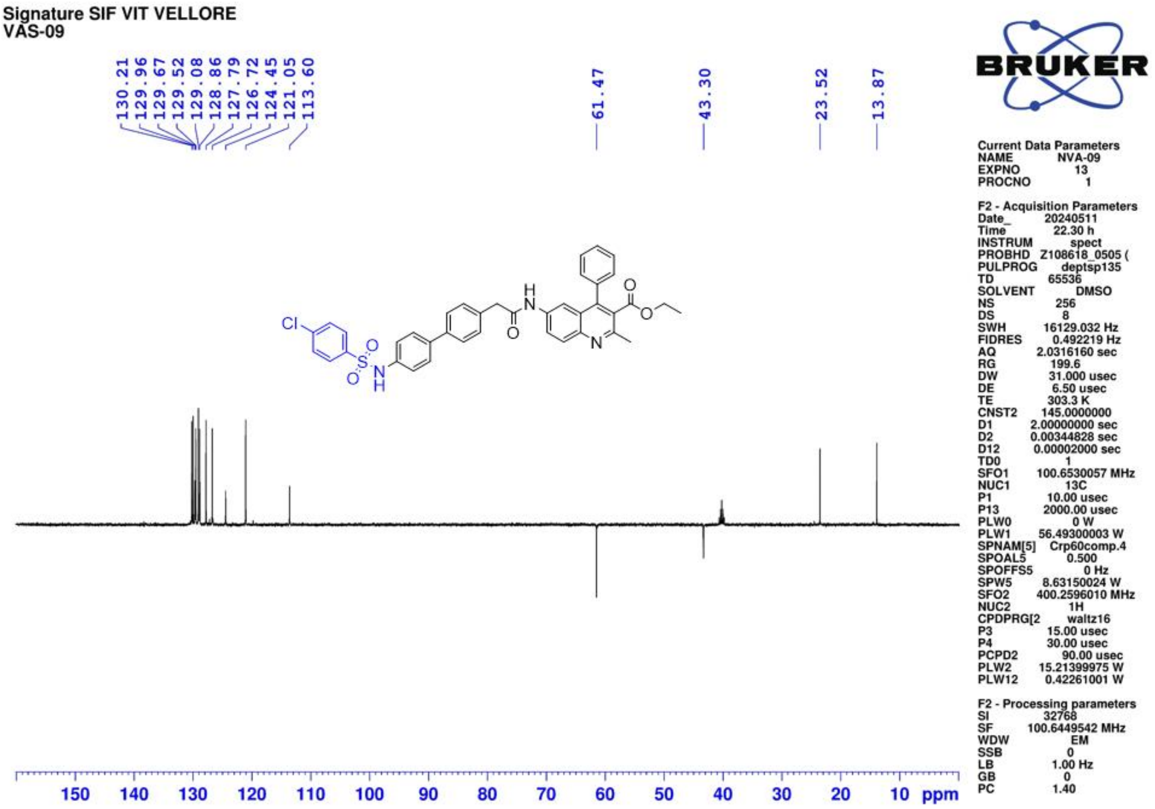

Fig. S64 DEPT-135 NMR spectrum of compound 10o

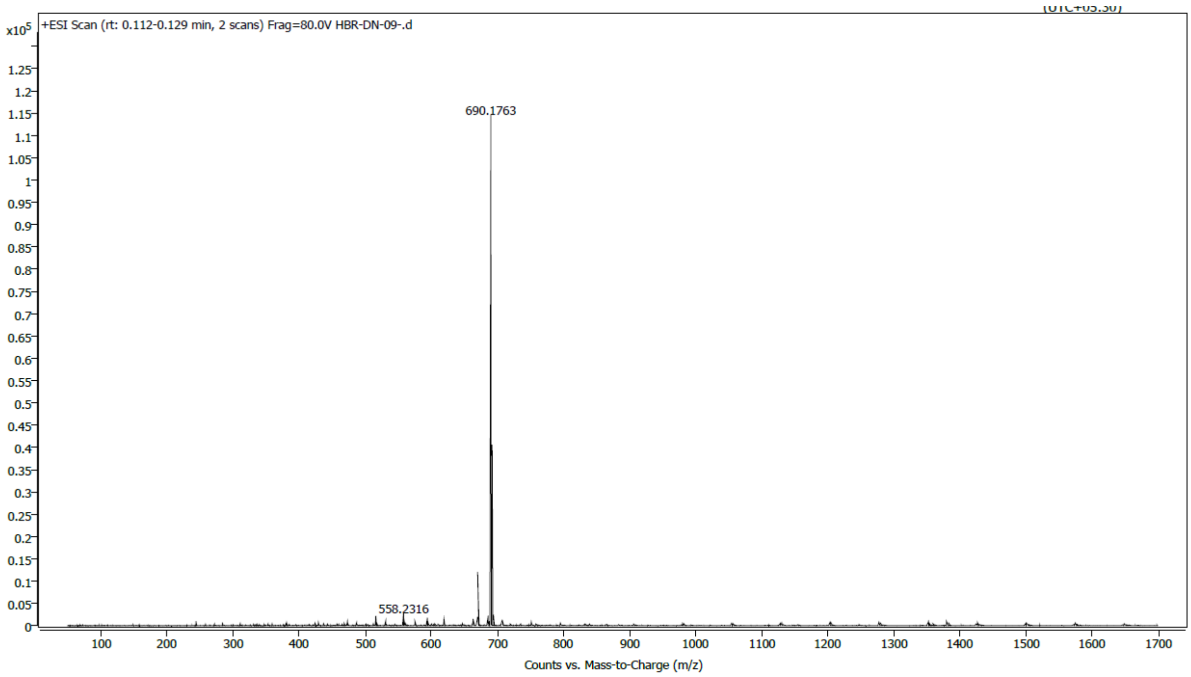

Fig. S65 HRMS of compound 10o

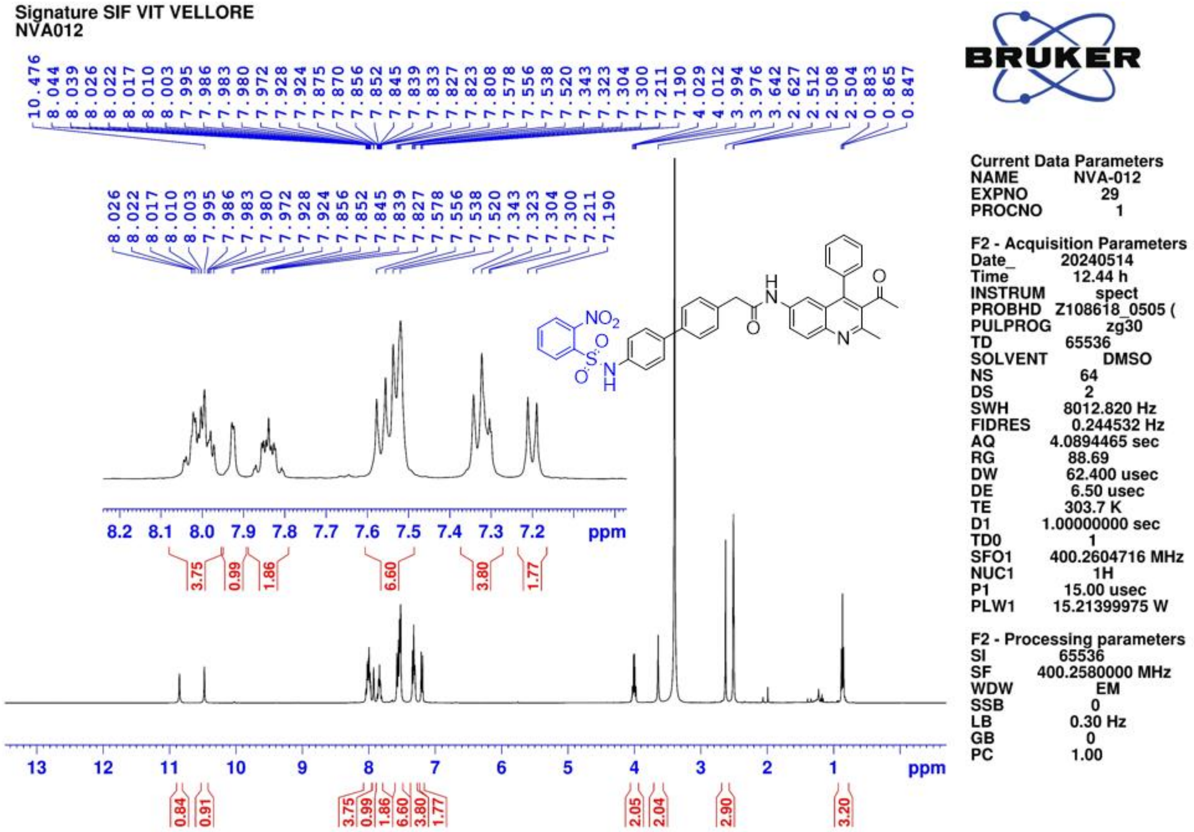

Fig. S66 ^{1}H NMR spectrum of compound 10p

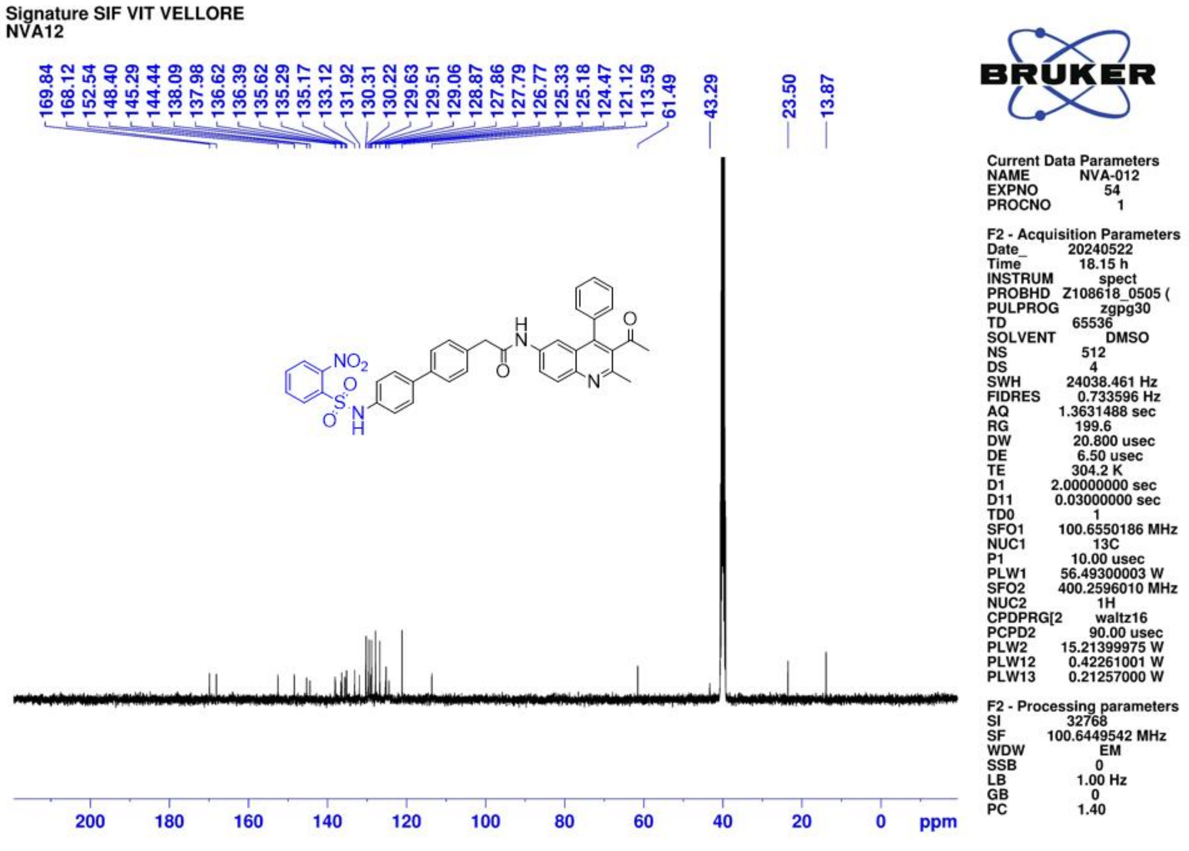

Fig. S67 ^{13}C NMR spectrum of compound 10p

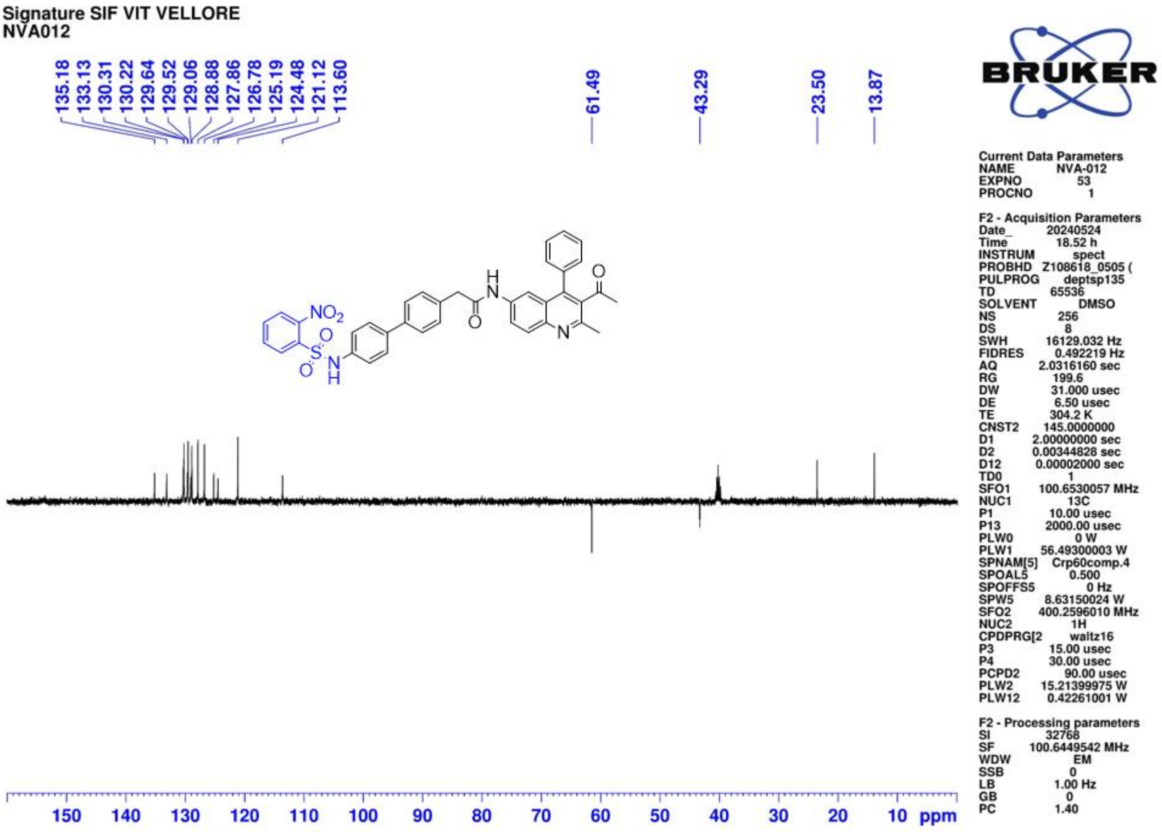

Fig. S68 DEPT-135 NMR spectrum of compound 10p
